# Risks, Epidemics, and Prevention Measures of Infectious Diseases in Major Sports Events: Scoping Review

**DOI:** 10.2196/40042

**Published:** 2022-12-02

**Authors:** Xiangyu Yan, Yian Fang, Yongjie Li, Zhongwei Jia, Bo Zhang

**Affiliations:** 1 School of Public Health Peking University Beijing China; 2 School of Basic Medical Sciences Peking University Beijing China; 3 Center for Intelligent Public Health Institute for Artificial Intelligence Peking University Beijing China; 4 Center for Drug Abuse Control and Prevention National Institute of Health Data Science Peking University Beijing China

**Keywords:** major sports event, epidemic, risk factor, prevention, surveillance, scoping review

## Abstract

**Background:**

Major sports events are the focus of the world. However, the gathering of crowds during these events creates huge risks of infectious diseases transmission, posing a significant public health threat.

**Objective:**

The aim of this study was to systematically review the epidemiological characteristics and prevention measures of infectious diseases at major sports events.

**Methods:**

The procedure of this scoping review followed Arksey and O’Malley’s five-step methodological framework. Electronic databases, including PubMed, Web of Science, Scopus, and Embase, were searched systematically. The general information (ie, publication year, study type) of each study, sports events’ features (ie, date and host location), infectious diseases’ epidemiological characteristics (ie, epidemics, risk factors), prevention measures, and surveillance paradigm were extracted, categorized, and summarized.

**Results:**

A total of 24,460 articles were retrieved from the databases and 358 studies were included in the final data synthesis based on selection criteria. A rapid growth of studies was found over recent years. The number of studies investigating epidemics and risk factors for sports events increased from 16/254 (6.3%) before 2000 to 201/254 (79.1%) after 2010. Studies focusing on prevention measures of infectious diseases accounted for 85.0% (238/280) of the articles published after 2010. A variety of infectious diseases have been reported, including respiratory tract infection, gastrointestinal infection, vector-borne infection, blood-borne infection, and water-contact infection. Among them, respiratory tract infections were the most concerning diseases (250/358, 69.8%). Besides some routine prevention measures targeted at risk factors of different diseases, strengthening surveillance was highlighted in the literature. The surveillance system appeared to have gone through three stages of development, including manual archiving, network-based systems, and automated intelligent platforms.

**Conclusions:**

This critical summary and collation of previous empirical evidence is meaningful to provide references for holding major sports events. It is essential to improve the surveillance techniques for timely detection of the emergence of epidemics and to improve risk perception in future practice.

## Introduction

Major sports events, including national or international multisport events (eg, the Olympic Games) and single-sport events (eg, International Federation of Association Football [FIFA] World Cup), attract the focus of the world. However, the gathering of crowds from various parts of the world poses challenges to public health, especially for global infectious disease prevention and control [1]. Moreover, the threats of infectious disease exist over the entire course of the games, including the competition stage and the journey before and after the sports events [2,3]. Several factors play roles in public health security of these sports events, including close contact of the attendees in confined and crowded spaces, the demographics and disease exposure history of the participants, their mobility patterns, the event setting, and climate conditions [2-4]. The incoming and outgoing travel patterns of international participants attending these events can accelerate disease transmission among a large number of people, potentially leading to a pandemic within a short period [5,6].

Many recent studies have raised concerns about the potential risks of infectious disease in major sports events and their impacts on public health on a global scale [7]. During the 1991 International Special Olympics Games, 16 outbreak-associated secondary measles cases were reported in athletes, spectators, and volunteers [7]. An outbreak of 36 influenza cases among participants was recorded at the 2002 Winter Olympics held in Salt Lake City, Utah, United States [8]. Outbreaks of norovirus were reported at many sports events such as the 2006 Football World Cup and 2018 Winter Olympics [9,10]. In addition, many other types of infectious diseases might also threaten public health security in major sports events, such as leptospirosis infection in triathlons, spread of blood-borne diseases in boxing, and small-scale transmission of insect-borne diseases at World Cup events [11-13].

To better prevent and control infectious disease outbreaks, routine prevention and control measures, new technologies for symptom surveillance, environmental sampling, and virus testing are used in major sports events. Routine prevention and control measures include reminding participants to pay attention to personal hygiene, and strengthening publicity, education, and cooperation between health institutions [14,15]. For infectious disease surveillance and management, before the 1990s, major sports events mainly used traditional methods relying on routine manual filing of reportable diseases by clinicians [16-21]. The development of network communication technology makes it possible to quickly and automatically monitor infectious diseases based on a variety of data sources, including clinical data from health providers, drug sales from pharmacies, outbreak reports, emergency and urgent care data, websites and social media posts, environmental data, and travel flights, to name a few [22-24].

Overall, previous studies have provided abundant evidence of infectious disease epidemics, risk factors, and prevention measures among major sports events around the world. However, the evidence remains very fragmented, with no study systematically summarizing the prevention and control of infectious diseases in major sporting events. To fill this gap, the aim of this scoping review was to provide an overview and critical summary of existing evidence on the epidemics and risk factors, prevention measures, and surveillance paradigm of infectious diseases in major sports events, which could help to provide guidance for the prevention and control of infectious diseases at national and international major sports events, especially during the COVID-19 pandemic.

## Methods

### Design and Search Strategy

The procedure of the scoping review followed Arksey and O’Malley’s [25] five-step methodological framework, including (1) identifying the research question; (2) identifying relevant studies; (3) study selection; (4) extraction and charting the data; and (5) collating, summarizing, and reporting the results. The results are reported in accordance with the PRISMA-ScR (Preferred Reporting Items for Systematic Reviews and Meta-Analyses extension for Scoping Reviews) guidelines [26].

A systematic search of electronic databases (PubMed, Web of Science, Scopus, and Embase) was performed from the earliest record to October 15, 2022. All study designs were included. The search strategy contained two parts: (1) the sports event and its related terms, including “athletic” or “Olympic” or “Paralympic” or “World cup” or “championship” or “marathon” or “mass gatherings” or “stadium” or “sports venues” or “gym”; and (2) terms covering topics of the infectious diseases and their transmission, including “infection” or “infestation” or “infectious” or “transmission” or “communicable disease” or “community spread” (see Multimedia Appendix 1 for the detailed search strategy).

### Study Selection

After eliminating duplicates, a two-step study selection procedure was performed. The first step was the preliminary selection through titles, abstracts, and keywords. Studies were excluded if (1) the content of the study was not relevant to infectious diseases or (2) not relevant to sports events. The second step was based on examination of the full text, and studies were excluded if (1) there was no available full text or (2) the article was not written in English. The type of article was not restricted, and comments, letters, or replies related to the research theme were all included in this review. Studies were screened independently by two reviewers (BZ, XY) against the above criteria. Disagreements were resolved through discussion or via a third reviewer (YL).

### Data Extraction

Two authors (BZ, XY) reviewed the included studies and analyzed infectious diseases in major sports events. The general information (ie, publication year, study type) was extracted. Data related to the sports events’ features (ie, type, date, and host location); key findings of each study, including infectious disease characteristics (ie, types, epidemics, risk factors), prevention measures, and surveillance paradigm of infectious diseases, were also extracted. According to the extracted data, literature quality assessment was performed based on the following two criteria: (1) specifies the type of sport and the scale of the sport event, along with the host location and time of the event; (2) specifies the main types of infectious diseases in the sports event. If either of the two criteria was not met, the study was considered to be of low quality and was excluded.

### Data Synthesis

Descriptive analysis was performed for statistics of related infectious diseases (eg, respiratory tract infection, vector-borne infection) by time period. According to the sports types, the included sports events were divided into four categories, including multisport (eg, the Olympic Games), ball (eg, football, basketball, tennis), race (eg, running, marathon, bike race), and other sports types (eg, swimming, fighting). This classification also referred to the sports categories of the world’s greatest sports events from a previous report [27]. Maps were used to present the geographical distribution of studies on different infectious diseases around the world. Key findings of the included studies were summarized and categorized into two main topics, including (1) epidemics and risk factors and (2) prevention measures, which were determined by the studies’ primary aims and outcomes, with subcategories identified where appropriate. Statistical analyses were performed using SPSS version 21.0 (IBM Corp), and Tableau 2021 (Tableau Software) was used for mapping.

## Results

### General Characteristics of Included Studies

**Figure 1:**
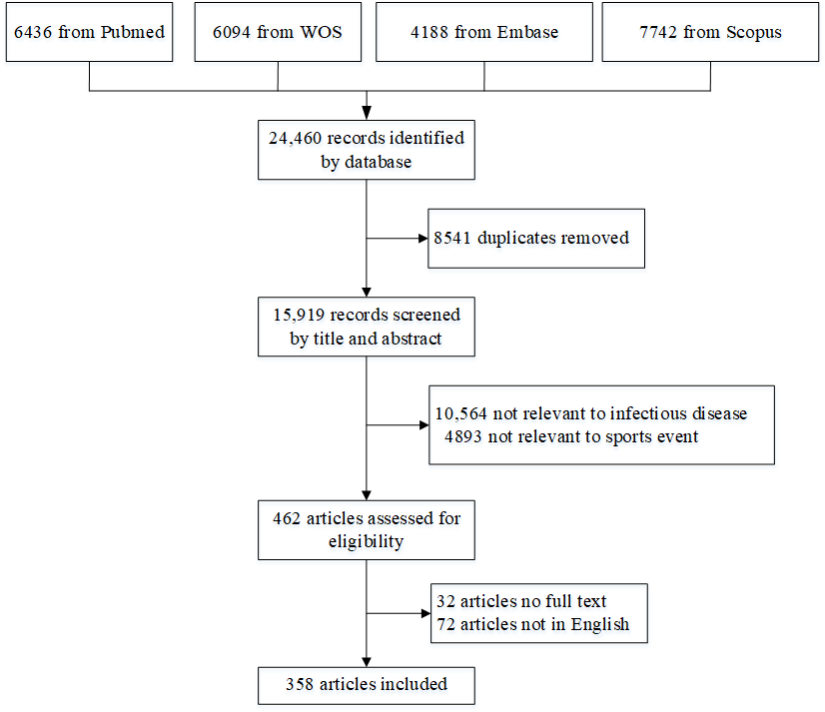
Flow of selection process for eligible studies for inclusion. WOS: Web of Science.

**Figure 2:**
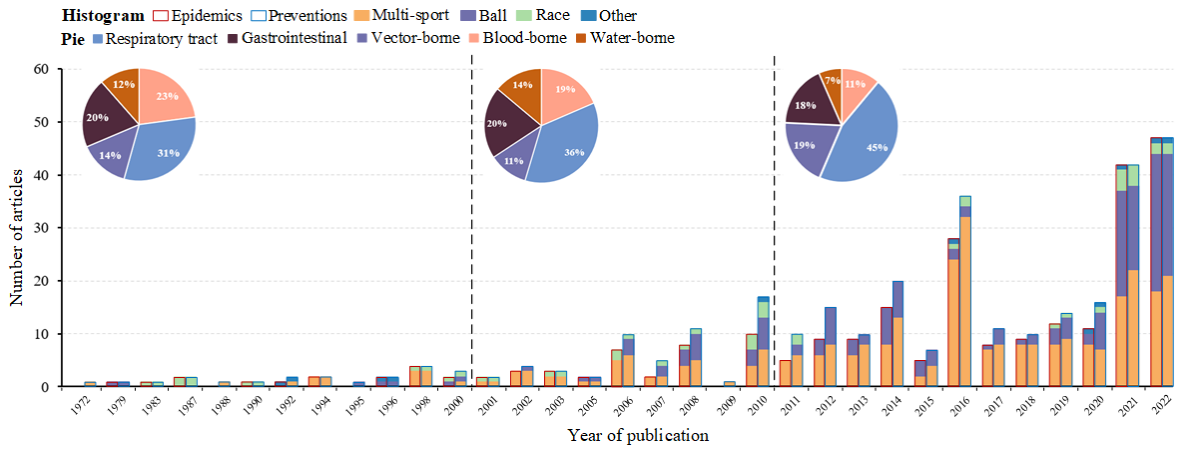
Number of studies on epidemics and prevention of infectious diseases in major sports events over time.

We identified 24,460 articles through a database search. Following the removal of duplicates and screening for eligibility, 358 studies were ultimately included in the review (Figure 1). The basic information of the included articles is provided in Tables S1 and S2 of Multimedia Appendix 1. Figure 2 shows the numbers of publication and their themes over recent years, demonstrating the rapid growth of studies in this area. Only 16 of 254 studies (6.3%) investigated epidemics and risk factors of infectious disease in major sports events before 2000, which increased to 201 (79.1%) after 2011. Studies focusing on prevention measures accounted for 85.0% (238/280) of the articles published after 2010. The main types of sports studied over the years were multisport events and ball games (Figure 2). Among the 358 studies, the top five most studied major sports events were the 2016 Rio Olympics (n=46, 12.8%), 2020 Tokyo Olympics (n=42, 11.7%), 2012 London Olympics (n=19, 5.3%), 2014 FIFA World Cup (n=16, 4.5%), and 2010 FIFA World Cup (n=14, 3.9%) (Multimedia Appendix 1 Table S3).

Based on the main transmission routes, the infectious diseases reported were classified into five categories: respiratory tract infection, gastrointestinal infection, vector-borne infection, blood-borne infection, and water-contact infection. Among them, over half (250/358, 69.8%) focused on respiratory tract infections, with COVID-19, measles, and influenza as the main concerns (Figure 2). Studies on vector-borne diseases such as Zika and dengue showed the fastest rate of increase in recent years, from 23.8% (5/21) published before 2000 to 30.4% (85/280) published after 2010 (Figure 2). Moreover, a considerable number of studies have focused on gastrointestinal infections (eg, *Salmonella*, norovirus), blood-borne infections (eg, HIV/AIDS, hepatitis B), and water-contact infections (eg, leptospirosis) (Figure 2).

**Figure 3:**
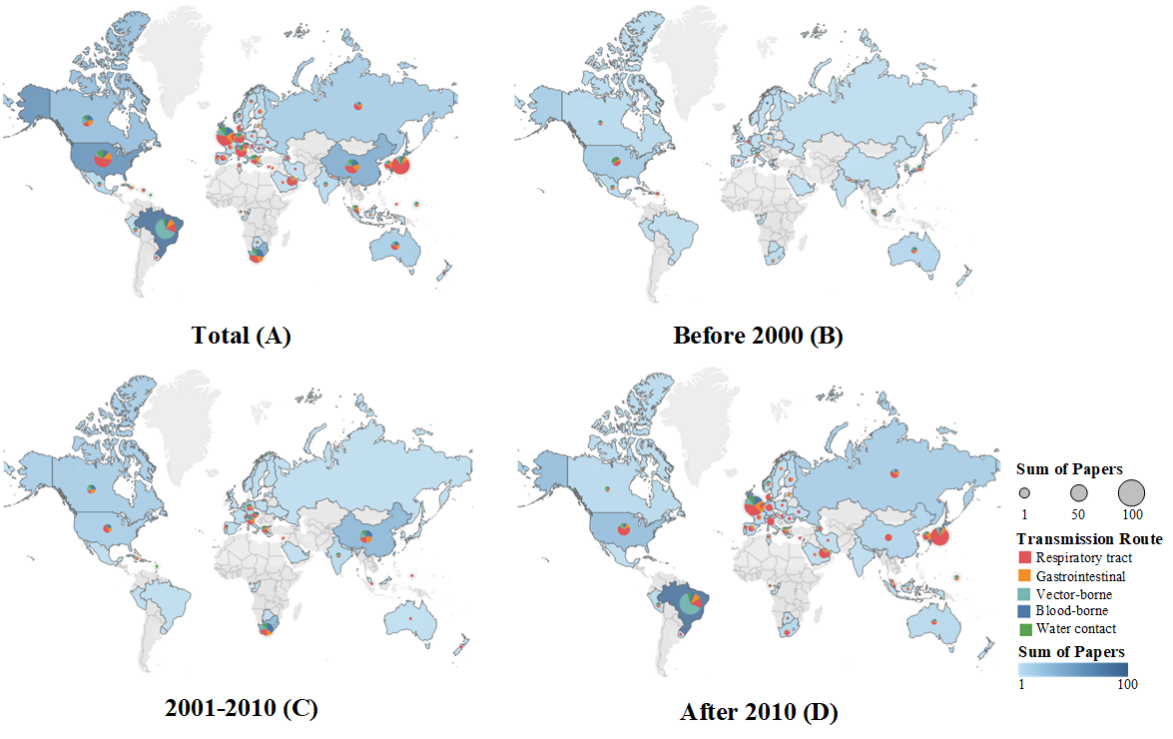
Geographical distribution of studies on infectious diseases in major sports events.

Several studies focused on the major international sports events’ host countries (Figure 3A). The 1996 Olympics held in Atlanta, Georgia, United States; the 2000 Olympics in Sydney, Australia; the 2008 Olympics in Beijing, China; and the 2010 FIFA World Cup in South Africa were the key events of focus of the corresponding periods (Figure 3B, C). After 2010, based on the Olympics and the FIFA World Cup, most studies concentrated in Brazil, the United Kingdom, Japan, and China (Figure 3D). Among them, studies related to Brazil mainly focused on vector-borne infections, as the 2014 FIFA World Cup and the 2016 Olympic Games both took place in Brazil where Zika, dengue, and other vector-borne infections were more prevalent, whereas COVID-19 has been the focus of the Tokyo and Beijing Olympics and Paralympics (Figure 3D).

### Epidemics and Risk Factors

#### Respiratory Tract Infections

Respiratory tract infections such as influenza and measles are the most common infections in major sporting events. In the 2018 Winter Olympics, Team Finland reported that respiratory tract infections caused by coronaviruses, influenza B virus, respiratory syncytial virus A, rhinovirus, and human metapneumovirus affected 45% of the athletes and 32% of staff members of the service team [28]. Measles is also a highly infectious and acute viral respiratory tract infection that caused small outbreaks in sports events such as the 1991 International Special Olympics Games, 1991 International Gymnastics Competition, and 2007 International Youth Sporting Event [7]. At the end of 2019, the sudden outbreak of COVID-19 disrupted the pace of human life, and many sports events were forced to be cancelled or postponed. Thailand first reported SARS-CoV-2 transmission in a boxing sport stadium, with 27 individuals infected [29]. During the Tokyo Olympics and Paralympics, the positive rates of COVID-19 tests were 0.10% and 0.03% for games-related people arriving at airports and people living in the Tokyo 2020 bubble, respectively, including 24 of 11,476 Olympic athletes and 13 of 4303 Paralympic athletes who tested positive [30]. As for the Beijing 2022 Winter Olympics and Paralympics, 284 of the 16,092 games-related people tested positive at the airport and 179 people tested positive in the closed loop with no cluster epidemic [31] (see Tables S1 and S2 of Multimedia Appendix 1).

**Figure 4:**
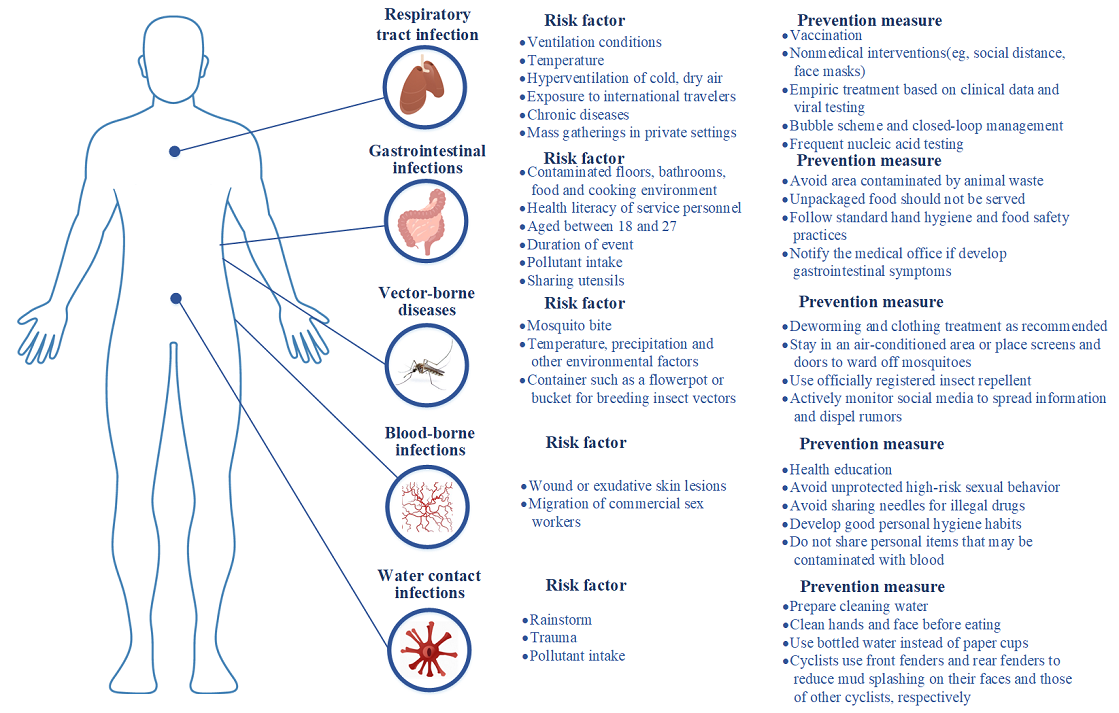
Risk factors and prevention measures in major sports events.

Risk factors for respiratory infectious diseases have mainly included three aspects. First, respiratory tract infections could easily be transmitted in confined places; therefore, ventilation conditions, temperature, cold, and dry air in the competition grounds were highlighted as risk factors for respiratory tract infections, because these conditions are suitable for viruses to survive and colonize the human respiratory tract [8]. Second, individuals with routine exposure to international travelers might be at greater risk of respiratory tract infections [32]. Third, people with chronic diseases or weak immunity are more susceptible to respiratory tract infection, as well as its aggravation or complications [32] (Figure 4).

For the ongoing COVID-19 epidemic, the emerging viral variants and the relatively low vaccination rate brought challenges to the holding of major sports events [33]. When holding the Tokyo Olympics, only 3.4% of the population in Japan had been immunized [33]. Another risk for the spread of COVID-19 is the uncontrolled gathering for activities occurring outside the venue during the sports events. Two studies indicated that the increase in COVID-19 cases and cluster epidemics during the 2020 European Football Championship (EURO) were more likely to occur as a result of aggregations and celebrations in private settings, pubs/other public buildings, or public squares rather than from the official football events [34,35]. In addition, previous studies found that athletes had a higher risk of COVID-19 than staff members during professional football competitions [36,37] (Figure 4).

#### Gastrointestinal Infection

Norovirus and *Salmonella* were the main pathogens that caused gastrointestinal infections in major sports events. In the 2015 Obstacle Adventure race, 866 of 1264 adults reported acute gastroenteritis infections [38]. In the 2018 PyeongChang Winter Olympics, symptoms associated with the gastrointestinal tract, including diarrhea, vomiting, and dyspepsia, were the second most common reported [10]; an outbreak of norovirus infections was recognized, starting with security staff, and noroviruses were detected in samples from food handlers [39] (Multimedia Appendix 1 Tables S1 and S2).

Dirty floors and bathrooms, contaminated food and unhygienic cooking, and serving staff with little awareness of hygiene were all highlighted as risk factors for gastrointestinal infections [38]. Younger athletes (aged 18-27 years) had a significantly higher risk of acute gastrointestinal infection [38]. Ingestion of mud during sports events was associated with gastrointestinal infection [38]. In addition, the pathogens could be transmitted by sharing water bottles or food boxes [40] (Figure 4).

#### Vector-Borne Infections

During the 2014 FIFA World Cup and the 2016 Rio Olympics Games, vector-borne diseases such as Zika and dengue aroused wide public concern. Due to the efforts of implementing highly effective preventive measures against vectors, there were no epidemic incidents during either event in Brazil [41,42] (Multimedia Appendix 1 Tables S1 and S2).

The primary vectors of Zika, *Aedes* mosquitoes, breed larvae in standing water. Potential mosquito breeding sites (eg, tires, flowerpots, buckets, and other similar containers) were highlighted as risk factors for disease spread [43]. In addition, temperature, precipitation, and other environmental factors had an impact on vector-borne disease infections [44,45] (Figure 4).

#### Blood-Borne Infections

There was no statistically significant increase in clinic attendance for blood-borne infections (eg, HIV, hepatitis B virus, or hepatitis C virus infections) during sports events in previous years. Sexual transmission was the main transmission route for such infections. The distribution of condoms and health-related messages about safer sex might have contributed to the successful control of blood-borne infections during major sports events [46-48] (Multimedia Appendix 1 Tables S1 and S2).

Injuries and close contacts during competition were highlighted as risk factors for blood-borne pathogen transmission. The pathogen could transmit through bleeding wounds or exudative skin lesions of an infected athlete to the injured skin or mucous membrane of another athlete [12]. Migration was also an important risk factor. Internal and international migration of commercial sex workers might become an important vector for the transmission of HIV [3] (Figure 4).

#### Water-Contact Infections

The largest outbreak of leptospirosis was reported in the 1998 Springfield triathlon, held in the United States. Among 834 athletes in the Springfield triathlon, 98 (11.8%) met the definition for a suspected case [11] (Multimedia Appendix 1 Tables S1 and S2). Heavy rains that preceded the triathlon were likely to cause leptospiral contamination of the lake. Urine from small mammals (eg, rodents such as mice and rats), wild boars, or domestic pigs was proposed as a possible source of *Leptospira*. Swimming or wading in fresh unchlorinated water containing contaminated animal urine was another potential cause of leptospirosis [11,49,50] (Figure 4).

### Prevention Measures

#### Routine Preventive Measures

Vaccination emerged as the most effective preventive measure. All delegation members, staff, volunteers, and accompanying visitors should be appropriately vaccinated according to the recommendations of their respective nations before arrival [51,52]. Under the influence of a global pandemic of infectious diseases, local and state health departments should work quickly with organizing committees and governing bodies to establish plans for evaluation, treatment, and prophylaxis, and to ensure that vaccination clinics are functioning appropriately [51] (Figure 4, Multimedia Appendix 1 Tables S1 and S2).

The strategy for respiratory tract infections prevention can integrate empiric treatment based on clinical data and viral testing, along with a public health surveillance approach, including daily review of all viral test results from the polyclinic and reports of symptoms in the community [8]. Nonpharmacological interventions such as social distancing, facemask wearing, and ventilation efficiency enhanced in the stadium are also needed to minimize the risk of community transmission [53,54] (Figure 4, Multimedia Appendix 1 Tables S1 and S2). To prevent COVID-19 transmission, no spectators were allowed and a bubble scheme for games-related people was implemented in some international sports events represented by the Tokyo Olympics and Paralympics, which consisted of a series of measures that separated participants from the general public [55]. Such a closed-loop management strategy was also implemented in the Beijing Winter Olympics and Paralympics [56]. Another strengthening preventive measure was frequent SARS-CoV-2 testing. During the Tokyo Olympics and Paralympics, all participants had to undergo laboratory-based saliva antigen screening every day, and relevant isolation and close-contacts management were implemented for people with positive nucleic acid testing results [30,55]. In addition, it is strongly recommended that games-related people should be vaccinated against COVID-19. More than 80% of athletes and staff were vaccinated in the Tokyo Olympics and Paralympics [55].

For gastroenteritis infections, standard hand hygiene and food safety precautions were recommended, such as eating appropriately cooked food and drinking bottled beverages. Participants were also advised to notify medical offices if gastrointestinal symptoms appeared during or after the competition [23]. In the meantime, the awareness and education of the public as well as of health care professionals are warranted [57] (Figure 4, Multimedia Appendix 1 Tables S1 and S2).

The mainstay for vector-borne infections prevention is to avoid mosquito bites [43,58]. Participants must take extra care to follow recommendations from the sports event organizing committee for insect repellant and clothing treatment [59]. Some equipment was recommended, including rooms with air conditioning, window and door screens, mosquito bed nets, and official registered insect repellents, which are proven to be safe and effective [43] (Figure 4, Multimedia Appendix 1 Tables S1 and S2).

Emphasis on the prevention of transmission of blood-borne pathogens in athletes should focus on reducing the traditional modes of transmission and modification of off-the-field behavior, including the avoidance of unprotected high-risk sexual activity and needle sharing in the use of illicit drugs such as anabolic steroids or blood doping [60,61]. Athletes should also practice good personal hygiene and not share personal items that might be contaminated with blood, such as razors, toothbrushes, and nail clippers [60,61]. In addition, education regarding universal precautions when dealing with blood or body fluids should also be enhanced [60,61] (Figure 4, Multimedia Appendix 1 Tables S1 and S2).

To prevent water-contact infections in eco-challenge and racing competitions, racers and organizers should be aware of the potential risk of inadvertent ingestion of muddy and possibly contaminated water during the race [62]. In general, planners of these competitions should consider building circuits to avoid areas heavily contaminated with animal feces [62]. In addition, clean running water should be available at stations to allow racers to clean mud off their hands and faces prior to eating and drinking [57]. An alternate form of water delivery should also be considered, such as bottles of water, which are less easily contaminated than paper cups [57]. It is also recommended that bike racers use front and rear fenders to reduce splashing of mud onto their and other riders’ faces, respectively [57] (Figure 4, Multimedia Appendix 1 Tables S1 and S2).

#### Surveillance Paradigm

Before the 1990s, major sports events mainly used traditional methods for disease surveillance. Traditional disease surveillance relied on routine manual filing of reportable diseases by clinicians [16-21]. With the continuous development of information technology, infectious disease surveillance technology in major sports events has gone through three stages of development.

**Figure 5:**
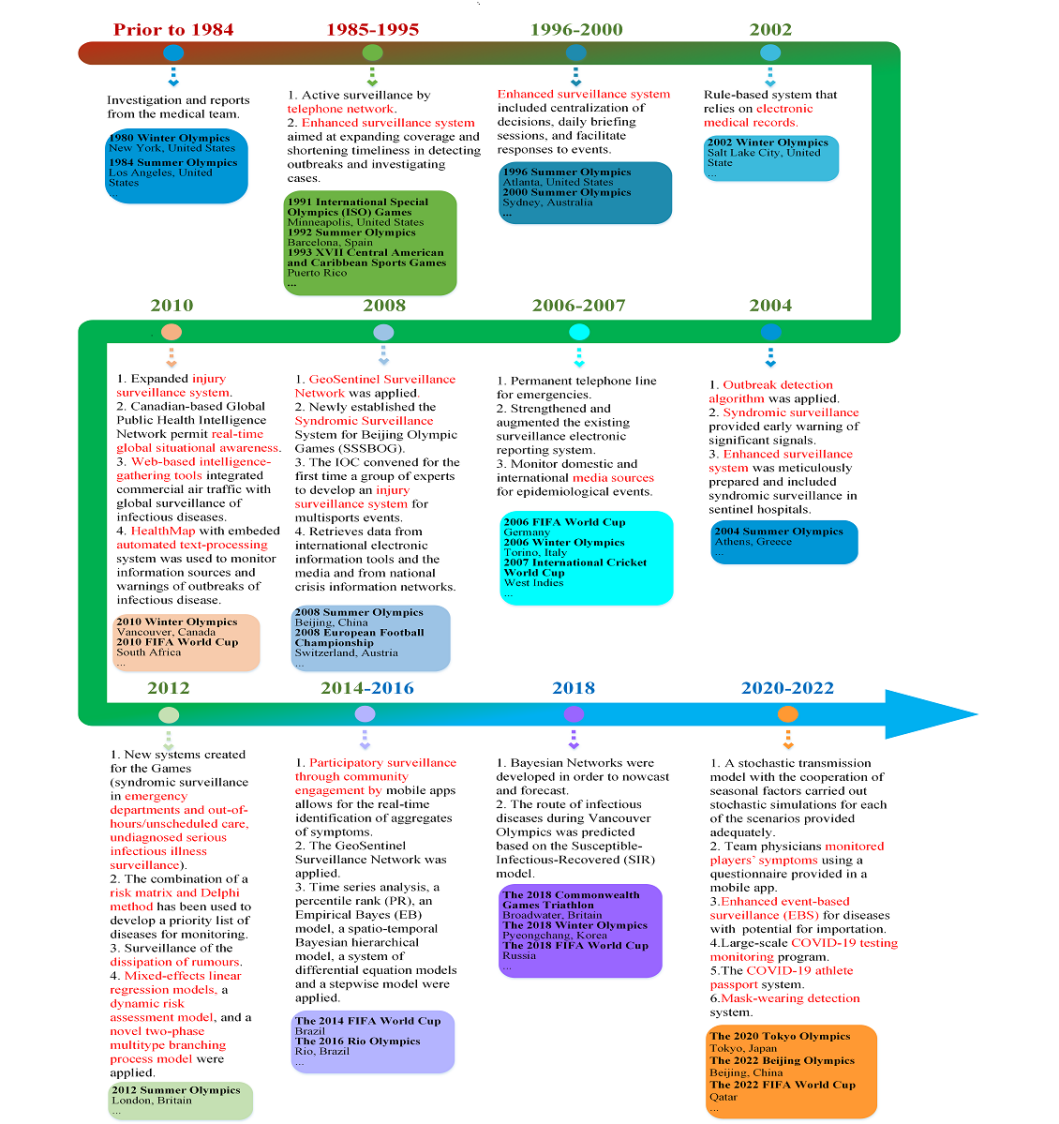
Surveillance patterns in major sports events. FIFA: International Federation of Association Football; IOC: International Olympic Committee.

The first generation of a complete disease surveillance network for major sports events was applied in the 1984 Los Angeles Olympic Games. The whole network covered 46 hospitals, 90 physician offices, 4 university student health centers, 31 preschools, 3 Olympic Village polyclinics, and 24 Olympic first-aid stations [63]. During the Games, the International Olympic Committee made three phone calls per week with 198 participating sites. Each site used a disease report card to report disease information [63]. Early active surveillance systems reported structured and simple data, mainly including the name of surveillance sites, type of surveillance sites, date, and number of cases (grouped by disease type and age of patients) [64-66] (Figure 5).

After 1995, the second generation of surveillance networks emerged with extensive application of internet technology in infectious disease surveillance that enhanced existing systems and integrated multisource data with richer data types that significantly improved real-time performance. During the 1996 Atlanta Olympic Games, the government designed and implemented “outside the fence” and “inside the fence” surveillance systems. The “outside the fence” system was an augmented surveillance system implemented for health conditions that occurred outside of Olympic venues, while the “inside the fence” surveillance system was established to enumerate clinical encounters in Olympic venues and at contract hospitals for Olympic athletes, official Olympic staff, and national delegations [67]. Continuous surveillance data collection allowed uninterrupted monitoring of disease epidemic trends.

In the following major sports events (eg, 2000 Sydney Olympics, 2006 Germany FIFA World Cup, 2007 International Cricket World Cup, 2008 EURO, 2008 Beijing Olympics), the infectious disease surveillance system continued using this mode, with two major improvements, including enhancing the existing domestic surveillance system and establishing syndromic surveillance targeting health-related symptoms [4-6,68,69]. Data structures and types were enriched in the form of electronic medical records. Automatic data acquisition technology improved the timeliness of information collection and reporting. Complex algorithms and models were used to predict outbreaks of infectious diseases. Several professional global surveillance networks (eg, GeoSentinel, Healthmap) were set up, collecting clinician-based sentinel surveillance data (diagnosis, travel itinerary, demographics) to support the prevention and control of infectious diseases in major sports events [22,70].

After 2016, mobile internet apps and artificial intelligence algorithms were applied to monitor participants’ health status in sports events. In the 2016 Rio Olympics, a surveillance platform based on a mobile app was installed on participants’ smartphones called “OlymTRIP,” which monitored health status through a daily interactive check of user health status, including geolocalization data [71]. The app also provided information and advice about Zika infection. In case of feeling unwell, participants could contact doctors through the app, and doctors could also track the health status of the participants in a real-time manner using a web-based platform.

In addition to mobile phones, there are many types of wearable devices for infectious disease monitoring. Using an agent-based model, the data collected by wearable devices were applied to explore the feasibility of using tracking data from a football match to assess interpersonal contact between individuals by calculating two measures of respiratory exposure [72]. More multisource data and more advanced technologies were integrated into the monitoring system, including online public opinion monitoring based on natural language processing technology, risk classification monitoring of international passengers based on global aviation data, and the risk early warning model of infectious diseases [22,24,73]. In addition, enhanced event-based surveillance was undertaken by the National Institute of Infectious Diseases, Japan, to identify infectious diseases occurring overseas (excluding COVID-19) that had potential for importation during the Tokyo Olympics [74].

In response to the challenges of COVID-19, some surveillance measures focusing on COVID-19 were developed and implemented. The COVID-19 athlete passport system was used during the Tokyo Olympic games to present a novel way of managing athletes’ previous exposure, testing results, and vaccination status, which could be used to tailor appropriate precautions to each athlete [75]. A large-scale COVID-19 monitoring program involving daily testing was instituted during the US 2020 National Football League season [76]. For enhanced surveillance activities during EURO 2020, a specific identifier was marked in the system for each case within the observation period that might have had an association with the games to facilitate communication and timely case detection by local health authorities [77]. In addition, some image-based technologies of mask-wearing detection could contribute to monitoring the athletes’ and spectators’ mask-wearing behaviors during sports events [78] (Figure 5).

## Discussion

### Principal Findings

The most prominent characteristics of major sports events are mass gathering and cross-regional mass mobility, which may contribute to a pathogen’s cross-regional, national, and even continental transmission. In recent years, more and more research on this topic has emerged, with a 13-fold increase in the number of research papers published since 2010 compared to that before 2000. There were a variety of infectious diseases of concern during major sporting events. Among them, the most predominant infectious diseases were respiratory tract infections, followed by gastrointestinal infections, vector-borne infections, blood-borne infections, and water-contact infections. It was noteworthy that massive outbreaks of infectious diseases in major sports events were generally scarce, mainly due to thorough prevention and control measures.

Major sports events are the hotbed of almost all types of infectious diseases. Therefore, in the process of prevention and control of infectious diseases in sports events, targeted prevention and control measures should be taken according to the main risk factors of infectious diseases. For vaccine-preventable diseases, routine vaccination coverage should be improved. The environmental conditions of the sports events venue should be monitored to protect the upper respiratory tract of participants. Food and drinking-water hygiene should be ensured to reduce the risk of gastrointestinal infection. For vector-borne diseases, the hidden danger of breeding insects should be eliminated. For blood-borne diseases, especially sexually transmitted infections, health education should be strengthened to reduce risky sexual behaviors. For water-contact infectious diseases prevention, bacteria and microorganisms in the water should be monitored to eliminate the risk of infection, and providing clean water for racing is essential. Previous studies have mainly focused on the disease epidemic and prevention measures for athletes and related personnel in host countries during sports events, whereas few studies have provided evidence on the follow-up monitoring of returning participants after major sports events. In future research, participating countries should strengthen health status monitoring of sports events–relevant individuals after they return, and, if necessary, form a closed loop for management to prevent transnational epidemics.

The control of infectious diseases in major sports events cannot be separated from the support of surveillance technology. The number of studies on surveillance of infectious diseases in major sports events in recent years also presented explosive growth. Additionally, improvements in technology (eg, mathematical modeling, big data, and artificial intelligence) have enhanced the ability to monitor the risk factors related to multiple key points during the epidemic course of infectious disease, and to realize early warning and prediction of infectious disease outbreaks. However, the application of virtual reality, big data, artificial intelligence, and other technologies in the prevention and control of COVID-19 in major sports events is still in its infancy, and the platform architecture and application mode of emerging technologies warrant further research. In the future, intelligent surveillance should be equipped with advanced tools to collect multisource heterogeneous global mass data in real time, and detect and warn about various risk factors through intelligent algorithms and models so as to realize the advance of the prevention point. The target of the development of surveillance technology in future major sports events is to realize the rapid identification of new outbreaks of emerging infectious diseases and achieve the goal of gathering data faster than the epidemic situation.

Currently, we are nearly 3 years into the COVID-19 pandemic, resulting in many sports events having been suspended, postponed, or scaled back, which are now being gradually resumed. Based on the experiences of the Tokyo and Beijing Olympic Games, strict management measures, represented by a bubble scheme, closed-loop management, and frequent testing, could effectively prevent the spread of COVID-19 in the Olympic Village and the communities of host cities during the sports events [30,31]. However, international spectators were not allowed to attend the previous two Olympic Games. The gathering of spectators from all over the world, mutation of SARS-CoV-2, and the loosening of COVID-19 prevention and control policies in many countries are still putting pressure on the efficiency and safety of holding the upcoming World Cup in Qatar and other international sports events.

In the last year, the emergence of monkeypox outbreaks around the world has also posed challenges to the holding of international sports events. The transmission of monkeypox could occur through multiple routes, including close contact with respiratory droplets, infected lesions, body fluids, contaminated materials, and sexual behaviors, and the virus has a long incubation period [79]. Therefore, researchers are warning organizers and participants of the upcoming FIFA World Cup 2022 in Qatar to pay attention to monkeypox prevention and control, and to establish reliable communication channels among health authorities, visitors, and the local population [79]. Thus, the prevention and control of the risk of infectious diseases in major sports events is a continuously evolving topic. With the emergence of new infectious diseases and the change of pandemic risk of existing infectious diseases in host and participants countries, researchers, organizers, and participants of major sports events should always be vigilant.

### Study Limitation

There is one key limitation of this study. Articles written in languages other than English were not included in this study, whereas relevant information about infectious diseases related to sports events might be published in the local media in the native language of the host countries. However, most major international sports events (such as the World Cup and Olympic Games) involve publications written in English. Our aim was to provide an overview and critical summary of existing evidence on infectious diseases in major sports events, and these English articles could cover this content. Although there may be some publications published in the local language in host countries of sports events, the key information of the sports events might not be notably missing if these publications were not included.

### Conclusion

This scoping review provides an overview and critical summary of existing evidence on the risk factors, epidemics, prevention measures, and surveillance paradigm of infectious diseases in major sports events. We observed an increase in relevant research in recent years, with international sports events such as the Olympics and FIFA World Cup as the main focus. A variety of infectious diseases of concern during major sporting events were identified, including respiratory tract infections, gastrointestinal infections, vector-borne infections, blood-borne infections, and water-contact infections, among which respiratory tract infections were the most common. Surveillance of infectious diseases during major sports events has made great progress in recent years. In particular, progress has been made to update the surveillance paradigm from a manual archive to a network-based system, and finally to automated intelligent platforms gathering multisource data that are analyzed in a timely manner by multiple algorithms and mathematical models. In the future, it will be essential to strengthen the monitoring of various risk factors of infectious diseases, improve the accuracy of intelligent algorithms and models, and ensure the timely detection of the emergence of an epidemic to effectively mitigate the risk.

## Appendix

Multimedia Appendix 1 Search strategy and detailed study characteristics (Tables S1-S3).

## Abbreviations

EURO: European Football Championship
FIFA: International Federation of Association Football
PRISMA-ScR: Preferred Reporting Items for Systematic Reviews and Meta-Analyses extension for Scoping Reviews
