# Supplementary material for: Risks, Epidemics, and Prevention Measures of Infectious Diseases in Major Sports Events: Scoping Review
Source: JMIR Public Health Surveill. 2022 Dec 2;8(12):e40042. doi: 10.2196/40042 (PMC9758642; doi:10.2196/40042)
Supplement: Multimedia Appendix 1 [file publichealth_v8i12e40042_app1.docx]

**Multimedia Appendix 1**

**Search Strategy**

**Pubmed:**

((((((((((((((((((((((("Pathogen Transmission"[Title/Abstract]) OR ("Transmission, Pathogen"[Title/Abstract])) OR ("Transmission, Infectious Disease"[Title/Abstract])) OR ("Infectious Disease Transmission"[Title/Abstract])) OR ("Communicable Disease Transmission"[Title/Abstract])) OR ("Infection Transmission"[Title/Abstract])) OR ("Transmission, Infection"[Title/Abstract])) OR ("Transmission of Infectious Disease"[Title/Abstract])) OR ("Horizontal Transmission of Infection"[Title/Abstract])) OR ("Infection Horizontal Transmission"[Title/Abstract])) OR ("Community Transmission"[Title/Abstract])) OR ("Community Transmissions"[Title/Abstract])) OR ("Community Spread"[Title/Abstract])) OR ("Person-to-Person Transmission"[Title/Abstract])) OR ("Person to Person Transmission"[Title/Abstract])) OR ("Transmission, Person-to-Person"[Title/Abstract])) OR ("Autochthonous Transmission"[Title/Abstract])) OR ("Transmission, Autochthonous"[Title/Abstract])) OR ("Close-Contact Transmission"[Title/Abstract])) OR ("Close Contact Transmission"[Title/Abstract])) OR ("Transmission, Close-Contact"[Title/Abstract]))) OR ((Infections[Mesh]) OR ((Infection[Title/Abstract]) OR (Infestation[Title/Abstract]) OR (Infectious[Title/Abstract])))) AND ((Sports[MeSH]) OR ((((((((Sport[Title/Abstract]) OR (Athletic[Title/Abstract])) OR (Olympic[Title/Abstract])) OR (Paralympics[Title/Abstract])) OR ("World cup"[Title/Abstract])) OR (championship[Title/Abstract])) OR (marathon[Title/Abstract])) OR ("mass gatherings"[Title/Abstract])) OR (athletes[Title/Abstract]) OR (stadium[Title/Abstract]) OR ("sports venues"[Title/Abstract]) OR (gymnasium[Title/Abstract]))

**WOS:**

(TI=(Sports or Sport or Athletic or Olympic or Paralympics or "World cup" or championship or marathon or "mass gatherings" or athletes or stadium or "sports venues" or gymnasium) OR AB=(Sports or Sport or Athletic or Olympic or Paralympics or "World cup" or championship or marathon or "mass gatherings" or athletes or stadium or "sports venues" or gymnasium)) AND (TI=(Infections or Infection or Infestation or Infectious) OR AB= (Infections or Infection or Infestation or Infectious) OR TI=("Pathogen Transmission" or "Transmission, Pathogen" or "Transmission, Infectious Disease" or "Infectious Disease Transmission" or "Communicable Disease Transmission" or "Infection Transmission" or "Transmission, Infection" or "Transmission of Infectious Disease" or "Horizontal Transmission of Infection" or "Infection Horizontal Transmission" or "Community Transmission" or "Community Transmissions" or "Community Spread" or "Person-to-Person Transmission" or "Person to Person Transmission" or "Transmission, Person-to-Person" or "Autochthonous Transmission" or "Transmission, Autochthonous" or "Close-Contact Transmission" or "Close Contact Transmission" or "Transmission, Close-Contact") OR AB=("Pathogen Transmission" or "Transmission, Pathogen" or "Transmission, Infectious Disease" or "Infectious Disease Transmission" or "Communicable Disease Transmission" or "Infection Transmission" or "Transmission, Infection" or "Transmission of Infectious Disease" or "Horizontal Transmission of Infection" or "Infection Horizontal Transmission" or "Community Transmission" or "Community Transmissions" or "Community Spread" or "Person-to-Person Transmission" or "Person to Person Transmission" or "Transmission, Person-to-Person" or "Autochthonous Transmission" or "Transmission, Autochthonous" or "Close-Contact Transmission" or "Close Contact Transmission" or "Transmission, Close-Contact"))

**Embase:**

((Sports or Sport or Athletic or Olympic or Paralympics or "World cup" or championship or marathon or "mass gatherings" or athletes or stadium or "sports venues" or gymnasium):ti OR (Sports or Sport or Athletic or Olympic or Paralympics or "World cup" or championship or marathon or "mass gatherings" or athletes or stadium or "sports venues" or gymnasium):ab) AND ( (Infections or Infection or Infestation or Infectious):ti OR (Infections or Infection or Infestation or Infectious):ab OR ("Pathogen Transmission" or "Transmission, Pathogen" or "Transmission, Infectious Disease" or "Infectious Disease Transmission" or "Communicable Disease Transmission" or "Infection Transmission" or "Transmission, Infection" or "Transmission of Infectious Disease" or "Horizontal Transmission of Infection" or "Infection Horizontal Transmission" or "Community Transmission" or "Community Transmissions" or "Community Spread" or "Person-to-Person Transmission" or "Person to Person Transmission" or "Transmission, Person-to-Person" or "Autochthonous Transmission" or "Transmission, Autochthonous" or "Close-Contact Transmission" or "Close Contact Transmission" or "Transmission, Close-Contact"):ti OR ("Pathogen Transmission" or "Transmission, Pathogen" or "Transmission, Infectious Disease" or "Infectious Disease Transmission" or "Communicable Disease Transmission" or "Infection Transmission" or "Transmission, Infection" or "Transmission of Infectious Disease" or "Horizontal Transmission of Infection" or "Infection Horizontal Transmission" or "Community Transmission" or "Community Transmissions" or "Community Spread" or "Person-to-Person Transmission" or "Person to Person Transmission" or "Transmission, Person-to-Person" or "Autochthonous Transmission" or "Transmission, Autochthonous" or "Close-Contact Transmission" or "Close Contact Transmission" or "Transmission, Close-Contact"):ab)

**Scopus:**

TITLE-ABS-KEY ( sports OR sport OR athletic OR olympic OR paralympics OR "World cup" OR championship OR marathon OR "mass gatherings" OR athletes OR stadium OR "sports venues" OR gymnasium ) AND ( TITLE-ABS-KEY ( infections OR infection OR infestation OR infectious ) OR TITLE-ABS-KEY ( "Pathogen Transmission" OR "Transmission, Pathogen" OR "Transmission, Infectious Disease" OR "Infectious Disease Transmission" OR "Communicable Disease Transmission" OR "Infection Transmission" OR "Transmission, Infection" OR "Transmission of Infectious Disease" OR "Horizontal Transmission of Infection" OR "Infection Horizontal Transmission" OR "Community Transmission" OR "Community Transmissions" OR "Community Spread" OR "Person-to-Person Transmission" OR "Person to Person Transmission" OR "Transmission, Person-to-Person" OR "Autochthonous Transmission" OR "Transmission, Autochthonous" OR "Close-Contact Transmission" OR "Close Contact Transmission" OR "Transmission, Close-Contact" ) )

**Table S1. Risks, epidemics, and prevention measures of infectious diseases of each study.**

| **Held Year** | **Host Country** | **Sports Name** | **Disease** | **Main Transmission Route** | **Epidemics** | **Risks** | **Prevention** | **Surveillance** | **Record Number** |
| --- | --- | --- | --- | --- | --- | --- | --- | --- | --- |
| 1978 | United Kingdom | The 1978-79 Rugby Competition Season United Kingdom | Herpes | Blood-borne | A total of 48 players were affected, of whom 47 were forwards and 1 scrum-half. | The "gentleman's agreement" which prevents athletes from playing if they have skin trouble tends to be forgotten before important fixtures. | Achieve laboratory diagnosis in order to initiate appropriate therapy as soon as possible. | Questionnaires were sent to secretaries in 70 rugby union clubs, asking for details of any cases of skin disease. | 1 |
| 1987 | Australia | The 1987 Australian Orienteering Championships Australia | Molluscum | Blood-borne | / | 1. Minor lacerations 2. Contact with infected plants or by communal bathing after the competition. | Wear protective clothing and by the avoidance of communal bathing. | / | 2 |
| 1991 | United States | The 1991 International gymnastics competition United States | Measles | Respiratory tract | 3 suspected measles cases among athletes from New Zealand (NZ) participated in an international gymnastics competition. | / | 1. Prompt case confirmation and aggressive vaccination of many contacts within 72 hours of most intense exposure. 2. Promote cooperation and coordination among the competition's officials, the Health Department, and the laboratory and immunization personnel. 3. Guarantee the availability of jet injectors and multidose vaccine vials. 4. All people related to the sports event should be appropriately vaccinated according to the recommendations before arrival. 5. Delegation members, staff, and volunteers should be required to provide vaccination records or other documentation of immunity (such as serology or physician diagnosis) for measles and other VPDs. 6. Vaccination records should be maintained in data bases that include birth date, home address (including country), and telephone number, and similar data collected on vaccination consent forms must be legible. 7. Participants aged less than 18 years should have authority to designate an accompanying adult to authorize medical intervention. 8. In the event of a VPD outbreak, local and state health departments should work quickly with organizing committees and governing bodies to establish plans for evaluation, treatment, exclusion, and prophylaxis and to ensure vaccination clinics are held promptly and conveniently. | Surveillance for secondary cases included: 1. twice-daily reports from delegations on whether any member had prodromal measles symptoms;  2. daily review of visits to the competition's medical station and observation of the venue for persons with measles symptoms;  3. letters to participants, volunteers, and staff advising them of the outbreak and control measures, signs and symptoms of measles, the need to seek health care, and the importance of notifying local public health officials if symptoms occurred; 4. daily telephone calls to all emergency rooms and urgent-care centers in Marion County during September 16-23; 5. notification of states whose residents attended the competition. | 3 |
| 2020 | Japan | The 2020 Tokyo Olympics Tokyo, Japan | COVID-19 | Respiratory tract | An outbreak of respiratory tract infections affecting 45% of athletes and 31% of staff members of the Finish team was reported. | Asymptomatic persons carrying MERS coronavirus or SARS-CoV2 have been demonstrated to be a potential source of contamination to the population. | / | / | 4 |
| 2016 | Brazil | The 2016 Rio Olympics  Rio, Brazil | Zika | Vector | / | 1. If visitors become infected, even asymptomatically, and return home to tropical countries having the right mix of Aedes aegypti mosquitoes and overladen slums, that could establish local viral transmission, and new outbreaks of microcephalic, braindamaged children disabled for life. 2. a few Olympic travellers introduce those newly evolved strains to those continents, riddled with weak health systems (weaker than Brazil), the outcome could be dreadful. 3. Even assuming the Olympics have very low odds of spreading Zika virus globally, that is only half the issue, because if it eventuates the outcome—new microcephaly outbreaks among children—will be major and devastating. | 1. The Olympics could be delayed a year or two, pending proof (now lacking) that herd immunity in Brazil is effectively reducing incidence of Zika virus infection. 2. The Olympics could be moved, like the 2003 Women’s World Cup was as a result of severe acute respiratory syndrome (SARS) in China. | / | 5 |
| 2016 | Brazil | The 2016 Rio Olympics  Rio, Brazil | Zika | Vector | / | Aedes mosquitos mainly bite around dawn and dusk. Although ZIKV transmission can occur year around, it is more likely to happen during the summer. | 1. Protective clothing, mosquito repellants, avoiding visit to high-risk areas, air-conditioning and protected accommodations are essential preventative measures. 2. Take proper measures in the design and preparation of the official uniforms for athletes, team members, staff and volunteers. 3. Avoiding stretchy and Spandex fabrics and dark colors in uniforms and casual clothing can reduce the risk of mosquito bites. Clothing containing repellant agents can also provide extra protection.  4. A few number of countries, such as South Korea, have gone one more step by designing “Zika-proof” uniforms that include long pants, longsleeved shirts and jackets that contain mosquito-repellant chemicals.We suggest a routine practice of wearing long-pants and long-sleeved shirts by everyone during the games | / | 6 |
| 2014 | Brazil | The 2014 FIFA World Cup Brazil | Dengue | Vector | / | / | / | Calculated the risk of dengue for foreign visitors to the World Cup on the basis of past daily (not monthly) incidence in the 12 cities that will host the games. | 7 |
| 2010 | South Africa | The 2010 FIFA World Cup South Africa | Rift Valley fever, measles, HIV/AIDS,tuberculosis, H1N1, influenza, H3N2 | Respiratory tract/Blood-borne | / | 1. Mass gatherings have the potential to increase transmission of imported and endemic communicable diseases, including pandemic H1N1 influenza. 2. The biggest risk factor for tuberculosis is HIV and if people were worried about getting tuberculosis they should check their HIV status. | 1. With mass vaccination campaigns in Europe and the USA, most football fans travelling to South Africa from the northern hemisphere in June will already have had the jab, or have been sick and developed immunity before they get here. 2. Distributing 240 000 soccer balls with tuberculosis and HIV health messages to children countrywide. Taxis are also being targeted to carry public health messages. 3. The NICD and WHO has recommended that visitors to South Africa, especially those intending to visit farms or game reserves, avoid coming into contact with animal tissues or blood, avoid drinking unpasteurised or uncooked milk, and avoid eating raw meat. 4. Travellers should take precautions against mosquito bites with the use of insecticide-treated bednets and insect repellents. 5. If visitors are planning to visit game reserves, such as the Kruger Park, precautions should be taken. | / | 8 |
| 2008 | Switzerland, Austria | The 2008 European Football Championship Switzerland, Austria | Measles | Respiratory tract | The outbreak in Switzerland began already in November 2006 and 2,944 cases were reported until the start of the Euro 2008 (unpublished data) while the one in Austria started in March 2008 and 332 cases were reported before the event (unpublished data). | The measles outbreaks ongoing in both organising countries could have a potential for international spread. | All the participants agree that the daily exchange of information, the coordination epidemic intelligence and response activities at EU level and the close collaboration of the organising countries with ECDC has strengthened the established network of experts from different sectors nationally and internationally, which will function beyond the Euro 2008. | To better monitor the measles situation, Italy, Germany, Austria and Switzerland provided ECDC with weekly updates on the number of measles cases two weeks prior and after the Euro 2008. Weekly updating of ECDC about the measles situation from Austria and Switzerland is still ongoing. | 9 |
| 2016 | Brazil | The 2016 Rio Olympics  Rio, Brazil | Multi-infectious disease | Vector/Respiratory tract/Gastrointestinal/Blood-borne | / | / | 1. It is advisable that all travelers ensure that their routine immunizations are upto-date. Hepatitis A vaccine is very important. Hepatitis B vaccination is advised for those who could be at risk. 2. Travelers to Rio de Janeiro are at significant risk of travelers’ diarrhea. Therefore, all travelers are advised to ensure strict food and water hygiene.  3. Travelers need to be advised about the significant risks associated with unprotected casual sexual relations. | / | 10 |
| 2016 | Brazil | The 2016 Rio Olympics  Rio, Brazil | Zika | Vector | / | / | 1. Prevention of mosquito bites information dissemination education with the ultimate goal of minimizing anxiety for athletes and staff, a postexposure Zika screening strategy was implemented for asymptomatic but concerned team members.  2. Semen polymerase chain reaction (PCR) (males) and blood PCR (female) for Zika virus immediately on return from Brazil, in combination with 2 sequential Zika virus serological evaluations (IgM/IgG) at least 2 weeks apart was proposed. 3. Ultimately, the postexposure evaluation included a single serological evaluation for Zika virus (IgM/IgG) at the 2 to 3 week period postreturning to NZ from Brazil. This testing was only available on request after a comprehensive medical consultation during the Games period. | / | 11 |
| 2020 | United States | National Basketball Association (NBA) National Hockey League (NHL) National Football League (NFL) United States | COVID-19 | Respiratory tract | / | / | 1. Very similar to the recommendations put forth by the NCAA and the WHO, it is expected for players to social distance as much as possible; however, the rules in Orlando are strict as people will be wearing a device that sets off an audible alarm if they are within 6 feet of other people for more than 5 seconds; players and personnel are also likely to be wearing monitors to assess body temperature and blood oxygen levels. 2. Creating an isolated bubble will certainly lower the risk of COVID-19 spread, and as of now, the NBA appears to have the resources to provide players with all testing and monitors that they require. The approach taken by the NBA appears to be the most stringent requiring all games to be played in Orlando and all players to remain in the bubble for the duration of the season. 3. Players and personnel will undergo daily testing with results provided within 24 hours while in the hub cities; the NHL has also designated Secure Zones which include rinks, hotels, bars, restaurants, and entertainment options. 4. Testing of all players before arriving to camp and testing every day for two weeks or testing until positive cases are below 5% league-wide. 5. Along with testing, the league recommends to continue social distancing, wearing masks, and maintaining healthy hygiene habits. | / | 12 |
| 2018 | Korea | The 2018 Winter Olympics PyeongChang, Korea | Multi-infectious disease | Respiratory tract/Gastrointestinal | 1. A total of 288 patients were encountered in the emergency department (ED): 113 had injuries and 175 had illnesses. The patients comprised 180 men and 108 women.  2.The respiratory system was the most commonly involved system for all categories: staff members accounted for 45 patients and athletes accounted for 26 patients. The gastrointestinal tract was the second most commonly affected system for all categories: 28 cases occurred in staff and seven cases occurred in athletes. 3.The most common symptom that patients complained of was pain, with 49 cases reported in staff members, 24 in athletes, and 14 and 11 in the other and unknown categories, respectively. The second most common symptoms were respiratory symptoms (72 cases), including dyspnea, cough, and sputum. 4.Symptoms associated with the gastrointestinal tract, including diarrhea, vomiting, and dyspepsia, were the next most common symptoms (37 cases) (Table 1). For all categories, the most common cause of illness was infection (119 cases), followed by illness caused by environmental effects (35 cases) 5.The total number of transports to hospitals was 67, split between GMC (for MRI) (47 cases), GAH (16 cases), and Wonju Severance Christian Hospital (WSCH)(4 cases). Influenza tests were issued in 38 cases, among which seven patients tested positive. A norovirus rapid test and polymerase chain reaction (PCR) was performed in 17 cases, among which four patients (all athletes) tested positive. | Cold weather and a sudden change in surroundings might influence these organ systems, resulting in a greater number of cases involving these systems. | 1. The head of infection control and two nurses at Wonju Severance Christian Hospital(WSCH) rushed to the polyclinic and took charge of infection control in the polyclinic. One nurse from Pyeongchang Public Health Center and three staﬀ members from the Korea Centers for Disease Control and Prevention (KCDCP) also visited the polyclinic.  2. Second, suspected patients were carefully controlled through the treatment process, and the guidelines for treating patients with suspected norovirus were distributed. Suspected patients were isolated at their accommodations rather than instructing them to visit the polyclinic.  3. For medical services in the 2010 Vancouver Olympic Winter Games, the training program for medical staﬀ was a four-phase education and training curriculum, designed to foster the performance of a high-functioning interdisciplinary medical team. In our case, several meetings with the national Olympic Organizing Committee were held to improve the quality of medical services. In addition, test events were held, during which comprehensive medical services were provided. However, it would have been preferable to have a polyclinic operating simulation before opening the Olympic Games to provide familiarity with the working conditions and reduce errors. | Te polyclinic ED operated from January 25 to February 27, 2018. All cases were enrolled in this study, and their data were analyzed by date and category. | 13 |
| 2016 | Brazil | The 2016 Rio Olympics  Rio, Brazil | Zika | Vector | / | 1. ZIKV is mainly transmitted via a bite from a female mosquito of the Aedes species. 2. ZIKV also has been detected in semen (ZIKV remains present in semen longer than it does in blood), and sexual transmission from an infected male partner has been reported (7). 3. Furthermore, maternal-fetal transmission can occur vertically as well as during childbirth. Because it is a bloodborne disease, 4. ZIKV also can be transmitted through blood transfusions. 5. Another special concern is the introduction of infection to the mosquito population in countries of the Northern Hemisphere by athletes, athlete support personnel, and visitors returning from Brazil.  6. Although the Olympic Games will be held in August during the cooler and drier climate of Brazil’s winter, reducing Brazil’s mosquito density, spectators will return to the Northern Hemisphere’s late summer where the mosquito population will be at its peak, increasing the risk that uninfected mosquitoes will feed on infected travelers and then transmit disease. Because of this, returning travelers are advised to continue mosquito preventive efforts for 3 wk to ensure that ZIKV is not transmitted to uninfected mosquitoes. | 1. Minimizing or eliminating standing or stagnant water will significantly reduce areas for mosquito breeding and will reduce the mosquito vector load. Because the A. Aegypti mosquito flies low with a preference for biting humans during daytime and prefers to live indoors, it is recommended that athletes and spectators use approved mosquito repellents and cover exposed skin by wearing closed shoes, long loose pants, long-sleeved shirts, and hats thick enough to block a mosquito bite during the daytime. In sports with limited body cover such as wrestling and aquatic sports, particularly in open water swimming, use of appropriate mosquito repellents on all exposed skin is essential.  2. Pregnant women who have traveled to affected regions should be considered for serologic testing 2 to 12 wk after their return regardless of absence of symptoms. 3. Women returning from these areas are advised to avoid pregnancy for at least 8 wk even if fully asymptomatic. Men who have traveled to an area with known ZIKV are advised to use condoms for 8 wk to protect their sexual partners. 4. Set a plan for regular inspection of venues and water quality during the games. Furthermore, the United States Olympic Committee has organized an Infectious Disease Advisory Group to identify best practices that will prepare and protect athletes and staff attending the games. | / | 14 |
| 2020 | Japan | The 2020 Tokyo Olympics Tokyo, Japan | COVID-19 | Respiratory tract | / | / | / | A stochastic transmission model with the cooperation of seasonal factors which influence much on the transmission cycle of dengue virus, and carried out stochastic simulations for each of the scenarios provided adequately. | 15 |
| 2012 | British | The 2012 London Olympics London, British | Sexually Transmitted Infections | Blood-borne | / | / | / | We undertook an interrupted time-series analysis of surveillance data from the Genitourinary Medicine Clinic Activity Dataset (GUMCAD) collected at 33 genitourinary medicine (GUM) clinics in London and Weymouth (where Games events were concentrated) between 2009 and 2012. Mixed-effects linear regression models of weekly attendance and diagnoses, incorporating temporal trends, bank holidays, categorical month and clinic closures, were used to test for the effect of the ‘Olympic–Paralympic’ period. | 16 |
| 2019 | Greece | The 2019 Panhellenic Handball Championship Northern Greece | Clostridium perfringens | Gastrointestinal | / | Univariable analysis showed a statistically significant association of disease occurrence with the consumption of minced beef (RR:8.29 (95%CI 1,31-52,7)) served at the restaurant the teams visited on 26 June for lunch. All ill responders reported eating pasta with minced beef meat. Among the people that preferred pasta with grated cheese (without meat), none reported gastro symptoms. | Laboratory investigation for toxins should be enhanced, especially in foodborne outbreaks where clinical manifestations of the cases are found to be compatible with infection caused by a toxin. | A retrospective cohort study among the members of the four teams was performed. The local public health authority visited the restaurants where common meals took place, amassed information on the preparation of meals, and collected samples of leftovers. Stool samples were tested for Salmonella spp. and Shigella spp. | 17 |
| 2018 | Korea | The 2018 Winter Olympics PyeongChang, Korea | Influenza | Respiratory tract | 1. 20 out of 44 (45%) athletes and 22 out of 68 (32%) staff members experienced symptoms of the common cold during a median stay of 21 days. Eleven (26%) samples tested virus-positive using POCT. All subjects with inﬂuenza (n=6) and 32 close contacts were treated with oseltamivir. The aetiology of the common cold was fnally detected in 75% of the athletes and 68 % of the staff members.  2. Seven virus clusters were identifed. They were caused by coronaviruses 229E, NL63 and OC43, inﬂuenza B virus, respiratory syncytial virus A, rhinovirus and human metapneumovirus. The virus infections spread readily within the team, most commonly within the same sport discipline. 3. In agreement with our observations, 48% of 44 Norwegian cross-country skiers self-reported becoming ill during or 10 days immediately after the Tour de Ski. | Long-haul air travel is a wellknown risk factor for the transmission of contagious diseases. One person with influenza can transmit the infection to 5–10 people during an intercontinental flight. | Molecular POCT diagnostics proved clinically valuable. The aetiology of the common cold was identified in the majority of cases. The viruses were readily transmitted within the team. | We performed a prospective observational study of the common cold in Team Finland during the 2018 Winter Olympic Games. The chief physician recorded the symptoms of the common cold daily on a standardised form. Two nasal swabs were taken at the onset of symptoms. One swab was analysed within 45 min using a molecular POCT for respiratory syncytial virus and inﬂuenza A and B viruses. After the Games, the other swab was tested for 16 possible causative respiratory viruses using PCR in laboratory-based testing. | 18 |
| 2016 | Norway | The 2016 Youth Winter Olympics  Lillehammer, Norway | Multi-infectious disease | Vector/Respiratory tract/Gastrointestinal/Blood-borne/Water-contact | 1..Illness risk was highest in curling (17.2% of participating curling athletes) and in the Nordic skiing disciplines with biathlon (14.6%), nordic combined (14.3%) and cross country skiers (12.5%) suffering from at least one illness case during the games. Most of the illnesses were respiratory tract infections (n=66, 81.5%). Also, five cases of gastrointestinal infections were reported, corresponding to 6.2% of all reported illnesses (table 5). Almost two-thirds of all illnesses (26, 32%) prevented the athlete to train or compete for at least 1 day, in most cases due to respiratory track infections with fever, cough and pain symptoms for 1–2 days. 2.The high incidence of upper respiratory infections mirrors data from other Olympic, Paralympic and single elite sport events. The rate of illness in the Lillehammer was similar to those reported in the Innsbruck YOG9 (7% of all athletes affected in Lillehammer, compared with 8% of athletes in Sochi and in Innsbruck vs 7% in Vancouver). Also consistent with recent reports from multisport events with a 75% higher odds, female athletes were exposed to higher risk as their male counterparts. | Especially among endurance athletes extensively exposed to cold air or ice arenas. | More education on hygiene may be warranted through different channels to create awareness.60 63 64 A recent IOC consensus statement on load in sport and risk of illness provided a list of general guidelines for illness prevention in athletes. | 1. Both the IOC and several International Sports Federations have instituted comprehensive injury and illness surveillance systems longitudinally or in their main events to detect risk factors and mechanisms. 2. As in any surveillance study, a high response rate does not necessarily equal high data validity. The surveillance form was kept simple with only eight categories to complete for each case to sufficiently capture information for injury and illness surveillance. Still, we cannot rule out misclassification of injuries and illnesses, as of overuse injuries versus pre-existing injuries versus recurrences. 3. Only 36% of the injuries and 20% of the illnesses were captured by the NOCs and the Lillehammer 2016 staff, allowing us to cross-check data. The majority of minor severity injuries and illnesses was likely seen internally by the NOC medical staff only. These findings demonstrate how recording data among NOC medical staff and in the organising committee’s medical stations is vital to the scientific quality of the surveillance study. | 19 |
| 2020 | Japan | The 2020 Tokyo Olympics Tokyo, Japan | Dengue | Vector | / | / | 1. First, cyclical training or a certification program on tropical disease management is warranted for physicians, especially those working in non-infectious disease-designated hospitals or clinics.  2. Second, multi-language communication methods need to be strengthened especially in the health and hospitality sector.  3. Third, owners of accommodations should consider incorporating a formal tropical disease-training program for their staff members and have a contingency plan for infectious disease-suspected travelers. | Failure mode and effects analysis (FMEA) | 20 |
| 2014 | Russia | The 2014 Olympic Winter Games Sochi, Russia | Multi-infectious disease | Respiratory tract/Gastrointestinal/Blood-borne | A total of 159 illnesses (64%) affected the respiratory system, and these were most frequently reported in skeleton (21% of the athletes), cross-country skiing (10%), curling (8%), biathlon (8%), bobsleigh (8%), short track (7%) and Nordic combined (7%). The second, third, fourth and fifth most frequently affected systems were the digestive system (n=28, 11%),nervous system (n=13, 5%), skin and subcutaneous tissue (n=12, 5%) and genitourinary system (n=9, 4%), respectively. | Predominant risk factors are mechanical and dehydration stresses generated within the airways and the level of airborne pollutants, irritants and allergens inhaled by the athlete under high ventilatory exercise conditions. | The continuously accumulating evidence that injury and illness rates vary substantially between sports demonstrates the need for tailoring preventive measures to the specific context of each sport. | We employed the IOC injury and illness surveillance system for multi-sport events in this prospective cohort study. We asked all National Olympic Committee (NOC) medical teams to report the daily occurrence (or non-occurrence) of injuries and illnesses on a standardised medical report form. | 21 |
| 2018 | Russia | The 2018 FIFA World Cup Russia | Multi-infectious disease | Vector/Respiratory tract/Gastrointestinal/Blood-borne | A review of outbreaks at large sport events including the Summer and Winter Olympics, FIFA world cup and the EURO football cup from 1984 through 2015 found little evidence for infection outbreaks with the exception of 36 cases of influenza among participants to the Winter Olympiad in Salt Lake City in 2002 and a small outbreak of gastrointestinal disease affecting a single team during the pre-race sailing competition in Brazil in 2015, prior to the 2016 Olympics. More recently, the Pyeong Ghang 2018 Winter Olympiad may have been have been hindered by a norovirus outbreak days before the events commenced. This outbreak affected mainly security staff for the games rather than athletes or visitors. | / | Advising football fans should re-iterate the need for them to be up to date with the routine vaccination courses recommended in their home country. | / | 22 |
| 2016 | Brazil | The 2016 Rio Olympics  Rio, Brazil | Zika | Vector | / | 1. The majority of athletes who have been known to have competed in the Olympics while pregnant were in the first trimester, a time when the risks associated with maternal-fetal transmission are potentially most significant.  2. Exacerbating the risk of Zika virus in Olympic athletes is the potential for non–vector-borne transmission through sexual intercourse . 3. Despite the prevalence of barrier contraception, unintended pregnancy is a concern and would be at risk for Zika-related complications. Development of acute symptoms as a result of sexual transmission of the Zika virus is possible and could affect an athlete’s ability to compete. | 1. The mainstay for the prevention of a Zika infection is to avoid mosquito bites. The mosquitos that spread Zika mostly bite during the day, but precautions should still occur at night. 2. Athletes and other team members should avoid potential mosquito breeding sites when possible. Mosquito larvae breed in standing water, so it is prudent to avoid standing water that could be encountered in flower pots, tires, buckets, bottles, jars, and other similar containers. Athletes who train and compete in venues outside of the urban areas, such as marathon runners and cyclists, have increased risk of exposure to mosquitos. 3. These athletes must take extra care to follow recommendations for insect repellant and clothing treatment. 4. Wear long-sleeved shirts and long pants. 5. Stay in places with air conditioning or that use window and door screens to keep mosquitoes outside. 6. Sleep under a mosquito bed net. 7. Use Environmental Protection Agency (EPA)–registered insect repellents, which have been proven safe and effective, even for pregnant and breast-feeding women. 8. Treat clothing and gear with permethrin or advise others to purchase permethrin-treated items since a mosquito can bite through thin clothing. | / | 23 |
| 2019-2020 | Japan | The 2019 Rugby World Cup and the 2020 Olympics Japan | Multi-infectious disease | Respiratory tract/Gastrointestinal/Blood-borne | Few outbreaks associated with sports-based MGs have been reported in literature. Most were reported from the United States of America with one from the United Kingdom of Great Britain and Northern Ireland, limiting generalization. Their findings nevertheless imply important considerations: outbreak risk at sports-based MGs is low but not null; outbreaks occur among athletes and nonathletes, associated and unassociated persons and populations of high and low vaccination coverage; importation can spark an outbreak even in low-incidence countries; and, as noted in one article, the difficulties of conducting surveillance on international visitors could mean misunderstanding the size or nature of an outbreak or missing an outbreak entirely. | / | 1. Ministries of health, organizations, health-care providers and travellers should ensure up-to-date vaccinations of travellers before they attend MGs, and they should also promote and support travellers carrying updated vaccination records to assist the home country with any potential case or outbreak investigations. 2. As we have outlined, up-to-date vaccinations with additional preventive measures should be included in pretravel advice for visitors to the 2019 Rugby World Cup and 2020 Tokyo Summer Olympic and Paralympic Games, specifically for mumps, measles, rubella, influenza and IMD for all travellers and for hepatitis A and JE for travellers at higher risk.  3. When providing advice, health professionals should also inform travellers about the role they could play in transmitting or preventing the transmission of disease to MG attendees from across the world. | To assist ministries of health and related organizations in developing pre-travel advice, we summarized national surveillance data in Japan (2000–2016, to the extent available) for rubella, invasive pneumococcal disease, measles, non-A and non-E viral hepatitis, hepatitis A, invasive Haemophilus influenzae disease, tetanus, typhoid fever, invasive meningococcal disease, Japanese encephalitis, influenza, varicella, mumps and pertussis by calculating descriptive statistics of reported cases and reviewing trends. | 24 |
| 2009-2014 | United States | 2009-2010 Through 2013-2014 National Collegiate Athletic Association United States | Skin Infection | Blood-borne | During the 2009–2010 through 2013–2014 academic years, Athletic trainers (ATs) participating in the Injury Surveillance Program (ISP) reported 112 skin infections contracted by 87 unique student-athletes across 78 720 AEs. The resulting skin-infection rate was 14.23/ 10 000 AEs (95% CI = 11.59, 16.86; Table 1). Twenty student-athletes (23.0%) contracted multiple skin infections: 16 sustained 2 skin infections, 3 sustained 3 skin infections, and 1 sustained 4 skin infections. Of the skin infections identified, 22.3% (n=25) were recurrent. The majority of skin infections occurred during the regular season (n=76; 67.9%) and were identified during practice (n = 100; 89.3%; Table 1). Bacterial skin infections and fungal skin infections comprised 25.9% (n ¼ 29) and 21.4% (n ¼ 24), respectively, of the total skin infections. The rate for viral skin infections (6.35/10 000 AEs) was larger than those for bacterial skin infections (3.68/10 000 AEs; RR ¼ 1.72; 95% CI ¼ 1.09, 2.72) and fungal skin infections (3.05/10 000 AEs; RR ¼ 2.08; 95% CI ¼ 1.28, 3.39). The most commonly reported bacterial skin infection was impetigo (n ¼ 12; 10.7%), and the most commonly reported fungal skin infection was tinea versicolor (n ¼ 15; 13.4%). Impetigo constituted the largest proportion of recurrent cases (41.7% of all impetigo cases), followed by ringworm (33.3% of all ringworm cases) and herpes simplex I (37.0% of all herpes simplex I cases) | / | According to the National Athletic Trainers’ Association position statement on skin diseases, infection-control policies are recommended for all collegiate institutions, including cleaning and disinfecting of all facilities, encouraging hand hygiene, and ensuring the practice of athlete hygiene. Currently, the National Wrestling Coaches Association offers a Webinar on skin infections and the National Federation of State High School Associations emphasizes proper care for and prevention. Most skin infections were reported during practices. Interventions that reduce such clusters will help to dramatically reduce the rate of skin infections among collegiate wrestlers. This may include daily mat cleaning with bleach water, skin checks by ATs that exceed competition requirements, promotion of self skin checks, proper student-athlete hygiene, and other educational initiatives. | A convenience sample of 17 NCAA varsity wrestling teams provided 35 team-seasons of data; a team-season is defined as 1 season of 1 team. | 25 |
| 2016 | Brazil | The 2016 Rio Olympics  Rio, Brazil | Zika | Vector | / | / | 1. Several recommendations are available for reducing risk of acquiring ZIKV. Measures to avoid mosquito bites include wearing long sleeved shirts, use of insect repellant and staying in screened or air conditioned accommodations.  2. US-CDC and ECDC recommend that any travelers who are pregnant (any stage/ trimester) or planning to become pregnant, should avoid travelling to areas with ZIKV outbreaks. 3. If travel cannot be avoided or postponed, or if people live in areas where ZIKV transmission is known to occur, scrupulous measures to avoid mosquito bites during both daytime and nighttime hours must be taken.  4. Pregnant women in Zika-affected areas should wear protective clothing, apply a U.S. Environmental Protection Agency (EPA)-approved insect repellent, and sleep in a screened room or under a mosquito net.  5. Aedes spp mosquitoes predominantly bite during the day, especially around dawn and dusk, and thus the correct timing and use of mosquito repellents and other personal protection measures are key to preventing any vector-borne infection. 6. All travelers to the 2016 Olympic games should seek travel health advice from their family doctors or a travel medicine providers, well in advance of their travel. They should also consult the National Travel Health Network and Centre, the Latin American Society for Travel Medicine (SLAMVI) guidelines or their relevant local public health agency for up to date country information on requirements for pre-travel vaccinations (eg Yellow Fever) and malaria prophylaxis. Travelers should also be educated on safe eating and drinking habits such as choosing bottled water over tap water, making sure meat and seafood are cooked fully, and avoiding raw unpeeled fruits and vegetables, and wearing appropriate footwear and avoiding skin contact with sand to prevent worm infections, such as cutaneous larva migrans. Sex education for prevention of sexually transmitted diseases and the use of condoms is also important. | / | 26 |
| 2012 | British | The 2012 London Olympics London, British | Sexually Transmitted Infections | Blood-borne | Olympic visitors constituted 1% of new clinic attendances and were less likely to be diagnosed as having a new STI (adjusted odds ratio, 0.69; 95% confidence interval, 0.48–0.98; P = 0.040). | / | In this first multisite study to examine the effect of Olympic visitors on local sexual health services, the 2012 Olympic Games was found to have minimal impact. This suggests that a “business as usual” approach would have been sufficient. | Self-administered questionnaires (completed by 29,292 patients) were used to determine the visitor status of patients attending 20 STI clinics, between July 20, 2012, and September 16, 2012, in the host cities, London and Weymouth. Using routine surveillance data from the Genitourinary Medicine Clinic Activity Dataset version 2, Olympic visitors were compared with usual attendees (local residents and non-Olympic visitors) in terms of their demographic characteristics, services utilized, and STIs diagnosed using univariate and multivariate methods. | 27 |
| 2012 | British | The 2012 London Olympics London, British | Multi-infectious disease | Vector/Respiratory tract/Gastrointestinal/Blood-borne/Water-contact | 1. None of these outbreaks was reported from an Olympic venue, highlighting the tendency for all events in an Olympic City during the Games to be labelled as Olympics-related. 2. In the Beijing 2008 Olympic Games, the number of cases of communicable diseases (including gastrointestinal infections) paradoxically reduced by 40% compared with the previous year, and no infectious disease outbreaks were reported. 3. During the global pandemic in 2009 averted outbreaks of influenza A H1N1 at the Hajj and the Asian Youth Games, Singapore. 4. In the lead-up to the Games (June 2–27, 2012) several reports were received by the HPA of routine infections such as norovirus in several athletic teams (infections occurred before the teams arrived at the Athletes’ Village), and of chickenpox among crew on a floating hotel for Games’ staff. | / | 1. Some vaccine-preventable diseases needed more sensitive consideration to balance the competing interests of prevention of spread in a confined community against the desire of elite athletes to not be treated invasively while training and performing. This need shows the importance of real-time information and rapid public health risk assessment that takes into account the context of the incident. 2. To access information from as many sources as possible—eg, through media communications systems—is also important. Information sometimes appeared very quickly in the public domain through social media outlets such as Twitter. These reports were quickly addressed with the enhanced surveillance and reporting systems to assess validity. | Traditional surveillance The UK has well established public health surveillance based on clinician, environmental, and laboratory reporting, augmented by syndromic reporting systems. Several enhancements to these systems were implemented, including the addition of data for attendance at Olympics venues to the core reporting requirement of notified cases, and a move from weekly to daily analysis and reports. The national Centre for Infectious Disease Surveillance and Control routinely collates reports of incidents, outbreaks, and adverse trends from across the UK; during the Games, in addition to undertaking this daily, they collated the enhanced systems we describe in this Series paper. Daily analyses of mortality data were also done, and a new system was introduced for sentinel intensive care units to report unexplained illness of probable infectious cause.  Syndromic surveillance Before the Games, the HPA had two well established syndromic surveillance systems. These systems were the HPA/NHS Direct Syndromic Surveillance System, which provides so-called pre-primary care data using call information from the health advice telephone service for a range of syndromes, and the HPA/QSurveillance National General Practitioner (GP) Surveillance System, one of the largest GP surveillance systems in Europe, which monitors weekly consultation data from a network of more than 3500 GP practices across the UK. Further GP surveillance was done with the long-term sentinel surveillance scheme coordinated by the Royal College of General Practitioner’s Research and Surveillance Centre.   Event-based surveillance During the 2012 Games a national event-based surveillance team was the hub for reporting of incidents and outbreaks of an infectious disease from across the UK that might substantially aff ect the Games, by their effect on venues, Olympic staff , athletes, or visitors, or by the public’s perception of the Games. The team enhanced established systems by reviewing and collating daily incident and response reports submitted by all local health-protection teams. The team also reviewed the national public health case-management system (HPZone) for incidents and diseases of special interest. Information from both these sources was collated, and a Games-specific risk assess ment made according to agreed criteria.37 Information about any notable events identified was routinely reported daily to the national coordination centre, or more frequently, if needed.  Polyclinic reporting For the first time syndromic surveillance reporting was undertaken at the Games polyclinic. This polyclinic, in the Athletes’ Village in the main Olympic–Paralympic park, was the principal point of access to medical servicesfor athletes and others. Medical facilities were also located in every sporting venue, as well as in one of the main hotels housing the Olympic–Paralympic family. Each time a medical service was used, the doctor, first aider, physiotherapist, dentist, or other health-care provider recorded details of the consultation and treatment using a medical encounter form. These forms provided an electronic record of the signs and symptoms of the presenting illness or injury. The London Organising Committee of the Olympic and Paralympic Games (LOCOG) wanted to gain some understanding of the incidence and pattern of infectious disease during the Games time—for the first time an additional section was included on the form to enable this. This section was obligatory for care providers to complete, and asked whether the encounter was fever, rash, diarrhoea or vomiting, respiratory symptoms, jaundice, meningitis or encephalitis, or none of these.  International epidemic intelligence The HPA worked with international partners—particularly the European Centre for Disease Prevention and Control (ECDC) and WHO—to set up enhanced international surveillance for the 2012 Games. The team undertook joint risk assessments of incidents identifi ed as relevant through an agreed set of criteria designed for the Games, using methods developed for this purpose.  Reporting of hazards The HPA’s Centre for Radiation, Chemical and Environmental Hazards provided a daily environmental hazards situation report based on the gathering and analysis of data for both chemical and radiological incidents, along with any necessary expert public health advice. This report also included a range of environmental quality indicators including air quality, temperature, ultraviolet radiation levels, and pollen levels, as well as information on risks from river and surface-water flooding.  Enhanced microbiology services Enhanced clinical, public health, and environmental microbiology laboratory capability and capacity are necessary to meet the increased demands of a mass gathering. As well as additional routine testing requirements, response teams need the ability to rapidly scale up the testing capability as part of the response to an controls (particularly of water in new or vacant buildings and facilities) were properly applied. Additional tests are often requested by event organisers—eg, for the 2012 Games additional tests were done on samples from marinas, hotels, training camps, and ships, as well as on samples from swimming pools, spa pools, water systems, food services, and mobile food vendors. | 28 |
| 2012 | British | The 2012 London Olympics London, British | Multi-infectious disease | Respiratory tract/Gastrointestinal | 1. A total of 310 illnesses (41%) affected the respiratory system, and these were most frequently observed in athletics, beach volleyball, football, swimming and water polo (3–5% of the athletes). In beach volleyball, 61% of the illnesses were reported to be respiratory infections. The second and third most affected systems were the gastrointestinal tract (n=123, 16%) and dermatology (n=83, 11%), respectively. | 1. The high incidence of respiratory infections mirrors data from other elite sport events. Predominant risk factors are mechanical and dehydration stresses generated within the airways and the level of airborne pollutants, irritants and allergens inhaled by the athlete under high ventilatory exercise conditions. It has earlier been reported that airway hyperresponsiveness/asthma is the most common chronic medical condition experienced by both summer and winter Olympic athletes. | / | 1. The injury and illness report form was identical to the one introduced in the XXI Olympic Winter Games in Vancouver in 2010.19 With respect to injuries, we recorded the following information: accreditation number, sport and event, whether the injury occurred during competition or training, date and time, body part, type, cause and estimated time lost from competition or training. Likewise, we recorded the following information for illnesses: accreditation number, sport and event, date, diagnosis, affected system, main symptom(s), cause and estimated time loss. | 29 |
| 2010 | South Africa | The 2010 FIFA World Cup South Africa | Multi-infectious disease | Respiratory tract/Gastrointestinal | Ninety-nine illnesses were reported in 89 players (12.1% of all players) and this was equivalent to 135 per 1000 players or 7.7 per 1000 player days (95% CI 6.2 to 9.2). The majority of illnesses affected either the respiratory (40; 40.4%) or the digestive (26; 26.3%) system. The most frequent diagnoses were acute upper respiratory tract infection (31; 31.3%) and gastroenteritis (21; 21.2%) followed by sleep disorders (10; 10.1%). | / | / | 1. The applied methodology combined the FIFA injury reporting1 and the International Olympic Committee injury and illness surveillance systems to allow comparison with previous studies. 2. The medical report form was almost identical to the form that was developed for the 2009 IAAF and FINA championships, as well as the 2010 Winter Olympic games.12–14 However, the form was slightly modified to: (1) collect football-specific information on cause of injury (the coding used was in accordance with previous FIFA competitions); (2) include codes for illness using the International Statistical Classification of Diseases and Related Health Problems, 10th revision system; and (3) include the duration of the daily training. | 30 |
| 2015 | Qatar | The 2015 24th Men’s Handball World Championship Qatar | Multi-infectious disease | Respiratory tract/Gastrointestinal | The respiratory tract (mainly upper) was the most commonly affected system (n=31, 73.8%), followed by the gastrointestinal tract (n=5, 11.9%) and dermatological problems (n=3, 7.1%). The main symptoms were pain, ache or soreness (n=25, 59.5%), fever, excess sweating or chills (n=6, 14.3%), and nausea, vomiting or diarrhoea (n=4, 9.5%). Infection was reported as the most frequent cause of illness (n=32, 76.2%). Other causes included nutritional, endocrine or metabolic disturbances (n=3, 7.1%), environmental conditions (n=2, 4.8%), and pre-existing disease (n=2, 4.8%). The most frequent diagnosis was tonsillitis (n=14, 33.3%) and common cold (n=10, 23.8%). | During major international sports tournaments, athletes are exposed to crowded venues and a compressed training and match schedule, as well as environmental conditions, which they may not be used to. | A systematic approach to illness prevention may help to reduce the number of respiratory problems. After implementing preventive measures, such as general guidelines on illness prevention, screening for airway problems, a vaccination programme, and specific measures to minimise the risk of infection. | The medical staff of participating teams (n=24) were requested to report all new injuries and illnesses during matches and/or training on a daily basis throughout the event (15 January to 1 February, 2015). | 31 |
| 2016 | Brazil | The 2016 Rio Olympics  Rio, Brazil | Multi-infectious disease | Respiratory tract/Vector | / | 1. The spread of severe acute respiratory syndrome coronavirus (SARS-CoV) from China to Hong Kong and further to Canada was not due to a mass gathering, but to infected individuals travelling late in the incubation period or just after the onset of symptoms.  2. The introduction of West Nile virus to North America was probably through wild birds crossing the Atlantic, and it could not have been predicted.  3. Lastly, the outbreak of Middle East respiratory syndrome coronavirus (MERS-CoV) in Korea was caused by a single traveller waiting in an overcrowded hospital emergency room in South Korea.  4. MERS was estimated to have a low epidemic potential, and it was pointed out in this Journal that the outbreak was identified as being caused by MERS-CoV because it happened in a country with the resources (knowledge and laboratory facilities) to rapidly identify the virus. | 1. Vaccines are an important preventive tool for mass gatherings and should include the basic coverage provided by childhood immunization programmes, supplemented where appropriate with protection against meningitis and influenza, and yellow fever for mass gathering participants coming from yellow fever endemic countries. 2. A meta-analysis on the use of face masks and the reduction in risk of upper respiratory infections found a modest effect. Compliance is always higher during a study than in the real-life situation, and making face masks mandatory at mass gatherings is not presently recommended. | / | 32 |
| 2006 | Germany | The 2006 FIFA World Cup Germany | Varicella/Mumps/Norovirus/Salmonella | Respiratory tract/Gastrointestinal | 1. 61 cases of gastroenteritis were epidemiologically linked to the norovirus outbreak in Munich. By the end of the second week of June 2006, the outbreak had come to an end. 2. No case of measles associated with the World Cup was observed during the enhanced World Cup surveillance. | 1.Absolute increase in population and increase in population density 2.Population movement: exposure to "foreign" diseases | ‘Public health surveillance should be implemented at mass gatherings to facilitate rapid detection of outbreaks and other health-related events and enable public health teams to respond with timely control measures’. | 1. The enhanced surveillance system for the World Cup consisted of four major branches: acceleration of data transmission; introduction of an additional free-text reporting system; monitoring media sources for epidemiological events; Strengthening communication and interaction between the different public health stakeholders. 2. Additional information which complemented daily transmission of notifiable data reached RKI in a timely manner. | 33 |
| 2012 | British | The 2012 London Olympics London, British | Multi-infectious disease | Vector/Respiratory tract/Gastrointestinal/Blood-borne/Water-contact | / | 1. Contaminated food, water and the environment (e.g. mass production of food and a ‘just-in-time’ food chain distribution system) have the potential to cause explosive and extensive outbreaks of gastrointestinal diseases. 2. Unusual mixing patterns and high density of individuals during mass gatherings creates opportunities for efficient seeding of respiratory infections from single infected hosts.  3. Influenza, particularly because of its short incubation period, has the propensity to spread and cause extensive morbidity and mortality, and has frequently been noted at sporting and music events. 4. Recently Panton–Valentine leucocidin (PVL) positive Staphylococcus aureus (methicillin sensitive or resistant) causing severe skin and soft tissue infections has been reported in close contact sports participants in, e.g. wrestling, American football, rugby and judo. Risk factors include compromised skin integrity, skin-to-skin contact, and sharing of contaminated items such as towels. | 1. Front-line HPA clinical as well as HPA food, water and environmental microbiology laboratories have participated in real-time laboratory exercises using public health scenarios and simulated samples containing live organisms. Such scenario-based exercises have led to improvements in the reporting systems, better understanding of risk assessment and communication requirements during the Games 2. Collation of information and risk assessment will be led by a senior and experienced clinical microbiologist to coordinate the regional, reference and specialist microbiology activities in the event of an outbreak or incident.  3. The Olympics project management team established a data management group within the planning process whose activities were focused on: (1) mechanisms for effective collation and integration of laboratory, clinical and other datasets for both ‘routine’ Olympic outputs and in the event of significant infectious disease; (2) workforce planning – identifying the cadre of staff with data manipulation and information management skills and developing rotas for support. 4. The data management/informatics planning group has developed and implemented task and document management systems which allow effective cascade of tasks fit for audit trails and ensuring information governance. 5. Freshwater sports carry a unique threat of leptospirosis in participants Legionellosis may present as outbreaks during mass gatherings and prevention requires specific input from environmental engineers and inspectors who carry out regular inspection of water systems in hotels, hostels and other forms of accommodation. | 1. The dissipation of rumours is another important function of a world-class public health surveillance system designed for mass gatherings. Communication systems provide accurate laboratory results which inform public health risk assessment and assurance to the public through mass media. 2. As mentioned above, the HPA has strengthened its event-based surveillance capabilities, including enhancement of existing systems and setting up of new systems. Laboratory reporting of infectious diseases is one of the key components of surveillance. Reports generated through NHS and HPA frontline laboratories will feed into the national surveillance system of the HPA, through which they will inform an ongoing risk assessment and situation report. 3. Microbiological test results from key regional hospitals will be extracted from a more comprehensive respiratory virology surveillance system operating from key laboratories established during the 2009 influenza pandemic and analysed daily (DataMart). | 34 |
| 2010 | Canada | The 2010 Winter Olympics Vancouver, Canada | Listeria/Norovirus/Salmonella/Vibrio parahaemolyticus/Borrelia burgdorferi | Gastrointestinal/Vector | / | / | 1. It is recommended that all travelers receive a tetanusediphtheria vaccine if they have not done so within the last 10 years. It is also recommended that all children be up-to-date on routine immunizations, that all travelers be vaccinated for the measles, mumps, and rubella (two doses if born after 1956), and that all travelers receive an influenza vaccine. 2. To counter the effects of jet lag, suggests melatonin taken at bedtime (local time) on the first evening after arrival and continued for the next 5 days. However, instead of melatonin, the World Health Organization suggests the use of short-acting sedatives. There is no perfect solution to jet lag but it is recommended that travelers limit alcohol intake and exercise during the flight by stretching and walking the aisle of the airplane. 3. It is strongly recommended that Olympic visitors avoid any sexual contact with prostitutes and drug users while in Vancouver. | / | 35 |
| 2007 | West Indies | The 2007 International Cricket World Cup West Indies | gastroenteritis, fever and respiratory symptoms | Gastrointestinal/Respiratory tract | / | / | / | 1. Back-up mechanism for data transfer (ie fax or phone) is essential. Realtime information sharing was also important and the use of a simple listserv was invaluable in this regard. Early planning should be done, taking into account the length of procurement processes for supplies and allowing sufficient time for training and piloting of the system prior to the event. It was advantageous to build the Mass gathering surveillance system (MGSS) on the already existing surveillance system. Flexing an already familiar system resulted in a smoother transition into and out of the MGSS.  2. regional and international collaboration was very important to the successful execution of the surveillance activities. | 36 |
| 2006 | Germany | The 2006 FIFA World Cup Germany | Measles/Hepatitis A/Legionella/Norovirus/Salmonella/Tick-borne encephalitis/Haemolytic uraemic syndrome/shigellosis/campylobacter/meningitis/hantavirus/listeriosis/rotavirus | Vector/Respiratory tract/Gastrointestinal/Blood-borne/Water-contact | 1. 71 FIFA 2006 World Cup-marked cases from eight events were reported to SurvNet, of which 63 were Norovirus cases related to the Munich broadcasting company outbreak. The remaining eight comprised four Salmonella and four Campylobacter cases (two of the latter being part of the same event). 2. There were several incidents where press reports identified an issue before it was notified to RKI through the enhanced surveillance system. These included a case of meningococcal disease in Bavaria (not FIFA 2006 World Cup-related); gastrointestinal illness in the Croatian national football team (event 2 June 2006, press report 3 June 2006, in RKI report 9 June 2006); and cases of chickenpox in a Togo national team player, and in an Indonesian journalist. | 1. Infectious disease transmission may be promoted by an increase in population density, importation of unusual pathogens, strains on infrastructure, and changes in services (such as food stalls) or behaviour (increased demand for sex workers). 2. Potential drivers for increased infectious disease transmission occur commonly, due to commuting, seasonal travel and smaller-scale gatherings such as concerts and conferences. | The surveillance evaluation suggests that satisfactory event surveillance can be achieved through minor temporary adaptations of an existing routine infectious disease surveillance system. Our approach enables good comparability with data from other areas and time periods, but also means that the extra efforts required for a mass gathering can be harnessed for longer-term improvements in routine surveillance. | 1. The 2 days of surveillance immediately before the match was designed to provide a short run-in period for the system, and the 2 days following the final match to allow capture of the commonest, short-incubation period gastrointestinal infections. 2. There were four main enhancements to infectious disease surveillance during the FIFA 2006 World Cup: accelerated transmission of notifiable disease case-data (from weekly to daily); marking cases of relevance to the FIFA 2006 World Cup in routine notifications (SurvNet); daily supplementary reports from all cities hosting matches; and the National Enhanced Surveillance Operations Centre (NESOC), staffed Monday to Saturday, in the national epidemiology centre (RKI) which collected, analysed and reported on daily surveillance inputs.  3. Communication with local and state health departments was facilitated by teleconferences, email and telephone communications, and pre-World Cup training sessions. A commercial text search for 14 infection-related keywords including ‘bacteria’, ‘epidemic’ and ‘infection ’, of 25 German newspapers was used to screen for relevant press reports. | 37 |
| 2005 | Palau | The 2005 VII South Pacific Mini Games  Palau | upper respiratory illness | Respiratory tract | There were no outbreaks of infectious disease detected during the games. | / | It is recommended that future events adopt a similar approach, with a strong emphasis given in the planning stages to establishing direct contact between the staff responsible for surveillance and field and visiting health personnel. | Surveillance for infectious diseases and injury was carried out by the Epidemiology/Intelligence team (Epi Team) established for the event. Health providers traveling with visiting teams or working in Koror were requested to complete daily log sheets of encounters using standardized case definitions. These sheets were collected each evening, either from designated “drop-off” points or directly from the team accommodation, and entered into a Microsoft Access database. Reports were generated and reviewed each morning to provide current statistics for the Incident Command meeting and determine further actions as appropriate. | 38 |
| 2011 | Germany | The 2011 FIFA Women’s World Cup Germany | Not specified | Not specified | / | / | 1. Based on experiences during the Men’s World Cup in Germany, Schenkel et al. had stated that “introducing an additional, sensitive, non-case definitionbased written report system was overall beneficial” and recommended “additional reporting systems that are flexible and not bound to case-definitions, provided that at least one case-definition system or syndrome-based system is in place” 2. Involving the participating stakeholders early-on in the planning phase for tailoring an event-specific enhanced surveillance system secures ownership and later participation in the actual surveillance measures. Conducting a needs assessment is thereby a helpful tool to establish communication lines and to exchange ideas in the preparation phase. | 1. Pre-event collection and distribution of stakeholders’ contact details 2. Daily (Monday to Friday) transmission of infectious disease notifications from district via state level to the RKI 3. Using the tag "World Cup 2011" for World Cupassociated infectious disease cases in daily transmission 4. Twice-per-week reports of unusual events (including null reporting) from district health authorities to the RKI via the corresponding state health authority 5. Twice-per-week report (Monday and Thursday) of the RKI to district and state health authorities and ministry of health including – feedback report of daily district infectious disease reporting – feedback report of “reports of unusual events” from districts and state health authorities – summary report of World Cup relevant national/ international epidemiological events 6. Phone conferences among stakeholders (on demand) | 39 |
| 2011 | France | The 2011 Paris European Athletics Indoor Championships Paris, France | Multi-infectious disease | Respiratory tract/Gastrointestinal | 1. During the 2009 and 2011 International Association of Athletics Federations (IAAF) World Championships, time-loss illness incidence was 23.2 (Alonso et al., 2010) and 12.4 (Alonso et al., 2012) illnesses per 1000 registered athletes, respectively. The respiratory tract was the most commonly affected system (36–39%).  2. About three-quarters of illnesses affected the upper respiratory tract (n = 13; 72%). Gastrointestinal illnesses (n = 3; 17%), dermatological problems (n = 1; 6%) and ophthalmologic problems (n = 1; 6%) comprised the other illnesses. The most commonly reported symptom was dyspnea/coughing (n = 5; 28%) and rhinorrhea (n = 5; 28%), followed by diarrhea/vomiting (n = 3; 17%), pain (n = 2; 11%), and fever (n = 1; 6%; symptoms missing: 2). No episodes of cardiovascular related collapses or syncope were recorded.  3. The most commonly reported cause of illness was infection (n = 10; 56%), followed by allergies (n = 3; 17%), and environmental (n = 3; 17%; unknown causes: 2). Upper respiratory tract infection was the most commonly reported diagnosis (n = 8; 44%), followed by upper respiratory tract allergy (n = 3; 17%) and gastroenteritis (n = 3; 17%). Three illnesses (17%; two gastroenteritis and one flu) were expected to result in time loss from sport, equivalent to an incidence of 4.8 illnesses per 1000 registered athletes (95% CI: 0.0–10.1). | During indoor championships, the temperature difference between the stadium and outdoors could be an extrinsic illness risk factor in addition to the athlete intrinsic predispositions. About a quarter of upper respiratory tract affections were caused by allergies that could be explained by the dusty and dry environment in the arena. However, no upper respiratory tract infections or allergies resulted in time loss from sport. Thus, this common illness was not severe during the event studied. | / | 1. National team physicians were asked to report all newly incurred injuries and illnesses daily on a standardized injury report form.  2. In addition, injuries and illnesses were reported by the local organizing committee (LOC) for the medical centers in indoor area and hotels. In case of duplicate reporting, information from the national team physician was preferred to the LOC physician’s report. 3. And, other tools should be used to improve the completeness of data (retrospective interview of athletes, media monitoring or web-based system; Bjorneboe et al., 2011. | 40 |
| 2012 | British | The 2012 London Olympics London, British | food- and waterborne and zoonoses, vaccine-preventable diseases, emerging and vector-borne diseases, tuberculosis, airborne diseases, human immunodeficiency virus (HIV) and other sexually transmitted infections and antimicrobial resistance and healthcare-associated infections | Vector/Respiratory tract/Gastrointestinal/Blood-borne/Water-contact | / | / | / | 1. Information regarding infectious and non-infectious events is collected from a database for event-based surveillance named the Threat Tracking Tool. 2. A team composed of three ECDC experts (generic expert team) was assigned to evaluate potential public health threats to the Games, as well as to select and prioritise SIDEs for the event-based surveillance system. To this end, the generic expert team opted for a multilevel approach including the selection of infectious diseases for prioritisation, qualitative scoring of diseases using a consensus-building Delphi method and a risk matrix. | 41 |
| 2010 | Canada | The 2010 Winter Olympics Vancouver, Canada | Respiratory diseases (eg, measles, varicella, influenza).  Enteric and food-borne diseases (eg, norovirus, salmonella, hepatitis A)  Sexually transmitted infections (eg, hepatitis B, human immunodeficiency virus, chlamydia, syphilis, gonorrhea).  Vector-borne diseases (eg, dengue fever, malaria)  Diseases spread by close contact (eg, meningococcemia, methicillin-resistant Staphylococcus aureus) | Vector/Respiratory tract/Gastrointestinal/Blood-borne/Water-contact | / | 1. The geographic location of the event 2. Nature of the infrastructure provided, including housing and food services 3. Characteristics of the local population 4. Characteristics of those attending the event 5. Athlete-specific factors, including any underlying medical conditions. | 1. Developing detailed food safety plans with Games’ caterers to reduce the risk of food-borne illness. 2. Provide seasonal influenza and pandemic H1N1 vaccines to Games staff and volunteers. 3. For those diseases that are vaccine preventable, immunization is the most important prevention tool.  4. Sexually transmitted infections have longer incubation periods than some other communicable diseases and therefore may not manifest until an athlete returns home from a sporting event. It is important for sport medicine physicians to advise all athletes to bring a supply of condoms and to use them for all sexual contact. More than 100 000 condoms were distributed in Vancouver and Whistler during the 2010 Winter Games and were widely available in the Athletes’ Villages. | / | 42 |
| 2012 | British | The 2012 London Olympics London, British | Multi-infectious disease | Vector/Respiratory tract/Gastrointestinal/Blood-borne/Water-contact | / | / | / | Health Protection Event-Based Surveillance (EBS) Traditional event-based surveillance is generally recommended as an addition to the basic systems of indicator-based surveillance in order to fill potential gaps and to detect cases or outbreaks that did not enter the basic surveillance net or were not detected in it. | 43 |
| 2016 | Brazil | The 2016 Rio Olympics  Rio, Brazil | Multi-infectious disease | Vector/Respiratory tract/Gastrointestinal/Blood-borne/Water-contact | / | / | 1. On the basis of our results, mosquito bite prevention, food and water precautions and avoidance of skin contact with soil should be recommended for travellers to Brazil.  2. Vaccination against influenza should be considered for those in risk groups. Vaccination against illnesses such as yellow fever and malaria prevention should be considered, based on individual itineraries in Brazil as detailed in the ECDC health risk assessment | EuroTravNet (a GeoSentinel subnetwork) | 44 |
| 2016 | Brazil | The 2016 Rio Olympics  Rio, Brazil | Measles | Respiratory tract | / | 1. Most of these cases were associated to foreign travellers that came to the USA from Europe, demonstrating how infected travellers can contribute for triggering an outbreak in susceptible areas.  2. Cases of measles transmission in domestic flight have also been reported in the literature demonstrating that the virus may be easily transmitted in confined places to susceptible population. 3. Considering all previous data, the Olympic Games present a favourable setting for measles transmission for susceptible population due to mass gatherings with people shedding the virus in places that are often confined, like restaurants, airports, public transports, malls and also areas of competition. | 1. Require vaccination of all tourism professionals and staff groups that will work directly and indirectly with the event, which includes airship crews, hotel staff and restaurants employees, securities and health professionals. 2. Orientation of medical community for fast diagnosis of disease is also important to avoid the rapid spread of the virus like the one occurred in northeastern region in 2014. 3. In addition, Brazilian travellers should seek for information regarding disease transmissions risks during a trip. | / | 45 |
| 1998 | Japan | The 18th Oita International Wheelchair Marathon Oita, Japan | Upper respiratory tract infection (URTI) | Respiratory tract | The study included 21 persons with spinal cord injuries (SCI). All the subjects were males. The incidence of URTI after the race was not high compared to control subjects who did not participate in the race. | The greater incidence of symptoms of infection is attributed to possible drying of the mucosal surfaces resulting from hyperventilation of cold, dry air may be applicable to disabled persons during the wheelchair marathon training period. | / | Examined the self-reported infectious episodes of upper respiratory tract infection (URTI) in athletes with spinal cord injuries (SCI) during a 1-month period before the race and 2 weeks after the race. | 46 |
| 2014 | Brazil | The 2014 World Cup & The 2016 Olympics Brazil | Multi-infectious disease | Vector/Respiratory tract/Gastrointestinal/Blood-borne/Water-contact | / | 1. Infestations involving exposed skin and acquired outdoors, often on or near beaches. These are found in many tropical areas but have been especially common in travelers to Brazil. 2. Visitors also pose risks to the host country. Visitors could carry pathogens that could spark a local epidemic, if the local population is susceptible or local conditions favor spread. 3. Although Brazil has largely controlled transmission of Chagas disease directly by the rejuviid bug, multiple outbreaks have occurred where apparently locally prepared sugar cane juice, acai juice, or other foods have become contaminated with trypanosomes via crushed bugs or their excreta, and this represents a potential risk for travelers to some areas | 1. This is a reminder of the importance of sexual contacts among travelers and the need to reinforce messages about avoiding unsafe sex and activities that would allow exposure to blood and body fluids (such as shared needles, injections, and tattoos). 2. Events should receive influenza vaccine. Because measles has caused outbreaks during and after mass events in the past, it is prudent for all travelers to be immune to measles through immunization or previous infection. 3. Knowledge of incubation periods is useful when evaluating returned ill travelers. In the pre-travel consultation, clinicians should assess specific locations within Brazil that the traveler plans to visit and the traveler’s potential activities in order to determine their need for yellow fever vaccination, malaria chemoprophylaxis, other travel vaccinations, and to identify and discuss other possible risks. | / | 47 |
| 2013 | Japan | The 2013 Sports Festival Tokyo, Japan | Multi-infectious disease | Respiratory tract/Gastrointestinal | / | / | / | 1. This was conducted by utilizing the surveillance systems that were already active on a daily basis, with enhancements on the information sharing between parties that normally conduct surveillances independently, making daily reports, conducting risk assessments on a daily basis, and structuring a response framework when additional action is deemed necessary. 2. Four surveillance systems, namely the Official Syndromic Surveillance (OSS), Ambulance Transfer Syndromic Surveillance (ATSS) (12), Pharmacy Surveillance (PS) (13,14), and (Nursery) School Absenteeism Surveillance system ((N)SASSy), all of which operate routinely, were utilized. | 48 |
| 2008 | Switzerland | The 2008 European Football Championship Austria, Switzerland | Methicillin-resistant Staphylococcus aureus (MRSA) | Blood-borne | In general, the two countries with the lowest amount of yellow and red cards per play time had the lowest proportions of MRSA. | 1. National MRSA proportions increase with more unfair play of the national teams. 2. Demographic and socioeconomic factors, as well as safety culture, are known to influence the MRSA situation. | 1. Adherence to MRSA guideline. 2. Hand hygiene compliance. | 1. We obtained methicillin resistance data for S. aureus from the European Antimicrobial Resistance Surveillance System (EARSS) project in 2008, which surveyed the antimicrobial susceptibility among invasive (from blood and cerebrospinal fluid) S. aureus isolates. 2. All teams which qualified for the European Football Championship, 2008 and had reported data to the EARSS were included in the analysis, i.e. Czech Republic, France, Germany, Greece, Italy, Netherlands, Poland, Portugal, Romania, Spain and Sweden. | 49 |
| 2012 | British | The 2012 London Olympics London, British | Gastrointestinal | Gastrointestinal | 1. There was no increase in the number of specimens received for culture, Cryptosporidium, OCP or norovirus tests during the Games period.  2. Community specimens: There was no difference in the number of specimens received during the Games period compared with outside the Games period. The odds of having a positive culture result for all specimens were lower during the Games period compared with outside the Games period. 3. Hospital specimens: There was no difference in the number of specimens received during the Games period. The odds of having a positive OCP result was higher during the Games period compared with outside the Games period (OR53.19, 95 % CI 1.58–6.43, P50.001), with higher odds for hospital specimens (OR59.83, 95 % CI52.20–43.86, P50.003). 4. HPU outbreak and incident data: The descriptive analysis suggests that there was no increase in the number of gastrointestinal outbreaks reported to the London HPUs during the Games period. | / | / | 1. In response to the risk assessment, existing surveillance systems were enhanced and new systems were developed to monitor infectious disease activity throughout the Games period. In addition to this, microbiology laboratory services were enhanced in order to meet any increase in demand. 2. The Health Protection Agency (HPA) Microbiology Service Division comprised eight Public Health Laboratories (PHLs), five Food, Water and Environmental (FWE) Laboratories, and two Reference Laboratories. 3. Enhanced reporting systems were also implemented to provide daily updates for public health situation reports. 4. Data were collected retrospectively for all public health, community and hospital stool specimens received by PHL London between 1 January 2010 and 31 December 2012. Cross-sectional time-series data analysis was used to ascertain whether there was a significant increase in the number of stool specimens received and/or the proportion of positive results during the Games period (2 July to 30 September 2012). Clinical reports of outbreaks to the four London HPUs were also analysed. | 50 |
| 2004 | Portugal | The 2004 European Football Championship Portugal | Foodborne disease/meningococcal/legionnaires | Respiratory tract/Gastrointestinal/Blood-borne | Visitors were not affected, furthermore, cases among residents seemed not to be influenced by the presence of thousands of visitors. | 1. Foodborne outbreaks Several risk factors were identified, such as contaminated raw food, inadequate storage and transport, poor premises hygiene; bad hygiene practiced preparing the meals and long time lags between preparation and consumption.  2. Legionnaires’ disease The only reported case of legionnaires’ disease occurred in a woman aged 38 who had been admitted to hospital, with two important risk factors: she was a smoker, and had an HIV infection. | The local health authority interventions included inspecting restaurants and imposing corrections, educating food handlers and treating two food handlers who tested positive for Staphylococcus aureus. | As well as hospital physicians, we considered other potential sources of notifications: the normal statutory reportable disease system, a public health laboratory for cases of foodborne outbreaks, the general public or any other source, provided we could validate the information. | 51 |
| 1991 | United States | The 1991 International Special Olympics (ISO) Games Minneapolis, United States | Measles | Respiratory tract | 1. 16 secondary measles cases were reported in athletes, spectators, and volunteers.  2. At least 9 cases of subsequent spread of measles occurred in three states as a resuIt of transmission from the 16 outbreak-associated cases. | 1. Measles was transmitted in a turbulent airflow through the ventilation system rather than via concentric dispersion of the virus throughout the stadium. 2. Transmission to participants likely occurred in three settings: track and field events, first aid stations, and the opening ceremonies. | 1. Improve community-wide measles immunization ability to withstand importation of disease with no or limited disease transmission. 2. Ensure adequate immunization of participants at international sporting events. Vaccination records should be maintained in data bases that include birth date, home address (including country), telephone number, and vaccination histories.  3. All state health departments, Special Olympics chapters, MDH(Minnesota Department of Health), international delegations, volunteers and spectators should be notified of the outbreak. | 1. Clinical, epidemiologic, and laboratory data were collected on all confirmed measles illnesses in participants and all suspected secondary and community-acquired measles illnesses related to the outbreak.  2. Cases were interviewed by local health officials to ascertain potential sources of exposure. | 52 |
| 2010 | South Africa | The 2010 FIFA World Cup South Africa | Rabies | Blood-borne | / | 1. There is widespread poverty and a lack of primary health care for animals. Even owned dogs may wander as packs searching for scraps of food.  2. Sustaining a mass vaccination campaign is an expensive exercise, and fnancial pressures due to other diseases outbreaks have been a major factor in hampering rabies control in KZN in recent years. | 1. Two ways of prophylaxis of rabies are recommended: post-exposure prophylaxis, i.e., vaccination after an exposure to a (potential or suspect) rabid animal has occurred, and pre-exposure prophylaxis. 2. Control measures, i.e., mainly dog vaccination campaigns in villages, associated with community awareness program related to the hazard of dog bites and the importance of post-exposure prophylaxis. | / | 53 |
| 2010 | Canada | The 2010 Winter Olympics Vancouver, Canada | Multi-infectious disease | Vector/Respiratory tract/Gastrointestinal/Blood-borne/Water-contact | / | / | The capability to integrate knowledge of worldwide patterns of commercial air travel and infectious disease surveillance in real time could significantly enhance global situational awareness of infectious disease threats. | 1. The Canadian-based Global Public Health Intelligence Network, permit real-time global situational awareness (by leveraging media reports on the Internet) that typically outperforms traditional surveillance efforts and that exists outside politically influenced communication channels. 2. By integrating knowledge of worldwide patterns of commercial air traffic with global surveillance of infectious diseases via Web-based intelligence-gathering tools. 3. HealthMap is an online resource that monitors a wide range of information sources for reports and warnings of outbreaks of infectious disease at the local or regional level An automated text-processing system evaluates more than 30 000 sources for disease outbreaks and their associated locations and maps the information to an interactive display (see www.healthmap.org). To accomplish this dual objective, we took advantage of the widespread availability of RSS (really simple syndication) feeds to construct an automatically updating data set of news stories about infectious diseases. | 54 |
| 2009 | Singapore | The 2009 Asian Youth Games Singapore | H1N1 | Respiratory tract | / | / | 1. These control measures included temperature screening at the airport, the isolation of H1N1 cases in hospital and quarantine of their close contacts. Delegates who were unwell were advised not to travel to Singapore, and vaccination against seasonal flu was encouraged to reduce ‘noise’ caused by nonpandemic flu during the games period. Communication to the general public through press releases and media conferences was also needed to manage public concern that the games were bringing visitors from affected countries even as locals were advised against unnecessary travel to such areas. 2. Containment measures involved the early detection and confirmation of influenza A (H1N1) cases through frequent temperature checks as well as close monitoring for flu-like symptoms among participants. Confirmed cases were then isolated (with antiviral treatment) and close contacts quarantined (with antiviral prophylaxis). Health kits containing masks and thermometers were provided to all athletes and officials to facilitate at least twice daily temperature and health checks, which were ensured by the team officials, coaches and liaison officers.  3. All overseas participants with fever and/or acute respiratory symptoms were encouraged to seek medical attention at the GVMC immediately to facilitate early assessment and diagnosis. Those fulfilling the criteria for H1N1 tests had their samples taken and sent to the Singapore General Hospital. Results from the PCR tests were usually available within 6–8 h, during which patients were isolated in holding rooms at the GVMC pending the results. Later on, immunofluorescence testing for influenza A subtype was introduced to provide results as quickly as within 4 h. Cases who were negative for influenza A based on immunofluorescence could then be released earlier from the holding rooms | The DORSCON is a five-colour (green, yellow, orange, red, black) grading system that is linked to a response plan that allows a risk management approach appropriate to the transmissability and virulence of the virus. | 55 |
| 2010 | South Africa | The 2010 FIFA World Cup South Africa | HIV | Blood-borne | / | / | 1. With the tournament captivating the entire region, the public health community should have better applied our knowledge and evidence-based interventions to raise the profile of HIV prevention, care and treatment.  2. More should have been done to prevent possible new infections fueled by visitors, alcohol and sports celebrations. | / | 56 |
| 2006 | Germany | The 2006 FIFA World Cup Germany | Measles | Respiratory tract | / | / | Advise travellers to Germany, especially football fans and people travelling to this state, to ensure that they have had measles vaccination before their trip. The mumps, measles and rubella (MMR) vaccine is recommended 1. Suspected measles is a notifiable event in Germany and such notification will trigger investigations and control measures as indicated by the local health authorities. 2. Affected and exposed persons will be given advice on how to reduce the risk of transmitting the infection to others. 3. The national reference laboratory for measles at the RKI offers free laboratory testing of suspected measles cases and has distributed non-invasive as well as traditional testing kits to the local health departments where World Cup games are taking place. 4. People from foreign countries who are suspected to be infected with or exposed to the measles (or other notifiable infectious pathogens) will also be asked to contact the local health authority at home once they have returned. A form will be filled out in English which will contain the individual’s self-reported travel itinerary, details on the diagnostic evidence and additional epidemiological information. 5. Information concerning important infectious disease events during the World Cup in Germany, updated daily, is available in English and German at http://www.rki.de. | / | 57 |
| 2006 | Germany | The 2006 FIFA World Cup Germany | Unspecified | Not specified | / | / | / | 1. The surveillance preparations are based on an integrated, state-administered, electronic database – SurvNet@RKI - which is already in place. Data collected at local health level are entered into various software systems (by multiple manufacturers). Once data have been electronically transmitted to state health departments, they are imported into SurvNet@RKI. The notification data is then uploaded from the state SurvNet@RKI systems into the federal SurvNet@RKI system, which contains data for the whole of Germany. Anonymised public health data from SurvNet@RKI are made available to the general public through the interactive, web-searchable SurvStat@RKI system, updated on a weekly basis.  2. An enhanced, notification-based disease surveillance will be implemented. This system will be based on the routine infectious disease surveillance already existing in Germany, which will be enhanced by the daily transmission of all mandatory notifications to RKI. Each of the 12 local health departments directly responsible for stadium locations will report to the RKI every morning, via a restricted-access electronic communication forum available to all local and state health departments. Reports will concern notifiable disease cases, confirmed and preliminary, as well as reports of suspected, unconfirmed, or unusual occurrences that have come to the attention of health departments via non-systematic means (such as outbreak investigations, media reports, etc). The 12 reports will be summarised and synthesised into a larger federal report released by RKI on the same day the local reports are received. In addition to national information, an international situation report will also be included, summarising the world situation with respect to infectious disease outbreaks, especially those linked to countries participating in the World Cup. Sources will include the World Health Organization (WHO), European Centre for Disease Prevention and Control (ECDC), European Early Warning and Surveillance System (EWRS), and several others. 3. The enhanced surveillance system will be tested over one week, one month before the games begin. During this time the daily reporting system and expedited transmission system will be conducted using current, actual data from that given week. This is intended to be a full simulation exercise for the World Cup period. The normal reporting mechanism will serve as a baseline, as the basic methodology of infectious diseases under surveillance is not being changed. 4. Syndrome-based surveillance has been undertaken at several previous mass gatherings . However, at the current time, it is not clear whether, in regions with a well-functioning surveillance system in place, a syndrome-based system provides more than minimal additional information that is not identifiable through routine surveillance. Evidence-based research on the effectiveness and cost-effectiveness of syndromic surveillance at mass gatherings is needed, especially given the high cost of implementation. After careful consideration in consultation with the local and state health departments, and in light of this uncertainty, it has been estimated that the enhanced mandatory notification surveillance system would be sufficient, and a syndrome-based surveillance system will not be implemented. | 58 |
| 2016 | Brazil | The 2016 Rio Olympics  Rio, Brazil | Zika | Vector | / | / | 1. Olympic officials have promised regular checks at Olympic venues and at athlete accommodation for any stagnant water where mosquitoes tend to breed.  2. It advised athletes and visitors to practise safe sex. 3. Choose air conditioned accommodation, use insect repellent, and wear light coloured clothing that covers as much of the body as possible. | / | 59 |
| 2010 | South Africa | The 2010 FIFA World Cup South Africa | HIV | Blood-borne | / | / | 1. The meeting recommended public health campaigns that target potential clients with education and condoms as well as the setting up ‘sex worker-safe’ clinics, a moratorium on sex workrelated arrests during the World Cup, and ongoing engagement of FIFA. | / | 60 |
| 2010 | South Africa | The 2010 FIFA World Cup South Africa | Cholera/Diphtheria/Hepatitis A/Hepatitis B/influenza/Meningococcus/measles/mump/rubella/polio/rabies/tuberculosis/tetanus/typhoid fever/yellow fever | Vector/Respiratory tract/Gastrointestinal/Blood-borne/Water-contact | / | / | 1. Vaccine. 2. If travellers are planning side trips to risky areas, they need to be advised to adhere to appropriate malaria chemoprophylaxis and careful personal protective measures. 3. List of some Internet resources for health and safety in South Africa. | / | 61 |
| 2016 | Brazil | The 2016 Rio Olympics  Rio, Brazil | Dengue | Vector | / | 1. Individuals travelling to new areas can bring pathogens with them, be exposed to infections they have not encountered previously, or return to their homes and infect others.  2. World Cup fans might be at risk for vector-borne diseases. Vectors have come and gone in Brazil. 3. Risk for dengue infection is determined by the likelihood of A aegypti bites and number of infected bites. 4. Travellers, particularly those attending matches in high-risk cities, identified as Recife, Fortaleza, and Natal by Lowe and colleagues, might return home with dengue. | 1. Informed World Cup fans will protect themselves from a aegypti through choice of screened or airconditioned accommodation, the use of insecticide, and appropriate light coloured and loose fitting clothing. 2. Doctors must be aware of causes for febrile illness in World Cup spectators. 3. Diagnosis of dengue is important for exclusion of other diagnoses, such as malaria. Symptomatic treatment will be sufficient for most patients with dengue, apart from the few individuals, if any, who develop severe dengue. | Rachel Lowe and colleagues present findings derived from spatiotemporal modelling to forecast dengue risk at a fi ne spatial scale. The investigators project risk for microregions in Brazil and calculate risk levels that are useful for planning dengue control in the 12 cities where matches will be played Their early warning system is based on a spatiotemporal Bayesian hierarchical model framework driven by climate and non-climate information. | 62 |
| / | United States | Major League Baseball United States | COVID-19 | Respiratory tract | / | / | 1. Certain MLB health and safety protocols, which include frequent diagnostic testing for rapid case identification, isolation of persons with positive test results, quarantine for close contacts, mask wearing, and social distancing, might have limited COVID-19 transmission between teams. 2. The health and safety protocols established tiered, risk-based testing for MLB teams, which called for persons who received a positive SARS-CoV-2 test result to be placed in isolation and for close contacts to be quarantined separately.  3. Selected mitigation strategies implemented by Major League Baseball at the opening of the 2020 season. 4. MLB isolated all players and staff members with COVID-19 at a separate location from those under quarantine. MLB coordinated with Philadelphia Department of Public Health to ensure rapid identification of cases and testing of contacts and to implement mitigation efforts. The lack of evidence for on-field transmission, as demonstrated by the absence of infections among opposing on-field team players and staff members, pointed to indoor exposures as the likely means of SARS-CoV-2 spread. | / | 63 |
| 2008 | China | The 2008 Olympics Beijing, China | HIV | Blood-borne | / | It would appear that internal migration of commercial sex workers may become an important vector for the transmission of HIV during the 2008 Olympic Games. | A greater focus on 100% condom usage needs to be encouraged should travelers engage in sexual activity with the local population, either on a casual or commercial basis. This advice also applies to homosexual travelers, as the prevalence of HIV and sex-related risk factors appears to be increasing among men who have sex with men in Beijing. | / | 64 |
| 2006 | Italy | The 2006 Winter Olympics Torino, Italy | influenza/meningitis/Streptococcus pneumoniae | Respiratory tract | 1. During the games, notifications of infectious diseases were in line with those reported in the same period during the previous year, except for a slight increase in incidence of varicella. 2. Compared with the same period in 2005, we registered a lower incidence of meningococcal meningitis and a higher incidence of meningitis due to Streptococcus pneumoniae. 3. The incidence of influenza-like illness during the games was in line with national data, and lower than that recorded during the same period in 2005. 4. Syndromes from the 13 groups were reported in 5282 people. The most frequently reported syndromes were respiratory syndrome with fever (2355, 45%) and gastroenteric syndrome (1831, 35%). A statistical alert was generated on 38 days (64%), but only one cluster of gastroenteritis was epidemiologically confirmed. | / | / | An integrated epidemiological surveillance and response system was set up for the 2006 Olympic Winter Games in Torino, the integrated surveillance system consisted of: 1. reinforcing existing resources and improving routine surveillance, in order to increase timeliness; 2. setting up new surveillance systems; 3. establishing contacts with regional and national authorities dedicated to Olympic Games safety; 4. setting up a dedicated website. 1. Statutory notifications of infectious diseases. This system was made more timely by requiring immediate notification of suspect cases of selected diseases and clusters to the local and regional health units.  2. Laboratory-based surveillance of invasive diseases. Complete information on every patient with a bacterial isolate from blood or cerebrospinal fluid was transmitted daily by 12 microbiology laboratories to SeREMI. 3. Sentinel surveillance of influenza-like illness. Twenty one general practitioners provided data to SeREMI on a daily. 4. Syndromic surveillance was set up specifically for the Olympic Games. Syndromic surveillance was implemented in the Olympic sites, and in the following health facilities in Torino province, where the games took place | 65 |
| 2000 | Australia | The 2000 Olympics Sydney, Australia | herpes/cocksackieviruses /echoviruses/meningococcal/measles/gastroenteritis/legionnaires/influenza/tuberculosis/pertussis/yellow fever | Blood-borne/Gastrointestinal/Water-contact/Respiratory tract/Vector | / | / | 1. In the event of a major public health incident, investigations will be elevated to the State level.  2. To assist any largescale investigations, the telephone call-room used by the NSW Health Survey Program will be on stand-by to conduct interviews or provide information to the public. 3. Also, public health investigation teams located in public health units on the periphery of metropolitan Sydney are on stand-by, to be deployed in the event of major public health incident.  4. In the event that an emergency is declared, the coordination and control arrangements for any investigations will come under the provisions of the NSW Healthplan. | 1. Routine surveillance of public health conditions in NSW is conducted through 17 Public Health Units in Area Health Services and a centralised Public Health Division within the NSW Health Department (NSW Health). Under the NSW Public Health Act (1991), medical practitioners, hospital chief executives (or general managers), pathology laboratories, directors of childcare centres and school principals are required to notify certain medical conditions to the local public health unit.  2. The NSW Health Olympic Surveillance System (OSS) will be used to monitor acute disease outbreaks and potentially preventable injuries. This system integrates multiple data sources. 3. Daily transfer: Notifiable Diseases Database (NDD), Emergency Department Olympic Surveillance System (EDOSS), National and global epidemic surveillance, Food safety monitoring, The vessel inspection program, Environmental inspection program. Weekly transfer: Influenza surveillance. | 66 |
| 2007 | West Indies | The 2007 International Cricket World Cup West Indies | malaria/dengue/chikungunya | Vector | / | The concern with dengue, which is present in the Caribbean, is the introduction of new serotypes. | Information booklets in English are delivered to all travellers from countries participating in the tournament and disembarking in the French départements. This booklet recaps the main methods for individuals to protect themselves against malaria, stresses the need to consult a doctor on contracting a fever and gives the relevant health authorities’ telephone numbers. | 1. As an increase in travel across the region is anticipated during the event, the Département international et tropical of France’s Institut de veille sanitaire (International and Tropical Department of the Health Surveillance Institute, DIT-InVS) has established an enhanced surveillance system for the period, together with the Cellule Inter Régionale d'Epidémiologie Antilles-Guyane (Inter-Regional Epidemiology Unit for Antilles-Guyana, CIRE) and international partners. 2. To support epidemic intelligence, heightened media monitoring in the region is also being undertaken by the Medical Information System (MedISys, http://medisys.jrc.it), a project run by Directorate General Health and Consumer Affairs of the European Commission (DG SANCO) that uses online electronic information sources to rapidly detect, track and assess threats so that advance warning can be provided. | 67 |
| 2009-2017 | Global | 11 international athletics championships between 2009 and 2017 | Multi-infectious disease | Vector/Respiratory tract/Gastrointestinal/Blood-borne/Water-contact | The most common illness location was the upper respiratory tract (30.0%), followed by the gastrointestinal tract (20.0%), the cardiovascular system (15.4%) and the neurological system (9.9%). | / | Educate athletes and their entourage on prevention of infectious diseases, dehydration and heat stress; implement simple prevention strategies, like proper hygiene measures, drinking regularly and only bottled water, eating only ‘safe’ food, regular hand washing with alcohol gel, surveillance of special indoor air cleaning systems, decrease contact with people outside the team, and be aware of changes in temperature and climatic conditions Screening tests for airway problems and suitable strategies for athletes with a heavy competition load should be offered to all athletes at risk. We conclude that illness prevention strategies during athletics championships should be tailored for endurance and explosive disciplines. The focus should be on upper respiratory tract infections, exercise-induced dehydration/fatigue/hypotension/collapse and gastroenteritis/diarrhoea. | During 11 international championships held between 2009 and 2017, physicians from both national medical teams and the local organising committees reported daily on all athlete illnesses using a standardised report form. Illness frequencies, incidence proportions (IPs) and rates (IRs), and relative risks (RR) with 95% CIs were calculated. | 68 |
| 2006 | Italy | The 2006 Winter Olympics Torino, Italy | Anthrax/Botulism/Cholera/Diphtheria/Viral hepatitis/ Viral hemorrhagic fever/Yellow fever/Relapsing fever/Legionellosis/Bacterial meningitis/Measles/Plague/Poloomyekitis/Rabies/Typhus exanthematicus/Cluster of foodborne disease/Trichinosis/Tularaemia/Smallpox/Tuberculosis | Vector/Respiratory tract/Gastrointestinal/Blood-borne/Water-contact | / | / | / | 1. The health authorities in the Piemonte Region, the Italian Ministry of Health (Centro per il Controllo delle Malattie, CCM) and the Istituto Superiore di Sanità have therefore implemented an integrated surveillance system, to be maintained during the Olympic and Paralympic Games, to detect any specific adverse health events early, and to promptly and effectively intervene and control them. 2. During the games, medical assistance will be provided both by the National Health System (NHS), and by an ad hoc system set up by the Olympic committee (TOROC). 3. Within the NHS, 15 hospitals have been selected as ‘Olympic hospitals’ to provide care during the games. The regional health service also has a permanent telephone line for emergencies, which is linked to services which can respond to situations requiring urgent interventions. In addition, a 24 hours/7 days active network of physicians for non-urgent health care has been set up. 4. The statutory notification system is a case-based national surveillance system which covers all infectious diseases which has been in place since 1991. In this system, physicians who diagnose or suspect an infectious disease are required to notify the case to the LHU, which is responsible for investigating the case and any required control measures. With the exception of selected diseases (for example, cholera, yellow fever, plague), as a rule, notifications are transmitted from LHU to the regional and national level on a monthly basis. However, during the winter games, physicians will immediately notify the LHU of any suspect cases of selected diseases, and this information will in turn be immediately forwarded to Seremi. For all other diseases, data transmission from LHU to Seremi will occur on a daily basis. 5. Laboratory-based surveillance of invasive diseases Since 2001, all 45 NHS microbiological laboratories in Piemonte have been recruited to participate in surveillance of invasive diseases. Twelve of these laboratories are located in the area involved in the games. Case-based information relative to each patient with a bacterial isolate from blood or cerebrospinal fluid will be daily transmitted to Seremi by these 12 laboratories. 6. Sentinel surveillance of influenza-like illness The sentinel surveillance of flu-like illness was implemented in Italy in 2000, and it is based on general practitioners and primary care paediatricians. In Piemonte, 50 physicians are participating in the network, and 22 are located in the area of the games. Under usual conditions, physicians transmit case numbers (aggregated by age group) on a weekly basis. During the winter games, the 22 physicians will transmit the same data daily to Seremi. 7. Syndromic surveillance Syndromic surveillance has been set up specifically for the Olympic Games. A list of 13 syndromes has been defined (Table 2), and instructions for coding were provided to physicians. Syndromic surveillance will be based on: 8. Toxic exposure surveillance Three reference poison control centres located in a contiguous region (Lombardia) will send all data to Seremi daily concerning consultations in Piemonte. These case-based data will include information on toxic exposures (for example, food, drugs, environmental) and clinical symptoms. 9. The European Centre for Disease Prevention and Control (ECDC) will also have access to the web community, and the ECDC will provide updated information about current international health threats. The ECDC will assist the national team if necessary (for international contact tracing, for example). Contact points in France have also been identified, in order to facilitate the exchange of information about transborder events. | 69 |
| 2012 | Britain | The 2012 Olympics London, Britain | Multi-infectious disease | Vector/Respiratory tract/Gastrointestinal | / | / | / | The ReSST coordinates four main surveillance systems; HPA/NHS Direct (a national telephone health advice line run by NHS Direct), HPA/QSurveillance [in-hours general practitioner (GP) consultations], GP Out-of-hours/unscheduled care (GPOOHSS, out-of-hours GP consultations) and the Emergency Departments Syndromic Surveillance System (EDSSS, emergency department attendances), the latter two having been developed as part of the enhanced Games surveillance programme. | 70 |
| 2009 | Martinique | The 2009 Race in the Tropical Forest Martinique | Leptospirosis | Water-contact | Ten (91%) of the 11 ill athletes who were tested were confirmed by PCR or serology. Serogroup Pyrogenes was commonly found. Cutaneous cuts, reported by 14 (73.7%), was the only potential risk factor using univariate analysis. | Cutaneous cuts, reported by 14 (73.7%), was the only potential risk factor using univariate analysis. | 1. Sporting event participants in tropical areas should be made aware of specific warnings and recommendations concerning the risk of leptospirosis, especially after periods of heavy rainfall or flooding.  2. Considering the higher risk of leptospirosis in tropical countries, especially after heavy rainfall or periods of flooding, travelers and participants to outdoor events with inevitable exposure to potentially contaminated water or soil should be informed as to personal prevention measures and chemoprophylaxis. 3. the first steps of prevention in athletes should be to avoid swimming in rivers, swallowing lake or river water, and prevent dermal cuts. | The list of all participants in the event, including their telephone numbers and e-mail addresses, was obtained from the race organizers. An information letter was sent to all participants, in which they were informed of their potential exposure to leptospirosis during the race. Participants who did not return their questionnaires were contacted by telephone. A wide environmental investigation was performed, comprising sampling lake water and various domestic and wild animal testing. | 71 |
| 1998 | United States | The 1998 Triathlons Wisconsin and Illinois, United States | Leptospirosis | Water-contact | Of the 1194 athletes surveyed, 110 (9%) who participated in one or both events described an illness meeting the case definition. Of the 110, a total of 73 (66%) sought medical care; 23 (32%) of those were hospitalized. | / | Issued a precautionary advisory not to swim, water ski, or use personal watercraft at Lake Springfield. | To identify cases of febrile illness, a standardized telephone survey was conducted of athletes who participated in the event. | 72 |
| 2017 | Netherlands | The 2017 Obstacle and Mud Run Participation Netherlands | Multi-infectious disease | Respiratory tract/Gastrointestinal/Blood-borne | The median age was 33 years (range: 5–71 years) and 1,435 (53%) were male. Following an obstacle run, 2.7% of respondents reported AGI, 3.7% reported SI and 5.8% reported RI. The majority of respondents reported that they had no allergies or other chronic diseases (83.1% and 84.5%, respectively). A small number of respondents (8.1%) used medication at the time of the run. In total, 641 of 2,813 (22.8%) respondents reported health complaints (e.g. headache, stomach ache and vomiting) following participation in an obstacle run. | The ingestion of mud was associated with AGI | 1. To avoid ingesting water/mud by trying to keep their mouths closed during obstacle runs. This advice arose due to similar findings regarding the risk for infectious diseases observed in other (outbreak) investigations related to events that include water or mud, e.g. mountain bike events and city swims. 2. Proper handwashing is a very effective measure for the prevention of infectious disease. In our study, we found that 13 obstacle runs did not have adequate handwashing facilities, e.g. with running water, soap and paper towels. Food was distributed at 14 obstacle runs and, as it is not practical for participants to wash their hands during an obstacle run (and they are likely covered in mud when the food is distributed), unpeeled fruits and packaged foods may be better options. 3. We recommend that organisers of obstacle runs inform participants of infectious disease risks and potential preventive measures they could take, e.g. practicing good hand hygiene, not participating if they are ill, not swallowing mud and showering directly after the run.  4. In addition, we recommend that organisers adequately facilitate these preventive measures, e.g. by installing proper handwashing and shower facilities and only distributing foods that are unpeeled/packaged during the obstacle run.  5. Based on visual inspections, we also recommend that organisers fulfil the national hygiene guidelines regarding the toilets and showers around the obstacle run course. | Between April and October 2017, we conducted a retrospective cohort study among 2,900 participants of 17 obstacle runs in the Netherlands. Demographic, symptomatic and behavioural data were collected from participants via an online questionnaire 1 week after participation in an obstacle run. Stool specimens were obtained from respondents for microbiological tests. | 73 |
| 2009 | Italy | The 2009 International Swimming Federation (FINA) World Championships Roma, Italy | Upper respiratory tract infection (URTI) | Respiratory tract | A total of 184 acute illnesses were reported, which is equivalent to 7.1% of the registered athletes suffering an illness during the Championships. About half of the illnesses affected the respiratory system (n=91; 50.3%) and a fifth the gastrointestinal system (n=36; 19.9%). The most commonly reported symptom was pain. The most frequent diagnosis affected the upper respiratory tract including ‘otitis’ (n=31; 16.8%) and ‘tonsillitis’ (n=18; 9.8%). | URI are more common in elite athletes than in non-competitive athletes. This is thought to be due to the increased risk of infection from such factors as over training induced ‘immunosuppression’ and from crowding at competition venues. This is supported by the data from Rome where the highest incidence of illness was reported in swimming, with the highest number of competitors exposed to more crowding in warm-up areas and event call rooms. | / | 1. The IOC Injury Surveillance system was implemented with the extension to also survey illness.  2. The team physicians or, in their absence, a team physiotherapist were asked to report daily on the occurrence (or non-occurrence) of all newly incurred injuries and illnesses. | 74 |
| 2012 | Ukraine | The 2012 UEFA EURO Poland, Ukraine | Measles | Respiratory tract | / | Health advice was not appropriately disseminated. Despite the efforts made by public health organisations to raise awareness regarding measles vaccination and the continuous media coverage, both before and during EURO 2012, messages went unnoticed by a significant number of fans attending the tournament. It may be that people travelling to or within Europe have a false sense of security when it comes to health. | 1. Social networks could serve as innovative platforms to conduct surveys, enabling rapid access to target populations at low cost and could be of use during upcoming mass gatherings such as the Olympics. 2. Pre-travel health advice issued by international organisations such as the World Health Organization (WHO) and the European Centre for Disease Prevention and Control (ECDC) as well as national health authorities recommended measles vaccination to all Euro 2012 visitors. Public health messages were released via a number of different platforms including leaflets, official public health websites, other websites and the media. | We conducted a cross-sectional study among EURO 2012 fans. We set up an anonymous, self-administered, internet-based survey on the Union of European Football Associations (UEFA)’s EURO 2012 Facebook profile. | 75 |
| 2010 | Austria | The 2010 Triathlon Langau, Austria | Leptospirosis | Water-contact | In July 2010, four cases of leptospirosis occurred in athletes after a triathlon held in Langau, a village in the province of Lower Austria, close to the Czech Republic. | 1. Heavy rains that preceded the triathlon are likely to have caused leptospiral contamination of the lake. Urine from small mammals (e.g. rodents like mice and rats), wild boars or domestic pigs are possible sources of Leptospira.  2. Persons can get leptospirosis by swimming or wading in fresh unchlorinated water contaminated with animal urine leptospirosis. | 1. Although doxycycline prophylaxis does not prevent leptospiral infection in an endemic area, it has a signifcant protective eﬀect in reducing the morbidity and mortality during outbreaks.  2. Although no active case fnding was performed in this Austrian outbreak, it would have been informative to know if any other participants experienced fever or illness, or if anyone was on doxycycline for prophylaxis via survey. | / | 76 |
| 2012 | Britain | The 2012 Olympics London, Britain | Not specified | Not specified | / | / | / | The Health Protection Agency (HPA) has set up a suite of robust and multisource surveillance systems. These include enhancements of already established systems (notification of infectious diseases, local and regional reporting, laboratory surveillance, mortality surveillance, international surveillance, and syndromic surveillance in primary care), as well as new systems created for the Games (syndromic surveillance in emergency departments and out-of-hours/unscheduled care, undiagnosed serious infectious illness surveillance). | 77 |
| 2002 | United States | The 2002 Winter Olympics Salt Lake City, United States | Influenza | Respiratory tract | A total of 2,635 medical visits were recorded during the Games; patients with any respiratory symptom represented 12%. Of these, 188 satisfied the symptom criteria for the study and were screened for influenza. Influenza A was detected in 28 (15%) and influenza B in 8 (4%) patients. Athletes comprised 36% of all influenza patients. | / | 1. The intervention strategy integrated a policy of empiric treatment based on clinical data and viral testing with a public health surveillance approach, including daily review of all viral test results from the Polyclinic and reports of influenza in the community. Potential clusters of influenza were promptly identified, index patients were treated with oseltamivir, and contacts were given oseltamivir prophylaxis. 2. A low rate of influenza immunization was noted among participants. The World Health Organization and others have suggested that vaccination is beneficial for athletes. We support issuing a public health alert that encourages administering influenza vaccine to all athletes and staff before a large international event is staged. | Viral test results from the Polyclinic and public health reports of influenza in the local community were reviewed daily. Patients with ILI or confirmed influenza were offered treatment with oseltamivir; close contacts were offered prophylaxis. | 78 |
| 2014 | Micronesian | The 2014 8th Micronesian Games Pohnpei, Federated States of Micronesian | Multi-infectious disease | Vector/Respiratory tract/Gastrointestinal/Blood-borne/Water-contact | Influenza-like illness (ILI) was the most common syndrome reported (55%, n = 225). Most syndrome cases (75%) were among people from Pohnpei. Only 30 cases out of a total of 408 syndrome cases (7%) presented with acute fever and rash, despite the large and ongoing measles outbreak at the time. No new infectious disease outbreak was recorded during the Games. Peaks in diarrhoeal and ILI cases were followed up and did not result in widespread transmission. | / | / | 1. Pohnpei implemented an early warning syndromic surveillance system in 2010. Data are collected daily from the central public hospital (Pohnpei State Hospital) and weekly from the private hospital (Genesis Hospital) for acute fever and rash, prolonged fever, influenza-like illness and diarrhoea syndromes covering important outbreak-prone diseases in Pohnpei State. A weekly surveillance report is disseminated to DHSA and WHO.  2. The introduction of the web-based tool greatly improved the timeliness of data entry, analysis and SitRep dissemination, providing assurance to the Games organizers that communicable diseases would not adversely impact the Games. | 79 |
| 2015 | Latvia | The 2015 Riga Cup Riga, Latvia | Salmonella Enteritidis | Gastrointestinal | 30 cases of gastrointestinal disease. | / | 1. The Finnish ice-hockey teams scheduled to play on the last tournament weekend were prior advised to take standard hand hygiene and food safety precautions, such as eating only cooked foods and drinking bottled beverages. 2. Standard hand hygiene and food safety precautions were recommended and participants were also advised to notify THL in case gastrointestinal symptoms appeared during or after the competition. 3. On 30 April and 8 May the Latvian public health authorities contacted the organisers of both events and recommended to consume only well-cooked foods and maintain proper hand hygiene. In addition, a phone number to a specialist from the Latvian public health authority was provided in case any of the event participants would develop gastroenteritis symptoms. | The investigation included a description of the outbreak, retrospective cohort study, microbiological investigation and trace-back. | 80 |
| 1998 | United States | The 1998 Triathlons Wisconsin and Illinois, United States | Leptospirosis | Water-contact | After the event, 120 triathletes sought medical care; of these, 75 had an acute febrile illness. Eventually, leptospirosis was confirmed in 52 of these patients by use of a variety of laboratory methods. | / | / | Laboratory surveillance. | 81 |
| 2016 | Brazil | The 2016 Rio Olympics  Rio, Brazil | Zika | Vector | The risk of getting Zika at the Olympics is relatively low. | / | But if people do a good job of being cautious and using insect repellents, I don’t think there’s any evidence that additional measures are particularly necessary or even useful. | / | 82 |
| 2016 | Brazil | The 2016 Rio Olympics  Rio, Brazil | Zika | Vector | / | / | 1. The WHO statement advised athletes and visitors to practise safe sex, choose air conditioned accommodation, use insect repellent, and wear light coloured clothing that covers as much of the body as possible. Travellers should avoid “impoverished” and “overcrowded” areas in the city, it added.  2. Pregnant women were warned not to travel and were advised to practise safe sex or abstain from sex with partners returning from Zika hit areas during their pregnancy. | / | 83 |
| 1984 | Sweden | The 1984 Cross-country runner Sweden | Molluscum | Blood-borne | This study found a 3% prevalence of molluscum contagiosum confined to the knee region in a group of healthy athletes. | 1. Direct skin contact with each other in the sauna, in the shower, or on the benches. Soaps, brushes, and towels may also spread the infection if used by many people.  2. Transmitted from one competitor to another during the competition by branches, bushes, or barbed wire.  3. Transmitted after the competition in connection with bathing and washing. | Improved protection for the entire length of the legs. All runners had worn the compulsory covering competition overall, often made of thin nylon, which usually was enforced over the shins with protectors or stockings with plastic enforcement. | 1354 competitiors at a randomly chosen orienteering competition answered a brief written inquiry whether they had or had had a skin rash. | 84 |
| 1993 | United States | The 1993 Indoor association football tournament United States | Group A streptococci (GAS) | Respiratory tract | Isolates of group A streptococci serotype T28 SOR+ were obtained from five players. The same serotype was found in throat isolates from one player and two relatives. | / | / | 1. Questionnaires were sent to the players 4 weeks after the tournament.  2. Case records for players and family members who had consulted doctors for infected wounds or a sore throat were examined. | 85 |
| 2016 | Netherlands | The 2016 Obstacle Adventure Race Utrecht, Netherlands | Diarrhoea | Gastrointestinal | Of 8,229 persons registered, 1,264 adults reported AG resolved within 48 hours. Of adults who reported AG, 866 met the case definition. All four stool samples tested for viruses were positive for norovirus genogroup I and genotype 2. | Age group, departure time and ingestion of mud were associated with AG. The epidemiological investigation enabled us to identify mud ingestion as the main risk factor of developing AG; no other source of infection was identified. | 1. Racers and adventure race organisers should be aware of the potential risk of inadvertent ingestion of muddy, possibly contaminated, water during the race. For instance, persons preparing the path or participating in obstacle races should not have diarrhoea or vomiting in the two days before the race. In general, planners of obstacle adventure races should consider building slurry field challenges where animal faecal contamination is unlikely. The courses should be pre-routed to avoid areas heavily contaminated with animal faeces. 2. No unwrapped food should be served. 3. Facilities for washing hands with clean water should be available. | An online questionnaire was sent to 700 participants. Stool specimens from six participants and four water specimens were collected from the swimming location. | 86 |
| 2020 | Italy | Italian Serie A Football | COVID-19 | Respiratory tract | / | / | 1. In this context of uncertainty and multiple possible answers to the phenomenon, it is necessary that the football medicine community will establish uniform safe conditions to resume sports activities in the near future, in observance with the principle of ‘maximal caution’. 2. The aim of this letter is a call for action for all the football medicine community to recommend to the football governance the maximal caution on the decision when to restart sport activity. Moreover, a specific protocol to check cardiological, pulmonary and, in general, systemic sequelae of COVID-19 before resuming sporting activities should be considered. | / | 87 |
| 2013 | Uruguay | 2013 a sports competition Montevideo, Uruguay | Group A streptococci (GAS) | Respiratory tract | In November 2013, a large GAS outbreak was reported among 1702 young participants (15~20 years old) from all over the country who came to Montevideo, the capital city of Uruguay, for a sports competition. At least 374 patients with tonsillo-pharingitis were identified (attack rate 21.97%), most between November 8th and 9th. | The epidemic curve suggested an outbreak by a common source, probably food-borne. The meal was provided by a catering service to all participants, except to those living in Montevideo. The epidemiological investigation showed that procedures for preparation, storing, transporting and handling of the dishes were not always according to regulations. | / | / | 88 |
| 2016 | Brazil | The 2016 Rio Olympics  Rio, Brazil | Zika | Vector | / | / | WHO has advised that travelers who are not pregnant may attend the Olympics safely while taking precautions to avoid ZIKV exposure, and that canceling or relocating the event will not alter international ZIKV spread significantly. | Estimated returning travelers' total person-days at risk for transmitting ZIKV after becoming infected during travel, based on a model for ZIKV transmission in Brazil. | 89 |
| 2000 | Malaysia | The 2000 “Eco-Challenge” Borneo Malaysian | Leptospirosis | Water-contact | Of 304 athletes, we contacted 189 (62%) from the United States and 26 other countries. 80 (42%) athletes met the case definition. 29 (36%) case-patients were hospitalized; none died. | 1. On univariate analysis, statistically significant risk factors for illness included kayaking, swimming in the Segama River, swallowing water from the Segama River, and spelunking. By multivariate stepwise logistic regression, only swimming in the Segama River was independently associated with illness. The attributable risk of river swimming was 38%. 2. During Eco-Challenge, regional monthly rainfall totals were approximately 250 mm greater than the average for the previous 3 years. Flooding elevates the water table, saturating the soil with leptospires, preventing evaporation of contaminated animal urine, and potentially promoting the survival of leptospires in surface waters. | 1. Preexposure chemoprophylaxis could be increasingly important. In addition, physicians treating returning travelers should consider diseases such as leptospirosis in patients with a history of water exposures. 2. Prevention strategies for leptospirosis have traditionally relied on protective barriers such as wearing rubber boots and gloves and avoiding high-risk areas.  3. Doxycycline given before or shortly after exposure can reduce illness and death caused by leptospirosis. | 1. A standardized telephone questionnaire was administered, directed at determining demographics, symptoms, duration of illness, previous antibiotic use, and various exposures encountered during the event.  2. 38 serum samples were obtained from a sample of the cohort (who met the clinical case definition) for laboratory testing for various pathogens. | 90 |
| 2015 | United States | The 2015 Special Olympics Los Angeles, United States | Multi-infectious disease | Vector/Respiratory tract/Gastrointestinal/Blood-borne/Water-contact | / | / | The syndromic surveillance system can be customized quickly and flexibly to produce many indicators for medical encounters during mass-gathering events where alternative methods of surveillance may otherwise be unavailable, timeconsuming, or too expensive to implement. Although hospital recruitment for proactive patient tagging was timeconsuming, we demonstrated its potential added value for traditionally challenging applications ofsyndromic surveillance such as mass gatherings. Using syndromic surveillance as a complementary surveillance tool, possibly enhanced with proactive patient tagging, can improve patient detection, expedite outbreak investigations, and reduce the impact of communicable diseases and other health conditions of public health interest. | We queried live databases containing data on ED visits, California Poison Control System calls, and Los Angeles County coroner-investigated deaths for increases in daily counts from July 19 to August 6, 2015. We chose syndrome categories based on the potential for disease outbreaks common to international travel and dormitory settings, morbidity amplified by high temperatures, and bioterrorism threats inherent to mass gatherings. We performed line-list reviews and trend analyses of total, syndrome-specific, and region-specific daily counts, using cumulative sum-based signals. We also piloted a novel strategy of requesting that ED registrars proactively tag Special Olympics attendees in chief complaint data fields. | 91 |
| 2000 | United States | The 2000 Football Game North Carolina, United States | Norwalk Virus | Gastrointestinal | We identified 54 persons with illness among the 108 persons from North Carolina who were interviewed (50 percent). Forty-three of the patients met the definition for a primary case, and 11 were defined as having secondary cases. Seven of the 108 persons who were interviewed did not travel to Florida; of these 7, 4 met the definition for a primary case, and 2 were defined as having secondary cases. | 1. Contact with feces and vomitus.  2. Both fecal–oral transmission and aerosol transmission of vomitus probably occurred, given the intense physical contact and use of bare hands that are characteristic of the game of football. | 1. On the basis of our findings, we recommend that players with acute gastroenteritis be excluded from competition in order to avoid transmitting the disease to other players.  2. Educate players and support staff on appropriate hygiene measures, such as hand washing. | **Epidemiologic Investigation:** We conducted a retrospective cohort study to examine risk factors for diarrhea or vomiting. **Laboratory Investigation:** Four stool samples from North Carolina patients and two from Florida patients were collected within 24 hours after the onset of diarrhea. The samples were cultured for salmonella, shigella, and campylobacter species. Food was collected from a remaining box lunch at the restaurant where it had been prepared and was tested for Norwalk-like virus with a RT-PCR assay. **Environmental Inspection:** Environmental health specialists inspected the restaurant where the box lunches eaten by the team had been prepared. Food-processing procedures were reviewed, and food-storage temperatures were checked on all refrigeration units. | 92 |
| 2012 | Britain | The 2012 Olympics London, Britain | Multi-infectious disease | Respiratory tract/Gastrointestinal | Respiratory and acute respiratory infection indicators peaked during December 2010, concomitant with national influenza activity, as monitored through other influenza surveillance systems. | / | / | 1. The Health Protection Agency, in collaboration with the College of Emergency Medicine, has established the Emergency Department Sentinel Syndromic Surveillance System (EDSSS) to support the public health surveillance requirements of the Games. 2. During the 2004 Athens Games, syndromic surveillance provided early warning of an increase in gastroenteritis cases, which was linked to an outbreak of Salmonella spp., thus alerting infectious disease control teams before traditional surveillance systems had detected significant signals. | 93 |
| 2009 | Lebanon | The 6th Francophone Games Lebanon | Acute respiratory infection,Gastroenteritis,Febrile maculopapular rash | Respiratory tract/Gastrointestinal | 1. Twenty-one febrile respiratory infections were reported from the village clinic. Medical delegates reported 7 other febrile respiratory infections. No clustering was observed. 2. Twenty-three cases of gastroenteritis were reported from the village clinic and 5 from medical representatives the delegations. Bacteriological culture was performed on 11 stool specimens which all tested negative. | / | 1. At the village clinic, the MOPH provided stockpiles of essential drugs and antiviral medications, in addition to sets for specimen collection and personal protective equipment. Any acute respiratory infection case was considered as suspected A(H1N1)2009 and was isolated in a dedicated room. He/she was treated immediately with antivirals while a sample was taken for testing by real-time reverse transcriptase polymerase chain reaction (RT-PCR). Based on the test result, treatment was either continued or suspended. Te management of suspected cases was inﬂuenced by the experience in Serbia earlier that year. 2. Posters in French focusing on hand hygiene, cough etiquete and in fluenza prevention were distributed throughout the Francophone Village. 3. Building partnerships. Several collaborations were essential to carry out these surveillance activities. Expertise and technical support were provided by the World Health Organization (WHO) and the European Centre for Disease Prevention and Control. WHO provided posters and brochures and supported the presence of 2 epidemiologists. Te European Centre for Disease Prevention and Control provided epidemic intelligence. Measles Immunoglobulin M (IgM) serology, water bacteriological testing and stool bacteriological culture were referred to the clinical laboratory of the Rafc Hariri University Hospital. Timely RT-PCR testing for inﬂuenza virus was provided by the research laboratory of the Rafc Hariri University Hospital. Entomological specimens were sent to the Faculty of Arts and Sciences at the American University of Beirut. Food bacteriological testing was undertaken by Fanar Laboratory of the Ministry of Agriculture. | 1. For each participating country, the websites of their health authorities were reviewed in atempt to build their epidemiological profles. In addition, news websites were screened for reported national or local outbreaks. Diseases were then prioritized according to the likelihood of their occurrence during the 6FG. Priority diseases included acute watery diarrhoea, bloody diarrhoea, acute respiratory infection, non-febrile respiratory syndrome and febrile maculopapular rash. 2. Surveillance during the 6FG relied on enhancing the national communicable diseases surveillance, as well as conducting site-specifc surveillance. Official circulars were issued by the MOPH to hospitals in Beirut and Mount Lebanon, requesting them to immediately report all mandatory notifable diseases for the 6FG period. Site-specifc surveillance was initiated at the village clinic. Each medical consultation was documented using a standardized medical form, including information on patient identifcation, disease signs and diagnosis. In addition, the delegations’ medical representatives were asked to fll a daily zero reporting form specifying the number of acute respiratory infections, gastroenteritis and other febrile diseases. | 94 |
| 2020 | Japan | The 2020 Tokyo Olympics Tokyo, Japan | COVID-19 | Respiratory tract | 1. Only a few RTI and gastrointestinal infection outbreaks were found in a review of Gautret et al., which included the Summer and Winter Olympics from 1984 through 2015. In the 2002 Winter Olympics in Salt Lake City, 36 cases of influenza among participants were recorded. During the Vancouver 2010 Winter Olympics in Canada, an epidemic with 82 cases of measles occurred. No major public health incidents occurred during the London 2012 Olympic Games. Only a few cases of RTIs and gastrointestinal illness were reported during this event, but no food-borne illness was directly linked to a Games venue. During the London 2012 Olympics, a total of 47 sexually transmitted infections were diagnosed in 289 visitors, including 8 chlamydia and 15 nonspecific genital infections. There were no new cases of HIV or syphilis diagnosed. During the Sochi 2014 Winter Olympics in Russia, 249 illnesses were reported among the 2788 athletes (the incidence was 8.9 illnesses per 100 athletes). Of those, 58% were caused by infectious diseases. RTIs were the most frequent (63.9%), followed by gastrointestinal symptoms (11%). During this event, 613 illnesses were reported. Infectious diseases affected 56% of ill individuals. RTIs and gastrointestinal symptoms were the most common and occurred in 202 (47%) and 131 individuals (21%), respectively. Although the Rio de Janeiro 2016 Olympics took place during the time of a Zika outbreak, no cases of Zika virus were detected among the athletes and attendees. In 2018, the Pyeong Ghang Winter Olympics in South Korea had the highest number of athletes (2920) and participating countries (92) in the history of the Winter Olympics. During this event, the most common cause of illness was RTIs. A total of 1639 athletes consulted polyclinics, including 1402 (85.5%) visits for illness, with 107 cases of upper RTIs. Common cold was also observed in 42 of 112 members of the Finland team. Also, during the Pyeong Ghang 2018 Winter Olympics, a norovirus outbreak emerged a few days before the event began. This outbreak affected 172 volunteers staying at hostels but only 4 athletes. 2. During the Rio 2016 Paralympics season, athlete delegations were approximately 60% physical-motor deficient, 25% visually impaired, and 5% intellectually disabled [35••]. They presented twice the amount of total illness in comparison to the Olympic athletes, and RTIs were the most frequent. Controlling disease in Paralympic athletes is relatively more complex. It is estimated that between 10 and 20% of these athletes are at risk of aggravating symptoms if they are infected by COVID-19. | 1. Several MGs have been identified as the source of infectious diseases that have spread globally. 2. The strong infectivity of SARSCoV-2 and rapid transmission even from asymptomatic carriers during the long incubation period have been previously described. Because the incubation period of the virus is long (up to 14 days), controlling viral dispersion seems to be difficult.  3. The relatively close contact between participants, including athletes and staff, spectators, and journalists, could increase the spread of COVID-19. Moreover, there was a high risk of globalization of virus transmission by travellers. Screening at airports is feeble, and nearly 46% of infected travellers cannot be identified. 4. Regarding athletes, the overtraining syndrome and high glycemic diet are often associated with chronic diseases. Those with chronic diseases are more susceptible to SARS-CoV-2 infection and aggravation or complications of COVID-19. Athletes are exposed to a higher risk of infection because of their compromised immune system. | In collaboration with local health authorities, organizers should agree in advance about the circumstances in which risk mitigation measures would need to be reinforced. | / | 95 |
| 2003 | California | The 2003 160-km Western States Endurance Run Olympic Valley, California | Upper respiratory tract infection (URTI) | Respiratory tract | Nearly 1 in 4 runners reported an (upper respiratory tract infection) URTI episode during the 2-week period following a 160-km race, and the decrease in salivary IgA secretion rate was significantly greater in these runners compared to those not reporting URTI. | / | / | / | 96 |
| 2016 | Brazil | The 2016 Rio Olympics  Rio, Brazil | Zika | Vector | / | 1. At present significant global attention is firmly focused on Zika and the threat posed by mosquito transmission of this virus. 2. Reports of Zika transmission via sexual intercourse have, not surprisingly, increased and sustained media interest in the story. | / | / | 97 |
| 1997 | South Africa | The 1997 Adventure Race South Africa | Rickettsia africae | Vector | 13 cases of R. africae infection diagnosed in France that occurred in competitors returning from an adventure race in South Africa. | Patients in southern Africa, or those returning from a visit to this area, present with a history of a tick bite, fever, headache, eschars, and regional lymphadenopathy. | When patients in southern Africa, or those returning from a visit to this area, present with a history of a tick bite, fever, headache, eschars, and regional lymphadenopathy, physicians need to consider ATBF and instigate appropriate dianostic procesures and treatment. | 1. Serum samples and heparinized and EDTA (1 mg/mL)–treated blood samples were collected from each patient, and an eschar biopsy specimen was requested from each patient.  2. The clinical and epidemiological features of each patient were recorded for comparison with those of the eight cases of proven R. africae infections that have been reported so far. | 98 |
| 2020 | Japan | The 2020 Tokyo Olympics Tokyo, Japan | Multi-infectious disease | Gastrointestinal/Blood-borne | / | / | The NIID will use the live samples to validate tests it has developed to assess whether a person with one of the viruses is still infectious, says Saijo. The tests measure whether the person is generating antibodies that are capable of neutralizing the virus in question, which would suggest that they are recovering, and not infectious, he says. If there is a person with one of these viruses at the games, such a test could provide valuable information for assessing whether they can be discharged from hospital, he says. | / | 99 |
| 2007 | United States | The 2007 International youth sporting event Pennsylvania, Michigan and Texas, United States | Measles | Respiratory tract | Combined participant and spectator attendance for the event was approximately 265,000. 7 measle cases. | Persons born before 1957 might also remain susceptible to measles. | 1. The small number of identified cases in this outbreak, despite the large number of exposed persons, demonstrates the value of maintaining high measles vaccination coverage in the U.S. population through adherence to routine vaccination recommendations. This outbreak also highlights the continuing importance of promoting measles control and elimination in other countries and sustaining strong surveillance and response measures in the United States. 2. All persons aged >12 months without adequate evidence of immunity should receive 1 or 2 doses of measles or MMR vaccine in accordance with current recommendations. Vaccination records should be actively maintained for adults and children. Although 2 doses of measles vaccine are 99% effective, cases can still occur in appropriately vaccinated persons, as observed in this investigation (cases 6 and 7). | / | 100 |
| 1998 | United States | The 1998 Triathlons Wisconsin and Illinois, United States | Leptospirosis | Water-contact | 3 athletes were hospitalized with an acute febrile illness. One of three athletes had acute renal failure. Two of the athletes had participated in a triathlon held in Madison, Wisconsin. | / | / | A total of 639 triathlon participants from 39 states had been interviewed by telephone using a standardized questionnaire. | 101 |
| 2014 | Brazil | The 2014 FIFA World Cup and the 2016 Olympic Games Brazil | Mayaro virus | Vector | / | 1. This first description of a MAYV outbreak in an urban setting and the recent report that the highly anthropophilic mosquito Aedes aegypti may also act as a vector for MAYV underscores the possibility that MAYV will increasingly be introduced into urban settings and thus confer a risk for visitors to these World Cup host cities. In addition, visitors will be at risk for any of the other endemic arboviral infections in Brazil, including Oropouche virus, Venezuelan equine encephalitis and Yellow Fever.  2. The widespread distribution of Aedes mosquitoes over Brazil also poses a significant threat for introduction of new arboviruses by visiting fans. This risk seems particularly high for Chikungunya virus (CHKV). | Prevention consists of avoidance of mosquito bites. | / | 102 |
| 1992 | United States | Professional Football | HIV | Blood-borne | / | / | / | We developed a straightforward model for estimating the probability of HIV transmission during a single contact between two randomly chosen professional football players in one game. We defined this probability to be the product of four factors (probability of HIV transmission = prevalence of persons infected with HIV X risk for percutaneous HIV transmission X risk for laceration in an opponent X risk for any bleeding injury per game for each player). | 103 |
| 2020 | Japan | The 2020 Tokyo Olympics Tokyo, Japan | Multi-infectious disease | Vector/Respiratory tract/Gastrointestinal/Blood-borne/Water-contact | / | Communicable disease risks and precaution for heat exposure. | 1. Visitors should be aware of the risk of heat-related illness and should take special precautions for heat stroke, by limiting the time they spend outside in hot temperatures, regularly drinking water and covering and cooling the skin. In addition, the risk of taking particular drugs, including blood pressure medications, which may exacerbate heat-related illness, should be considered. Visitors experiencing any symptoms in this regard should consult medical staff immediately. 2. We recommend therefore, that all visitors attending Tokyo 2020 ensure that their routine vaccinations are up-to-date.  3. Food and waterborne diseases will also need to be considered due to the lowered hygiene standards that tend to occur during mass gathering events.  4. General good hygiene measures, including hand hygiene, should be encouraged throughout the Olympics.  5. Sexually transmitted infections also pose a risk and practicing safe sex behavior is recommended; especially in light of the recent increase in syphilis cases in Tokyo.  6. Furthermore, since the climate in Tokyo during summer is expected to be very hot and humid, visitors should be aware of heatrelated illnesses and health promotion programs should be expanded so that all visitors remain cognizant of heat related illness risks.  7. It would also be useful to increase the number of multilingual triage clinicians, particularly English-speaking nurses, which can be placed within emergency departments across Tokyo during the Olympic period to provide first contact services and coordination of emergency care among non-Japanese speaking visitors to Tokyo. | We reviewed up-to-date surveillance reports published by the National Institute of Infectious Diseases and Tokyo Metropolitan Infectious Disease Surveillance Center. | 104 |
| 2012 | Britain | The 2012 Olympics London, Britain | Multi-infectious disease | Vector/Respiratory tract/Gastrointestinal/Blood-borne/Water-contact | 1. Illnesses of the respiratory system were the most common illnesses recorded (39.4%) followed by illnesses of the digestive system (15.8%), skin and subcutaneous system (11.8%), genitourinary system (8.8%), and nervous system (7.3%).  5. Infection accounted for 40.8% of all illnesses, whereas environmental conditions accounted for 21.8% of illnesses. It is of interest to note that 7.8% of all illnesses were attributed to pre-existing conditions. Most illnesses for the specific systems were due to infections; 50% of all respiratory illnesses were deemed to be due to infection, whereas infection also accounted for 44.4% of skin and subcutaneous tissue illness and more than 82% of genitourinary illnesses. | / | / | 1. Chief medical officers or their designated staff entered daily illness encounter data on the **WEB-IISS**, which was specifically developed for this purpose. 2. The WEB-IISS incorporated several unique features that allowed more clinical detail on injury and illness as well as exposure data to be captured. The system was further adapted to be applicable to athletes with impairment, and translations were made available. Data input via the WEB-IISS was facilitated through desktop or laptop computer interface, tablet, or smart phone. 3. WEB-IISS reporting system was implemented for the first time during the London 2012 Paralympic Games. The WEB-IISS reporting system allowed us to capture more data than has been collected to date and, as such, provides valuable information for team physicians who work with teams of Paralympic athletes. | 105 |
| 2020 | German Bundesliga | The 2020 Men's Football (soccer) Competition German Bundesliga | COVID-19 | Respiratory tract | 1. Altogether, 1702 individuals were regularly tested (898 from the Bundesliga and 804 from the Bundesliga 2). This number included 1079 players (550 and 529) and 623 officials (348 and 275). Eight players and four officials from a total of seven clubs were tested positive during one of the first two rounds (before the onset of team training) and put into quarantine, that is, they were isolated from all other team members.Three of all positive cases were later proven to be remnants of earlier COVID-19 infections based on medical documents provided by the team doctors. 2. One hundred and sixty-five matches were played and this resulted in no player or official: (1) suffering a documented infection during the season; (2) being infected with SARS-CoV-2 (as shown by RT-PCR) throughout the season; or (3) seroconverting (IgG antibodies for COVID-19). | age >65 years, pre-existent diseases of the respiratory or cardiovascular systems | / | Team physicians monitored players’ symptoms using a questionnaire provided in a mobile app. Players were also encouraged to make direct phone contact with their team doctors as needed. The questions covered all typical COVID-19 symptoms (cough, fever, smell and taste sensations, difficulties breathing, etc) as well as general ones for respiratory infections, for example, sore throat, running nose, malaise. Team doctors were free to use their self-designed questionnaires or applications. | 106 |
| 2019 | Peru | The 2019 Pan American games Peru | Multi-infectious disease | Vector/Respiratory tract/Gastrointestinal/Blood-borne/Water-contact | / | 1. Travelers may not only acquire STIs, during travel, they may transmit them on return to their home countries and risk the international dissemination of agents with genetic diversity and different antibiotic-sensitivity patterns.  2. Travelers not only may introduce pathogens from their own country of residence but also, because of the propensity of a high proportion of travelers who travel to multiple countries over time, may serve as conduits for multinational dissemination of pathogens. | 1. As recommended by the World Health Organization (WHO), countries should vaccinate to maintain homogeneous coverage of 95% with the first and second doses of the measles, mumps, rubella (MMR) vaccine in all municipalities.  2. Apart from international travelers, it is also recommended to vaccinate local, at-risk populations (without proof of vaccination or immunity against measles and rubella), such as healthcare personnel, and transportation (hotels, airports, taxi drivers, and others), staff of the sports areas during the Pan American Games.  3. maintain a local stock of MR and/or MMR vaccines and syringes for control of imported cases, as well as to identify population flows (arrival of international travelers) and internal flows (displaced populations) related or not to the Pan American Games, including indigenous populations, in order to facilitate access to vaccination services according to the national schedule.  4. Before and especially during the Pan American Games, local health authorities should strengthen the epidemiological surveillance of measles to achieve timely detection of all suspected cases of measles in public and private healthcare facilities and to ensure that samples are received by laboratories within 5 days of rash onset (viral detection window) and that laboratory results are available in a timely manner.  5. They should provide a rapid response to imported measles cases to avoid the re-establishment of endemic transmission, through the activation of rapid response teams trained for this purpose and by implementing national rapid response protocols when there are imported cases. Once a rapid response team has been activated, continued coordination between the national and local levels must be ensured, with permanent and fluid communication channels between all levels (national, sub-national, and local). 6. Before the Pan American Games, health authorities should define which is the adequate hospital case management and facilities, to avoid nosocomial transmission, with appropriate referral of patients to isolation rooms (for any level of care) and avoiding contact with other patients in waiting rooms and/or other hospital rooms, in the case of outbreaks occurring before or during Pan American Games.  7. Day-time, mosquito bite prevention with protective clothing and repellent on exposed skin are highly recommended.  8. Accommodation with screens and air conditioning for prevention of some vector-borne infections will be also useful.  9. Pretravel counseling on sexual behavior should be an essential part of prevention, including hepatitis B vaccination as well as advice on the use of condoms.  10. Hepatitis B vaccination should be recommended for those without previous vaccination, and especially for those individuals probably engaging in casual sex, getting a tattoo or piercing, having dental surgery or other medical procedure all risky practices which should be avoided during travel of condoms is recommended for this and other sexually transmitted infections. | / | 107 |
| 2015 | Latvia | The 2015 international ice hockey tournament Latvia | Salmonella enteritidis infection | Gastrointestinal | Team leaders and national public health authorities reported a total of 250 cases (154 suspected and 96 confirmed) from 48 teams. The AR over the course of five tournament weekends was 5% (250/5290). | / | 1. The Latvian Food and Veterinary service requested the food business operator to improve the hygiene in the kitchen. In addition, participants of the Riga Cup 2015 and of other ice hockey events that took place in arena A were informed about the ongoing outbreak. They were recommended to follow good hand hygiene practices and consume only heat-treated foods. 2. Substantial coordination with clear roles and responsibilities is needed in international outbreak investigations. 3. Timely communication and data sharing between epidemiologists, microbiologists and food safety authorities is challenging in international investigations. Regular teleconferences for information exchange and access to information sharing systems like EPIS-FWD for data sharing and data pooling between the affected countries are essential to progress in this type of investigations. As uploading information to EPIS-FWD is additional work for member states, clear guidance should be given to the countries on how, when and what data to upload. | 1. To find the cases, we acquired each participating team’s contact person’s e-mail from the organisers of Riga Cup 2015.  2. The outbreak team at the Norwegian Institute of Public Health also used the Norwegian Surveillance System for Communicable Diseases (MSIS) to identify the confirmed cases and also contacted the team leaders to find suspected cases. | 108 |
| 2011 | New Zealand | The 2011 Rugby World Cup New Zealand | Sexually Transmitted Infections | Blood-borne | 1. Although there was no statistically significant increase in clinic attendance or STI diagnoses during the RWC compared with previous years, in these four cities, 151 individuals of 2079 attending SHCs for a new concern reported RWC-related sex. The most frequently diagnosed STIs were chlamydial infection (Chlamydia trachomatis), genital warts and genital herpes. Most attendees (74%) who had RWC-related sex had consumed three or more alcoholic drinks; 22% had used a condom. Seven percent of women reported nonconsensual sex. | / | 1. These findings highlight issues that are amenable to prevention. The continued promotion of condoms as well as a reduction in the promotion and availability of alcohol at such events may reduce sexual health risks as well as other harm. | / | 109 |
| 1997 | Belgium | The 1997 youth football tournament Belgium | Meningococcal | Respiratory tract | 11 cases occurred during and after the tournament. | / | 1. Surveillance and control need to be linked, with microbiologists and epidemiologists working together across Europe.  2. Reviewing differing prevention and control measures, including vaccination strategies. | 1. Notification systems for meningococcal disease are broadly similar in most European countries. The dignosing physician should inform the local health department. These departments are generally responsible for initiating preventative measures; every case of meningococcal disease is regarded as requiring a response. Notifications are transmitted to regional or national health departments.  2. Information shared through international networks could lead to action. | 110 |
| 1995 | Poland | The 1995 Junior World Rowing Championships Poznan, Poland | Salmonella | Gastrointestinal | 8 athletes and 8 coaches fell ill. Throughout the championship, 104 consultations were related to gastroenteritis. | Poor standards of hygiene, such as inappropriate storage, cross contamination from uncooked to cooked foods, or inadequate cooking. | 1. Assess accommodation and catering arrangements and seen if they were adequate.  2. There should be a system of notification of any infectious conditions occurring during the competition and that there should be frequent medical meetings. 3. athletes without their own doctor should be encouraged to consult one of the other doctors present. | Illness statistics at the end of the championship from each team. | 111 |
| 1992 | Spain | The 1992 Olympics Barcelona, Spain | Pneumococcal | Respiratory tract | / | / | 1. Physicians should consider the use of pneumococcal vaccine, particularly for elderly persons and travelers with risk factors for pneumonia and invasive pneumococcal disease.  2. Physicians should also be aware that resporatory tract or other infections in travelers returning from Spain may be caused by antibiotic-resistant S.pneumoniae. | / | 112 |
| 2012 | British | The 2012 London Olympics London, British | Measles | Respiratory tract | / | / | CDC has posted a “Measles Update” for travellers, which advises all travellers to be vaccinated against measles and gives examples of recent outbreaks, including cases in several countries in Europe. | / | 113 |
| 1994-2009 | United States | The US Open Tennis Championships | Multi-infectious disease | Vector/Respiratory tract/Gastrointestinal/Blood-borne/Water-contact | / | 1. High training loads coupled with insufficient recovery and nutritional replenishment may increase the likelihood of “overreaching,”9,23 which if not adequately managed may impact the quality of sleep and increase the risk of minor infections. 2. Risk factors such as the arduous travel commitments inherent in professional tennis, as well as the unpredictability of professional competitive tennis, may interfere with optimal physical conditioning, practice, access to treatment facilities and personnel, circadian rhythm (and sleep patterns), and optimization of recovery. 3. Risk factors for ENVIR illness include, but are not limited to, lack of sleep, improper nutrition or supplementation, dehydration, poor acclimatization, use of medications, and overtraining. | / | In this cohort observational study, illness rates were examined in tennis players participating in the US Open Tennis Championships over a 16-year inclusive period from 1994 to 2009. Data of cases were included if a medical issue within a player required attention by the tournament medical staff and/or physician during the US Open Tennis Championships. | 114 |
| 2010 | Canada | The 2010 Winter Olympics Vancouver, Canada | Not specified | Not specified | / | / | 1. Purchased 5000 doses of seasonal flu vaccine for its staff, while Vancouver Coastal Health is picking up the vaccine tab for an estimated 20 000 volunteers. 2. Organizers have also planned a series of measures to ensure air, water and food quality, as well as prevent disease outbreaks. Taunton says air quality will be monitored at both indoor and outdoor venues. And to prevent asthma and exerciseinduced asthma, VANOC has purchased 19 electric ice shavers to replace the traditional propane powered zambonis. 3. Water quality will be monitored in both Vancouver and Whistler, while food inspection officials will regularly inspect local eateries and restaurants to prevent the spread of enteropathic illnesses, such as Norwalk virus. Disease and injury reports from all hospitals, polyclinics and Olympic venues will be tracked and made available on the VANOC website. 4. Organizers will also distribute more than 100 000 condoms at polyclinics and designated Olympic hotels, and offer free HIV testing to athletes and their families. | / | 115 |
| 2016 | Brazil | The 2016 Rio Olympics  Rio, Brazil | Zika | Vector | / | / | 1. The best course of action is to inform the population regarding protective measures at the individual level. The best action is a set of practices such as the use of mosquito repellents to avoid bites, which is still assumed to be the main infection route, and the use of condoms to avoid the possibility of sexual transmission, for which evidence is increasing, along with public action by Brazilian authorities to minimize exposure to Ae. aegypti, which is likely to be facilitated by climate factors during Rio de Janeiro’s winter time. 2. Pregnant women, on the other hand, should avoid travelling to countries with known Zika trasmission, since the severity of the possible outcome in case of an unlikely infection is overwhelming. Nonetheless, the recommendation for the general public is to attend normally, while paying attention to the instructions from WHO and the Brazilian Health authorities. | Using a mathematical model, Massad et al. (2014) estimated three to 59 symptomatic dengue cases. Ultimately, only three cases of dengue infected tourists were reported during the World Cup. For the Olympic Games, Ximenes et al. (2016) estimated at a worst case scenario 23 to 206 dengue cases among tourists. Such estimate is based on the historical dengue reports from 2007. | 116 |
| 2010 | / | The 2010 Super 14 Rugby Union tournament | Multi-infectious disease | Respiratory tract/Gastrointestinal/Blood-borne | The incidence of illness in the cohort was 20.7/1000 player days (95% CI 18.5 to 23.1) with the highest incidence of illness in the respiratory system (6.4: 95% CI 5.5 to 7.3), gastrointestinal system (5.6: 95% CI 4.9 to 6.6) and the skin and subcutaneous tissue (4.6; 95% CI 4.0 to 5.4). Infections accounted for 54.5% of all illness and 26.1% of illness resulted in time loss of ≥1 day. | 1. The longer duration of the tournament (16 weeks vs 2–4 weeks), high-intensity weekly matches and training sessions, travelling across multiple time zones (up to 11 h difference between South Africa and New Zealand venues), varying climatic and environmental conditions in different countries and playing venues, and the nature of the sport (rugby union vs football). 2. Furthermore, it is common for all Super Rugby franchises to have their own training facility base where players are in close proximity to one another for 4–6 h per day for about 4 days per week. At these facilities, players eat two meals together (breakfast and lunch) and make use of communal shower and ablution facilities. This environment could also facilitate the spread of infection in teams. | Team physicians should be encouraged to educate both players and accompanying staff to report symptoms that could be associated with any infective illness as soon as possible so that appropriate treatment and isolation where possible may be initiated. This may also be of particular importance in symptoms of skin and ear disease, which were often only reported after 1–3 days. | Data collection took place on a daily basis during the competition. Each team physician was requested to complete a ‘daily medical illness log’ for each player during the competition. Detailed information about all the components of the study was provided to the team physicians of the South African (five teams) and some of the New Zealand (three teams) participating teams. All the players (n=259) participating in the eight teams were approached (28–36 players per team) to participate in this prospective study through their team physicians who were given detailed information about the nature of the research study. Each team physician was able to explain the details of the study as well as all the potential risks and benefits of the study by providing players with a detailed subject information sheet. Written informed consent was obtained from players to participate in the study. | 117 |
| 1988 | Korea | The 1988 Seoul Olympics Seoul, Korea | Japanese encephalitis | Vector | / | / | Strict prevention and control measures to the minority of travelers who meet all criteria: (1) those traveling to a country in which the disease is endemic,(2) those traveling during the season in which the disease is endemic, and (3) those planning a stay of at least two weeks and up to four weeks in a rural area. | / | 118 |
| 2016 | Brazil | The 2016 Rio Olympics  Rio, Brazil | Yellow fever | Vector | / | / | Mass gathering organisers must assure community and individual level availability of vector repellents and encourage and monitor their use routinely. Beyond the mass gathering host countries, where adequate vectors and susceptible human populations are present, vigilance, access to a stockpile of vaccines, and risk communication are critical to minimise and contain sporadic outbreaks. | / | 119 |
| / | / | International Football Game | COVID-19 | Respiratory tract | / | / | / | In this study, we explored the feasibility of using tracking data from a football match to assess interpersonal contact between individuals by calculating two measures of respiratory exposure. The dynamic tracking positioning of all players and referees during one international football match was analyzed.Tracking data can be used to assess respiratory exposure to interpersonal contact in team sports, such as football. The measures of exposure calculated can be used to the prompt identification of high-risk contacts of COVID-19 cases during a match or a training session, but also the risk stratification of different sports and physical activities. | 120 |
| 2016 | Brazil | The 2016 Rio Olympics  Rio, Brazil | Zika | Vector | / | The Olympics attracts mostly young healthy adults from middle and upper-middle income groups who live in developed countries. Such visitors are less likely to have been exposed to arbovirus infections and less familiar with mosquito bite prevention. Sexual transmission of Zika virus from commercial sex workers with asymptomatic infection might also be a possibility for those who attend the Olympics. | 1. Targeted promotion of options for personal mosquito bite protection—eg, the use of insect repellents, protective clothing, including long-sleeved shirts and trousers, insecticide-treated mosquito nets, and air conditioning in residences.  2. Despite the uncertainty about sexual transmission of Zika virus, the promotion of safe sex and provision of condoms is beneficial from a broader health perspective.  3. Health-care providers can be encouraged to use travel health visits as an opportunity to emphasise the need for personal protection against mosquito bites and sexual transmission. 4. Additionally, by training athletic coaches on prevention of Zika virus transmission, their frequent contacts with athletes can be used to remind athletes about the need for compliance with public health advisories.  5. Advice on personal protection can be reinforced at points of departure and arrival in home and host countries. Increasing the availability and distribution points of methods to prevent mosquito bites is also crucial. | / | 121 |
| 2014 | Brazil | The 2014 FIFA World Cup Brazil | Dengue | Vector | / | The mass gatherings and predictable movement of fans should be a help to campaigns promoting personal protection, but they may also increase the potential for dengue transmission. Supporters may inadvertently introduce into Brazil new dengue genotypes to which local immunity is low, and the assembly of large non-immune, and hence susceptible, populations could fuel transmission in the event of an outbreak. | 1. The Brazilian authorities should implement aggressive vector control in April and May, particularly around the northern stadiums, to decrease the number of dengue-transmitting mosquitoes. They can target adult Aedes mosquitoes through fogging (the use of aerosol formulations of insecticides that disperse efficiently) and can interrupt breeding by clearing sites at which the mosquitoes lay their eggs — water collected in discarded rubbish, for example.  2. So avoiding mosquito bites is the best precaution. Select accommodation with screened windows and doors and air conditioning; use insecticides indoors; wear clothing that coversthe arms and legs, especially during early morning and late afternoon, when the chance of being bitten is greatest; and apply insect repellent to clothing and exposed skin. | / | 122 |
| 2002 | United States | The 2002 Winter Olympics Salt Lake City, United States | influenza | Respiratory tract | The only significant event of public health interest that was noted during the surveillance period was an increase in influenza during the Games. | / | / | 1. Hospital-based infection control professionals led a multi-disciplinary team to develop a computer rule–based system that relies on the patient’s electronic medical record. The system was deployed at a large hospital in Salt Lake City during the 2002 Winter Olympic Games, and it was accessed 3 times a day to perform surveillance. Daily reports were provided to local PH agencies after preliminary investigation of the alerts. 2. The positive predictive value of the rules varied with a high value (89%) noted for identification of pneumonia from chest radiograph reports by natural language–processing algorithms. | 123 |
| 2016 | Brazil | The 2016 Rio Olympics  Rio, Brazil | Dengue | Vector | / | With a high prevalence of flavivirus-naive children, the proportion of those admitted to hospital with breakthrough dengue infections in vaccinated 2–5-year-old children was five times higher than in placebo controls. WHO have labelled this instance of vaccine-enhanced disease as “currently not understood”. | Nevertheless for now, tourists, many of whom will be flavivirus naive, should avoid dengue vaccination. | / | 124 |
| 2014 | Brazil | The 2014 World Cup and the 2016 Olympics Brazil | Multi-infectious disease | Vector/Respiratory tract/Gastrointestinal/Blood-borne/Water-contact | / | / | 1. All travelers to Brazil be vaccinated for hepatitis B.19 Travelers should have as many doses of these ries as possible before travel. An accelerated regimen and a hepatitis A and B combined vaccine are available. 2. The CDC recommends typhoid fever vaccine for all travelers to Brazil. No vaccine is available for paratyphoid fever. Other preventive measures include food and beverage precautions. 3. Yellow fever should be suspected when an unimmunized traveler returns with fever from an endemicarea. Clinicians suspecting yellow fever should contact their state or local health department or call the CDC Arboviral Disease Branch Treatment is exclusively supportive. Yellow fever vaccine is recommended for all travelers 9 months or older travelling to areas within Brazil with risk of yellow fever virus ransmission. Clinicians should review each traveler’s medical history and itinerary to evaluate risk of yellow fever disease vs risk from vaccine. Clinicians should educate travelers on preventive measures against mosquito bites, including using insect repellents, wearing long-sleeved permethrin-treated clothing, choosing rooms with air conditioning or intact screens, and sleeping under an insecticide-treated bed net. 4. Because preexposure vaccine is not entirely protective and the disease highly fatal, health care practitioners should educate travelers about animal avoidance and the importance of prompt health care for postexposure prophylaxis and immediate and thorough wound cleaning with soap and water should any bite occur. 5. Medical practitioners should advise travelers about malaria chemoprophylaxis based on travel itineraries. Health care practitioners should inform travelers to urgently seek medical care if they develop fever following travel to a malaria-endemic area. A rapid malaria antigen detection test along with three blood smears at 8-hour intervals should be evaluated for parasites. Assistance in diagnosis and treatment can be obtained from an infectious diseases or tropical medicine clinician, as well as from the CDC malaria branch. 6. Health care practitioners should educate travelers on the importance of frequent handwashing with soap and water and use of alcohol-based hand sanitizers to decrease their risk of gastrointestinal illness. Other recommended approaches to reduce risk include food and water precautions and the use of nonantimicrobial drugs for prophylaxis.39 Freshly cooked food that is served hot is less likely to be contaminated than foods that have been sitting out. Travelers should avoid raw foods, such as salads, unpeeled fruits, or uncooked vegetables. Tap water may contain viruses, bacteria, or parasites and may be unsafe for drinking, preparing food, or for making ice. Travelers may choose to boil, filter, or otherwise treat water for consumption,although this may not remove chemical contaminants. Choosing unopened, factory-sealed beverages and avoiding ice in drinks may decrease the risk of travelers’ diarrhea. 7. Practitioners should educate travelers on vector prevention practices, such as those described in the yellow fever section. Diagnosis usually involve sidentification of the parasitesinaskinbiopsy. A number of treatment regimens are available depending on the species identified. 8. Health care practitioners should advise travelers to avoid unprotected sex with new partners while traveling. Condoms can decrease risk and should be purchased prior to departure to ensure they are of sufficient quality. Assessment of a traveler with a suspected sexually transmitted disease includes screening for others such as human immunodeficiency virus and hepatitis B. | / | 125 |
| 2007 | Canada | A 2007 mountain bike race British Columbia, Canada | Campylobacteriosis | Gastrointestinal | Based on routine surveillance, 32 C. jejuni laboratory-confirmed cases were identified and known to be associated with the bike race. However, of the racers who completed the questionnaire, 25 identified themselves as having laboratory-confirmed C. jejuni. | 1. Campylobacter bacteria are most commonly found in poultry and cattle but may also be found in domestic animals, swine, sheep, rodents, and birds. People can become infected through contact with infected animals or animal waste and through ingestion of the organism in contaminated food and water. 2. Outbreaks have been reported in association with undercooked poultry, unpasteurized milk and contaminated water supplies. 3. We show that drinking cups of water was only significantly associated with illness as a result of the role it played in mud ingestion. Racers reported inserting muddy fingers inside the water cups and photographs from race day depict racers whose faces were completely covered in mud, explaining how contamination of drinking water could occur. | 1. Racers and organizers should be educated on the potential risks of ingesting mud/muddy water from the race course. Recommendations were given to race organizers, including closing the trail to domestic animals prior to the race and reviewing the trail for any obvious signs of environmental contamination such as the use of manure or other excessive amounts of animal waste and septic tank spillage.  2. In addition, clean running water should be available at stations to allow racers to clean mud off of hands and faces prior to eating and drinking. An alternate form of water delivery, such as bottles of water instead of paper cups, which are less easily contaminated, should also be considered. We also recommended that racers use front and rear fenders to reduce splashing of mud up onto their and other riders’ faces, respectively. | 1. In-depth interviews and a visual assessment of the environment were conducted to understand relevant exposures during the event that may have predisposed race participants to infection. Interviews were conducted with 15 ill individuals, as well as volunteers, race organizers, community members, a park ranger and waste disposal services. 2. Microbiological investigation: Initial samples of creek water accessible from the course and bottled drinking water served during the race were collected and tested soon after the 16 June race. | 126 |
| 2020 | Japan | The 2020 Tokyo Olympics Tokyo, Japan | Zika | Vector | / | / | Japan need to strengthen its public health responsivity for these emerging arboviral diseases, especially for dengue in urban areas. Further studies will be needed, including the identification of environmental factors that may contribute to these viral epidemics. | To compare the risk of infection by arboviruses transmitted by Ae. albopictus mosquitoes, the reproduction number for each of three arboviruses was estimated under the environmental conditions associated with the 2014 dengue outbreak in Tokyo, and additionally under conditions assuming a daily mean temperature elevation of 2° C. The basic reproduction number R0 was calculated using the classic Ross-Macdonald model | 127 |
| 2016 | Brazil | The 2016 Rio Olympics  Rio, Brazil | Zika | Vector | / | / | Although publichealth agencies have advised pregnant women to avoid countries with active Zika transmission owing to the threat of birth defects, there are much more pertinent threats to the average visitor to Brazil, including dengue virus and random street violence. | / | 128 |
| 2010 | South Africa | The 2010 FIFA World Cup South Africa | HIV | Blood-borne | / | / | While the distribution of condoms and messages about safer sex might have contributed towards this success, future public health programmes focusing on sex work and HIV prevention during international sporting events such as the 2012 Olympic Games in London and the 2014 FIFA World Cup in Brazil should be based on evidence, not mediadriven sensationalism that further heightens discrimination and vulnerability of sex workers. | We conducted a three-wave telephonic survey of female sex workers in the last weeks of May (pre-World Cup), June (during the World Cup) and July (post-World Cup) 2010. A sampling frame was constructed, by listing all sex worker profiles published on www. sextrader.co.za, a website with national coverage containing over 1000 profiles of sex workers. | 129 |
| 1993 | Puerto Rico | The 1993 XVII Central American and Caribbean Sports Games Puerto Rico | respiratory tract infection/skin/gastrointestinal/genitourinary | Respiratory tract/gastrointestinal/Blood-borne | Of 180 patients with respiratory diagnoses, 71(39%) were athletes whose most frequent diagnoses were upper respiratory tract infection (33) and pharyngitis (23). During the games, acute infectious conjunctivitis was diagnosed in 12 persons, including nine support staff and three athletes. | / | Because of concern about the potential for acute infectious conjunctivitis spread, the nine support staff were provided treatment and asked to leave the game; the three athletes were treated and interviewed by epidemiologists to detect additional cases. | Physicians provided medical care at the athletic village hospital, where an epidemiology unit conducted surveillance while the village was open. | 130 |
| 2016 | Brazil | The 2016 Olympic Games Rio de Janeiro, Brazil | Zika | Vector | / | The Zika virus infection caused by Aedes mosquito bites. | / | Mathematical models were used to calculate the risk of foreign tourists being bitten by an Aedes aegyptii mosquito, the predominant species in Rio, during the Olympic Games (5–20 August) 2016 in Rio de Janeiro. | 131 |
| 1999 | South Africa | An 1999 international sports event Johannesburg, South Africa | Shigella fIexneri | Gastrointestinal | A total of 578 children were involved. Of the 361 children who returned questionnaires, 134 were affected by an acute-onset emetic-type illness, while 53 children developed diarrhoea. Consumption of fruit juice was associated with acute illness, while diarrhoea was associated with the consumption of maize-meal porridge (pap) and chicken stew. | / | Guidelines for monitoring the supply and distribution of food. to future similar events should be established. Furthermore, hospitals should have protocols in place to deal with such outbreaks in a manner that facilitafes epidemiological investigation. | 1. On the night of the outbreak, staff from the private laboratory were alerted and interviewed several children to ascertain details of food consumed and predominant symptoms and to establish a case definition. On the basis of this information, a questionnaire was distributed to ask parents to assist their children in its completion. 2. Environmental investigation: After the outbreak all venues at which food was being served were visited on a daily basis throughout the games and random sampling of food was undertaken. 3. Microbiological and chemical analysis: random food samples taken from all venues where food was served to identify any further contamination. These samples were stored for 4 days so that complete analysis could be conducted should a second outbreak occur. | 132 |
| 2016 | Brazil | The 2016 Rio Olympics  Rio, Brazil | Zika | Vector | / | 1.The epidemic of Zika virus (ZIKV) occurred in South America, particularly in Brazil, the site of the 2016 Summer Olympic and Paralympic Games. 2.As of April 2016, there had been no reported cases of locally acquired ZIKV in the European Union, but 452 cases in returning travellers. | This study showed that fear of contracting ZIKV is not a major deterrent for travelling to high-risk areas. Pregnant women are appropriately concerned about the risk of ZIKV. Studies modelling the further spread of ZIKV need to account for these results. | / | 133 |
| 2008 | China | The 2008 Olympics Beijing, China | Multi-infectious disease | Respiratory tract/Blood-borne/Gastrointestinal | 1. For patients seen during travel, the most frequent syndromes were respiratory (365 per 1,000 ill travelers), injury (126 per 1,000), dermatologic (includes animal bites; 103 per 1,000), psychological (84 per 1,000), systemic febrile illness (79 per 1,000), and acute diarrhea (61 per 1,000).  2. For patients seen after travel to China, the most frequent syndrome groupings were dermatologic (includes animal bites; 226 per 1,000), acute diarrhea (182 per 1,000), respiratory (137 per 1,000), systemic febrile illness (92 per 1,000), non-diarrhea gastrointestinal illness (92 per 1,000), and chronic diarrhea (69 per 1,000). | / | 1. The pre-travel consultation is an important opportunity for health care providers to ensure that all travelers are up to date on immunizations such as MMR, diphtheria/pertussis/tetanus (DTaP, TdaP, Td), and poliovirus vaccine; those who belong to the respective risk groups should receive pneumococcal vaccine.  2. Any traveler who wants to reduce the risk for influenza infection should consider influenza vaccination. Hepatitis A, hepatitis B, and typhoid vaccines should be considered for all travelers to China.  3. Travelers who plan to visit destinations in China outside Beijing should consider additional precautions. These include a pre-exposure rabies vaccine and JE vaccine for longer stays in rural areas, as well as malaria prophylaxis for the infrequent traveler visiting malariaendemic regions. Travelers with pre-existing conditions, such as asthma, may especially need to visit their health care providers for pre-travel advice. | Selected data collected for travelers to China from 1998 through November 2007 by the GeoSentinel Surveillance Network were used to provide an evidence base for prioritizing recommendations for Olympic and other future travelers to China. GeoSentinel Sites are specialized travel or tropical medicine clinics that contribute point of care, clinician-based sentinel surveillance data (diagnosis, travel itinerary, demographics) on all ill travelers that present to the site. To be included in the database, patients must have crossed an international border within 10 years before presentation and have sought medical advice for a presumed travel-related illness. | 134 |
| 2008 | Austria | The 2008 European Football Championship Switzerland, Austria | Measles | Respiratory tract | / | / | The Swiss federal offce of public health urges unvaccinated Swiss people born after 1963 to receive two doses of an MMR vaccine and invites foreign visitors to be vaccinated before travelling in Switzerland. This recommendation is particularly important for people intending to visit large events such as the European football championship (EURO 2008) in June. | About half of the cases (693 or 49%) have been confrmed, either by detecting measles-IgM antibody or the measles virus RNA by RTPCR (555 or 40%), or by an epidemiological link with a laboratoryconfrmed case (138 or 10%). | 135 |
| 2009-2017 | Global | 2009-2017 international athletics championships | Multi-infectious disease | Respiratory tract/Gastrointestinal | Infection (30.8%) was the most common cause of illness, with upper respiratory tract (30%) and gastrointestinal infections (20%) being most frequent. | / | Illness prevention strategies during athletics championships should focus on infectious disease control, and take into account championship type (indoor or outdoor) and discipline category (endurance or explosive). Prevention measures for explosive disciplines should focus on upper respiratory tract infections and pre-existing pathologies. Suggestiions including: (1) Educating athletes and their entourage on illness prevention measures. (2) Maintaining hydration and paying attention to water quality. (3) Eating only ‘safe’ food. (4) Regular hand washing/use of alcohol gel. (5) Paying attention to sleep quality and quantity. (6) Decreasing contact with people outside the team. (7) Being aware of changes in temperature and climatic conditions (including differences between outside and inside). (8) Screening tests for airway problems. | / | 136 |
| (1)2014 (2)2015 (3)2015 | (1)Gaborone city, Botswana; (2)Equatorial Guinea; (3)Republic of Congo | (1) The second African Youth Games (2) The 2015 Africa Cup of Nations (3) XI edition of the All-Africa Games | Multi-infectious disease (during the Ebola outbreak) | Gastrointestinal/Blood-borne | All-Africa Games, Republic of Congo, 2015: 731 cases reported from the sports sites during the events, trauma accounted for 43%, followed by malaria at 27% and respiratory tract infections at 15%. No significant threat to public health was detected during the event. | 1.Mass gatherings at sporting events, or religious pilgrimages, attract millions of international and national hostcountry travellers. 2.Three major sports events were held in Africa during different phases of the Ebola virus outbreak, with participation by sportsmen and women and supporters from a broad range of African countries, including Liberia, Sierra Leone, and Guinea, the three most affected countries. | 1.African Youth Games, Botswana, 2014:  (1) An isolation facility was established in an existing health centre outside of the major hospitals. (2) Extensive staff training included the use of personal protective equipment (PPE) and infection control practices, as well as simulation exercises.  (3) Since Botswana did not have laboratory capacity for VHF and other specialized testing, arrangements were made for testing to be conducted in the biosafety level 4 (BSL4) laboratory and reference laboratories at the National Institute for Communicable Diseases in South Africa.  (4) The public health and hospital laboratories in Gaborone were able to test for malaria and meningitis and common pathogens.  (5)Training sessions in the recognition and management of a range of communicable diseases were held for medical personnel 2. Pre-travel vaccination for vaccine preventable diseases, such as measles, meningococcal meningitis, pneumococcal sepsis, influenza, mumps, and hepatitis A. For communicable diseases that do not have vaccines available, a high state of public health alert, with public health teams on standby coupled to educating the attendees and local population, can go a long way in improving their prevention and detection. | African Youth Games, Botswana, 2014: Both a syndromic approach and laboratory confirmation to identify participants with an acute febrile illness were used. A daily analysis attempted to establish trends. An emergency 24h reporting system was established for persons with suspected meningitis or VHF, and for any outbreaks. | 137 |
| 2014 | Brazil | The 2014 FIFA World Cup Brazil | Multi-infectious disease | Vector/Respiratory tract/Gastrointestinal | / | / | / | 1. Participatory surveillance is based on crowdsourcing methods that collect information from society and then return the collective knowledge gained from that information back to society. 2. Users from anywhere in the world were able to download the Healthy Cup app and record their health condition, reporting whether they were good, very good, ill, or very ill. For users that reported being ill or very ill, a screen with a list of 10 symptoms was displayed. Participatory surveillance allows for the real-time identification of aggregates of symptoms that indicate possible cases of infectious diseases. 3. Participatory surveillance through community engagement is an innovative way to conduct epidemiological surveillance. Compared to traditional epidemiological surveillance, advantages include lower costs of data acquisition, timeliness of information collected and shared, platform scalability, and capacity for integration between the population being served and public health services. | 138 |
| 2016 | Brazil | The 2016 Rio Olympics  Rio, Brazil | Zika | Vector | Until November 2016, there are still no reports on whether the Zika virus was spread from Rio to any other countries. Indeed, all countries launched several methods of disease control among returning travelers from the Games. | The practitioner might overlook other events while focusing only on mass gathering events. Emerging infections in new settings are sometimes carried by travelers from non-endemic areas. | / | / | 139 |
| 2016 | Brazil | The 2016 Rio Olympics  Rio, Brazil | Zika | Vector | / | 1. Mega sporting and mass events put people at risk of acquiring infectious diseases. | 1. Promoting protection strategies and dissemination of accurate information.  2. Strategies such as air conditioning in the Olympic Village bedrooms, use of insect repellents, protective clothing and insecticide-treated mosquito nets should be used to ensure that athletes optimize their performance and experience. | Previous study proposed a mathematical model to calculate the risk of dengue acquisition by foreign tourists at the Olympic Games in Rio 2016, and the incidence of symptomatic and asymptomatic cases among tourists would be 5.75 and 51.5 per 100,000 individuals, respectively. | 140 |
| 2015 | Canada | The 2015 Pan//Parapan American Games Toronto, Canada | Multi-infectious disease | Vector/Respiratory tract/Gastrointestinal/Blood-borne/Water-contact | No major public health incidents occurred that were associated with or a result of hosting the Games. There were two cases of reportable infectious diseases associated with the Games, and 18 public health investigations involving Games-accredited individuals (six related to vaccine-preventable diseases and 12 related to gastrointestinal illnesses or food/water safety violations). | 1. Infectious diseases can be imported from visitors’ countries of origin; 2. Susceptible visitors may be exposed to infectious diseases endemic to the host country; 3. Living arrangements are communal and events can be crowded; 4. The strain on the health care system may cause delayed responses. | New, mobile or temporary food vendors and sanitation facilities are brought in; | (1) Event surveillance: ①Public health coordinator (provincial/Games): A public health professional who was embedded in the polyclinic in the athletes’ village. ②Surveillance teleconferences (all partners): Held on those days that public health surveillance reports for P/PAG were produced. (2) Reportable infectious diseases: iPHIS (provincial): Ontario’s infectious disease surveillance and reporting system. (3) Laboratory: PHOL (provincial): Repository of all submissions to Public Health Ontario for laboratory testing and confirmation (excludes testing by hospitals and community laboratories). (4) Syndromic: ①Gold Medal System (provincial/Games): System included records of medical encounters with accredited individuals (including athletes, coaches, and officials) when they accessed medical services provided by TO2015. ②Telehealth Ontario (provincial): A free, confidential telephone service for Ontario’s general public to seek health advice or information. ③ACES (local/provincial analysis by ACES team): System captures data on chief complaints from emergency department visits and all hospital admissions, covering 53 reporting hospitals across 10 local public health units in the Games geographical area (5) Situational: PHIMS (local/provincial analysis by the ACES team): Online platform displays real-time environmental data (e.g. air quality markers, storm events and temperature stress) along with ACES, demographic, and social deprivation indices on a geographical interface. (6) International: GPHIN (provincial/federal): Program that uses an automated web-based system to scan newspapers and other communications worldwide for potential indicators of outbreaks. These are then analyzed and rapidly assessed by a multilingual, multidisciplinary team. | 141 |
| 2016 | Brazil | The 2016 Rio Olympics  Rio, Brazil | Multi-infectious disease | Vector/Respiratory tract/Gastrointestinal | The platform Guardians of Health had 7848 users who generated 12,746 reports about their health status. Among these reports, the following were identified: 161 users with diarrheal syndrome, 68 users with respiratory syndrome, and 145 users with rash syndrome. | / | / | A mobile app, a web app, and a dashboard platform (Guardiões da Saúde (Guardians of Health in Portuguese)) were developed, which were used in a project conducted during the 2016 Olympic and Paralympic Games in Rio de Janeiro, Brazil, and officially used by the Brazilian Ministry of Health for the monitoring of outbreaks and epidemics. Based on syndromic signals, the information subsidy for decision making by policy makers and health managers. This type of information source can be used as an early route to understand the epidemiological scenario. | 142 |
| 2020 | Yas Island, Abu Dhabi | Ultimate Fighting Championship (UFC) | COVID-19 | Respiratory tract | A total of 18 530 samples were collected by 97 nurses and 18 706 tests were conducted (due to retesting of borderline results). During the 4 weeks, 17 tests returned positive results, but on resampling and retesting two times, all of these were found to be false-positive results. | / | 1. An area of 11.8 km2 on Yas Island was identified to be turned into a virus free ‘safe zone’. Two weeks before the launch of UFC ‘Fight Island’, the zone was secured. 2. International delegates were quarantined in airport hotels for 48 hours prior to their departure and could only board their planes on receipt of a negative result. All passengers were required to wear personal protective equipment (PPE) and follow normal hygiene measures with social distancing, while all Etihad crew and airport staff were quarantined and tested regularly for 2 weeks prior to the flights (as were all bus drivers). One of the Abu Dhabi Airport terminals was made available for the exclusive use of the international delegates. 3. All international delegates within the safe zone were subjected to 48 hours quarantine on arrival (with two PCR tests conducted 24 hours apart during this time).Ongoing education on hygiene was provided and strict sanitization measures social distancing and PPE wearing were enforced. Participants were subjected to daily temperature and symptom checks and movement into and out of the zone was stringently restricted. Thermal cameras situated throughout the zone continuously monitored peoples’ temperatures. Athletes were generally kept away from other participants where possible and had their own private, exclusive gym and training area adjacent to each of their hotel rooms. There was additional focus on sanitising the arena before, during and after fights. Everyone within the safe zone was tested two times per week, including 24 hours before an event. Separate isolation rooms were established and anyone testing positive or leaving and re-entering the zone was placed in these areas. 48 hours quarantine and two negative PCR results allowed re-entry into the zone. Fighters requiring medical attention outside of the zone were taken to ‘COVID-free’ hospitals, full PPE was worn and contact with anyone outside limited as much as possible. | / | 143 |
| 2020 | Japan | The 2020 Olympics Tokyo, Japan | Hepatitis A | Gastrointestinal | / | / | To prevent a further hepatitis A outbreak at the time of the Tokyo Olympic Games in 2020, hepatitis A vaccination among susceptible populations such as MSM should be promoted vigorously. In the meantime, awareness and education of the general public as well as of healthcare professionals is warranted. | / | 144 |
| 2018 | Korea | The 2018 Winter Olympics Pyeongchang, Korea | Multi-infectious disease | Vector/Respiratory tract/Gastrointestinal/Blood-borne/Water-contact | 1. The respiratory system was the system at the highest risk for illness at these Games (incidence rate of 4.1 [95% CI 2.9 to 5.9]).  2. The overall illness burden at the Pyeongchang 2018 Games was 6.8 days lost per 1000 athlete days (95%CI 3.4 to 13.5), and the system with the highest illness burden was the respiratory system (illness burden of 1.4 [95% CI 0.6 to 3.0]). | The first important finding of this study was that the relatively new sport of Para snowboard was identified as a high risk for all illnesses, and specifically illnesses in the skin and subcutaneous system. | / | / | 145 |
| 2018 | Britain | The 2018 Commonwealth Games Triathlon Broadwater, Britain | Escherichia coli | Gastrointestinal | / | / | / | 1. A number of Bayesian Networks were developed in order to nowcast and forecast, up to 4 days ahead and in diﬀerent locations, the likelihood of water quality within the 2018 Commonwealth Games Triathlon swim course exceeding the critical limits for Enterococci and Escherichia coli. The model relied mainly on rainfall-related predictors. The model was deployed in real-time during the games and predicted accurately. The Bayesian Network modelling framework proved to be a cost-eﬀective, less time-consuming alternative to numerical modelling (such as hydrodynamic models) or traditional statistical models. | 146 |
| 2016 | Brazil | The 2016 Rio Olympics  Rio, Brazil | Zika | Vector | Of 92 participants, one individual showed high IgM titers before (OD = 0.687 at 450 nm) and after travel to Brazil (OD = 1.90) compared to 0.062 for negative control and 0.924 for the positive control. The samples had high IgM titers to WNV, TBEV, and YFV. | / | As ZIKV infection is often asymptomatic in healthy adults, it is important to follow up with specific laboratory testing to determine true infection rates, not just apparent infections. |  | 147 |
| / | Around the world | e.g., Olympics and FIFA World Cup | Multi-infectious disease | Vector/Respiratory tract/Gastrointestinal/Blood-borne/Water-contact | / | Close contact of the attendees in confined and crowded spaces, demographics and disease exposure history of the participants, their mobility patterns, the event setting, climate conditions, as well as the incoming and outgoing travel patterns of international participants attending these events. | / | 1.Data used in the health surveillance systems:  (1) Related health data like clinical data from health providers (hospitals, physicians, and laboratories), drug sales from pharmacies, outbreak reports, population demographic data, and emergency and urgent care data.  (2) Web searches, social media posts, environmental data, and travel patterns. However, the existing noise in Internet data presents a significant obstacle. 2. HealthMap was used to provide real-time analysis and detect any serious health threats In the Vancouver 2010 Olympic Winter Games.  3. Models: SIR (SusceptibleInfected-Recovered) compartmental model, agent-based modeling (ABM). | 148 |
| 2016 | Brazil | The 2016 Rio Olympics  Rio, Brazil | Multi-infectious disease | Respiratory tract/Gastrointestinal | 1. Of the illnesses, 47% affected the respiratory system and 21% the gastrointestinal system. The anticipated problem of infections in the Rio Olympic Games did not materialise, as the proportion of athletes with infectious diseases mirrored that of recent Olympic Games (3%). 2. While the majority of illnesses in Rio (56%) were caused by an infection, the proportion of athletes contracting an infection (3%) was identical to London 2012 (3%) and lower than Sochi 2014 (5%). Similarly, the incidence of digestive system illness (1%) was identical to London 2012 (also 1%). In terms of the Zika virus, no cases were reported among either athletes or the general population during the Olympic Games in Rio. | / | / | We recorded the daily incidence of athlete injuries and illnesses through the reporting of all National Olympic Committee (NOC) medical teams and in the polyclinic and medical venues by the Rio 2016 medical staff. | 149 |
| 2016 | Brazil | The 2016 Rio Olympics  Rio, Brazil | Zika | Vector | For 4 persons, test results for Zika virus IgG was positive; IgM and neutralization testing yielded negative results. The 4 Zika virus IgG–positive participants had received previous yellow fever vaccination and were asymptomatic. One sample showed Zika virus IgM in the absence of specific IgG; the results were confirmed as false positive. | / | / | To evaluate the risk for the Spanish Olympic Team acquiring Zika virus, data were collected in 6 diﬀerent recruiting Tropical Medicine Units in cities in Spain (Barcelona, Madrid, Sevilla, San Sebastian, Las Palmas de Gran Canaria, and Almeria). | 150 |
| 2018 | Australia | The 2018 Commonwealth Games Triathlon Broadwater, Australia (GC2018) | Zika | Vector | / | The primary vector of the virus, Aedes mosquito, is currently present in Australia. Aedes aegypti is common in the Northern and Fart North coastal areas of Queensland. It also occurred in South East Queensland in the past. | Control of Aedes mosquito population by targeting them directly or indirectly, effective use of condom in preventing sexual transmission, and early detection and containment of any imported cases will be essential for the prevention of local transmission of Zika during Commonwealth Games 2018. | Surveillance of adult mosquitoes using traps and aspirators provide more direct evidence on the disease transmission risk. | 151 |
| 2008 | China | The 2008 Olympics Beijing, China | Multi-infectious disease | Vector/Respiratory tract/Gastrointestinal/Blood-borne/Water-contact | / | 1. Rapid increase in population density.  2. Summer is also the peak period for heightened vector activities such as those of rats, mosquitoes, flies, cockroaches, etc.  3. Frequent travel of people across different provinces or countries. | / | 1.Beijing Olympic Games Infectious Disease Surveillance System (BOG-IDSS) In general, BOG-IDSS consists of two major components: disease surveillance targeted at notifiable infectious diseases as defined by the State, and syndromic surveillance targeting health-related symptoms. Specifically, four subsystems for infectious disease surveillance were integrated in BOG-IDSS, including three routine surveillance and one newly established system: the Syndromic Surveillance System for Beijing Olympic Games (SSSBOG). 2. The data source of BOD-IDSS mainly included CIDARS, SSSBOG, Early Warning Surveillance System for Influenza of Beijing and Early Warning Surveillance System in Intestinal Outpatients of Beijing. | 152 |
| 2015 | United States | Super Bowl XLIX (at the University of Phoenix stadium in Glendale, Arizona) | Multi-infectious disease | Vector/Respiratory tract/Gastrointestinal/Blood-borne/Water-contact | During the Super Bowl events, there were 9 open outbreak investigations (4 influenza investigations, 3 pertussis investigations, 1 respiratory, and 1 GI). 2.For this event, neurological symptoms were also the most commonly reported (n = 44), followed by respiratory (n = 10) and gastrointestinal (n = 7). | / | / | Surveillance strategies implemented from January 22 to February 6, 2015, included enhanced surveillance alerts; animal disease surveillance; review of NFL clinic visits; syndromic surveillance for emergency room visits, urgent care facilities, and hotels; real-time onsite syndromic surveillance; all-hazards mortality surveillance; emergency medical services surveillance, review of poison control center reports; media surveillance; and aberration detection algorithms for notifiable diseases. | 153 |
| 2016 | Brazil | The 2016 Rio Olympics  Rio, Brazil | Dengue | Vector | / | / | / | A mathematical model to calculate the risk of developing dengue for foreign tourists attending the Olympic Games in Rio de Janeiro in 2016 is proposed. A system of differential equation models the spread of dengue amongst the resident population and a stochastic approximation is used to assess the risk to tourists. Historical reported dengue time series in Rio de Janeiro for the years 2000-2015 is used to find out the time dependent force of infection, which is then used to estimate the potential risks to a large tourist cohort. | 154 |
| 2014 | Scotland | The 2014 XX Commonwealth Games Glasgow, Scotland | Sexually Transmitted Infections | Blood-borne | There was a significant decrease in core sexual health attendances, total acute STIs and emergency hormonal contraception prescriptions. There was no change in post exposure prophylaxis after sexual exposure (PEPSE) prescriptions or the number of reported sexual assaults throughout the city. | Large mass-gathering sporting events adversely affect the sexual and reproductive health (SRH) outcomes of the local and visiting populations. | / | / | 155 |
| 2016 | Brazil | The 2016 Rio Olympics  Rio, Brazil | Multi-infectious disease | Vector | / | / | 1. The more effective and long lasting a repellent is, the higher the level of protection. Complete protection time on human skin DEET repellents had a better repellency against Aedes over a longer time period.  2. Vitamin B 1(thiamine) has often been reputed to reduce human attractiveness for mosquitoes and thereby exert a protective effect. | / | 156 |
| 2014 | Brazil | The 2014 FIFA World Cup Brazil | Dengue | Vector | / | / | / | The data were obtained from the Brazilian notification database SINAN. Time series analysis via box-plots, combined with the information given by the precipitation data in Brazil, from the Instituto Nacional de Meteorologia (INMET) were used. And the seasonality of the disease plays a major role in dengue transmission. | 157 |
| 2012 | Finland | The 2012 European Athletics (EA) Championships Helsinki, Finland | Multi-infectious disease | Respiratory tract/Gastrointestinal | The most common diagnoses were upper respiratory tract infection (33.3%) and gastroenteritis/diarrhea (25.9%). | / | Previously published recommendations should be followed to prevent these illnesses: educate athletes and their entourage on infectious disease and dehydration prevention strategies, drink regularly and bottled water only, eat safe food and employ regular hand washing with alcohol gel, decrease contact with people outside the team, and be careful with temperature and climatic conditions changes. | Prospective recording of newly occurred injuries and illnesses. | 158 |
| 2014 | Brazil | The 2014 FIFA World Cup Brazil | Dengue | Vector | / | / | / | 1. The dengue risk map (http:\\go.nature. com/8g1io5) appears to be from a previously published global dengue risk assessment, but zoomed into the Brazilian territory.  2. The model was built using precipitation, temperature, vegetation/moisture, urbanisation, accessibility, and poverty as spatial covariates.  3. The nationwide risk maps were produced by smoothing mean dengue incidence rates during February and June, respectively, between 2001 and 2012.  4. A recent study produced a dengue early warning three months ahead of the 2014 World Cup, driven by seasonal climate forecasts and the epidemiological situation at the time of forecast, with a risk map and uncertainty estimates for the 553 microregions of Brazil. | 159 |
| 2014 | Brazil | the 2014 FIFA World Cup and the 2016 Olympic and Paralympic Games | Multi-infectious disease | Vector/Respiratory tract/Gastrointestinal/Blood-borne | The most common travel-related illnesses were dermatologic conditions (40%), diarrheal syndromes (25%), and febrile systemic illness (19%). Dengue and malaria, predominantly Plasmodium vivax, were the most frequently identified specific causes of fever and the most common reasons for hospitalization after travel. Dengue fever diagnoses displayed marked seasonality. Among the 28 ill returned travelers with human immunodeficiency virus (HIV) infection, 11 had newly diagnosed asymptomatic infection and 9 had acute symptomatic HIV. | / | 1. Skin problems: prevention is by avoidance of skin contact with soil/sand. 2. Myiasis and tungiasis: prevention is by use of insect repellents and/or wearing long sleeves and trousers during the day. Use of closed-toed footwear and avoidance of skin contact with soil. 3. Cutaneous leishmaniasis: Travelers should be advised to reduce bites from sandflies, whose bite transmits infection, by using insect repellent and/or wearing long-sleeved shirts and trousers during evening hours in endemic regions. acute and chronic diarrheal syndromes-food and water precautions are recommended. 4. Dengue: The primary way to avoid infection is to prevent mosquito bites with repellent on exposed skin. Only travelers to certain areas of Brazil will be at risk for malaria, but travelers to all areas will be at risk for dengue. 5. Malaria: Travelers who will visit malaria-endemic areas can be protected with chemoprophylaxis  6. Yellow fever: Vaccination is recommended for several cities hosting World Cup events, including Belo Horizonte, Brasilia, and Manaus 7. STIs: Avoid unsafe sexual practices and exposure to potentially contaminated needles and blood.  8. Measles, rubella, mumps,pertussis: travelers should be up to date with these vaccinations. 9. Influenza: Influenza vaccine. | The GeoSentinel Surveillance Network (www.istm.org/ geosentinel) is an international network of specialized travel and tropical medicine clinics located on 6 continents. All sites collect data by using a standard reporting form on ill travelers seen during or after international travel. Anonymized data on demographics, travel history, reason for travel, pretravel advice, hospitalization, major clinical symptoms, and final diagnoses assigned by the GeoSentinel site clinician are electronically entered into a central database. | 160 |
| 2014 | Brazil | The 2014 FIFA World Cup Brazil | Dengue | Vector | / | / | The risk of dengue among tourists during the World Cup is expected to be small. Quantitative risk estimates by different groups and methodologies should be made routinely for mass gathering events. | We used dengue incidence rates reported by each host city during previous years (2001– 2013) to estimate the risk of dengue during the World Cup for tourists and teams. Two statistical models were used: a percentile rank (PR) and an Empirical Bayes (EB) model. | 161 |
| 2014 | Brazil | The 2014 FIFA World Cup Brazil | Multi-infectious disease | Vector/Respiratory tract/Gastrointestinal/Blood-borne/Water-contact | / | / | 1. All travelers should be up-to-date on their routine vaccines, including hepatitis A, influenza, measles, mumps and rubella, but also tetanus, diphtheria, pertussis, pneumococcal and varicella if possible, as not all persons would be candidates for these last two vaccines in many countries. 2. Travelers visiting high risk areas (e.g. Amazon areas) should be assessed regard the potential need for chemoprophylaxis. If indicated, atovaquoneeproguanil, doxycycline or mefloquine are recommended.  3. Yellow fever: This is not recommended for travelers visiting cities such as Rı ´o de Janeiro, Sao Paulo, Salvador, Recife and Fortaleza. Unvaccinated individuals traveling to areas where vaccination is recommended should be vaccinated at least 10 days prior to travel. Public education is needed about the risk of disease and indications for vaccination, including contraindications and precautions for persons who might be at increased risk of severe adverse events. 4. Preventive measures include reducing exposure to sand flies by using personal protective measures. Travelers should be advised to: avoid outdoor activities, especially from dusk to dawn, when sand flies generally are most active; wear protective clothing and apply insect repellent to exposed skin and under the edges of clothing, such as sleeves and pant legs, according to the manufacturer’s instructions; sleep in air-conditioned or well-screened areas; spraying the quarters with insecticide might provide some protection. Fans or ventilators might inhibit the movement of sand flies.  5. Condom use should be recommended for travelers with the potential to engage in sexual relationships when visiting Brazil. | / | 162 |
| 2018 | Korea | The 2018 Winter Olympics Pyeongchang, Korea | Influenza | Respiratory tract | / | / | / | 1. A multi-city transmission model was used to predict the infection route during 2018 Winter Olympics in Korea based on the pre-existing SIR model. Various types of transportation system such as a train, a car, a bus, and an airplane for the interpersonal contact in both inter-and intra-city are considered. Simulation is performed with assumptions and scenarios based on realistic factors including demographic, transportation and diseases data in Korea. | 163 |
| 2012 | Britain | The 2012 Olympics London, Britain | Multi-infectious disease | Vector/Respiratory tract/Gastrointestinal/Blood-borne/Water-contact | 49 separate incidents were identified. Of these, 17 were related to gastrointestinal infections such as salmonellosis, cholera and Escherichia coli infection, 12 to childhood infections such as hand, foot and mouth disease, pertussis and measles, seven to influenza, seven to zoonoses such as anthrax and those due to infection with West Nile virus, hantavirus and Hendra virus, three to viral haemorrhagic fevers such as Lassa and Ebola and a further three to other infections. In terms of the geographical location of these incidents, 18 were reported in Europe, 10 in North America, eight in Asia, seven in Africa, four in Oceania and two in South and Central America. Of the 17 gastrointestinal disease incidents, nine had specific foods implicated as the source and the international team followed up six of these with the UK Food Standards Agency. | / | Countries hosting large sporting events in the future will need to consider to what degree they will need to supplement alerting systems such as these with their own, and/or collaborative, EI processes, when determining how to allocate resources to international surveillance among the wide range of public health responses required for such events. | 1. International surveillance for London 2012 was based on an enhanced ‘business as usual’ model and was part of wider surveillance activity that has been previously described. The international team developed an enhancement of their normal processes that was extensively tested and refined to maximise sensitivity and specificity of identification of ID incidents relevant for the Games, and to use resources efficiently. 2.Resources to support the international infectious disease incident surveillance: (1)Epidemic intelligence: Event-based surveillance systems (ECDC); Epidemic Intelligence Information System, EPIS (ECDC); Routine fortnightly surveillance of influenza in the southern hemisphere (ECDC); Weekly surveillance of measles outbreaks worldwide (ECDC); Criteria for London 2012 relevance (HPA, TMHS). (2)Databases: Threat tracking tool (ECDC); HPA Olympic international surveillance database (HPA, TMHS). (3)Communication: Extranet (ECDC); Shared drive (HPS, Colindale). (4)Protocols: Standard operating procedures (HPA, TMHS, ECDC); International risk assessment teleconference resources (HPA, TMHS). (5)Risk assessment support tools: Epidemiological profiles (HPA, TMHS and EIZ); Travel patterns (HPA, TMHS); Risk definitions (HPA, EIZ). | 164 |
| 2012 | Britain | The 2012 Olympics London, Britain | respiratory illness, influenza-like illness (ILI), difficulty breathing/ wheeze/asthma, gastroenteritis, vomiting, diarrhoea, myocardial infarction (MI) and heatstroke. | Respiratory tract/Gastrointestinal | / | / | / | The Health Protection Agency has developed a new syndromic surveillance system that monitors daily general practitioner out-of-hours and unscheduled care attendances. This new national system will fill a gap identified in the existing general practice-based syndromic surveillance systems by providing surveillance capability of general practice activity during evenings/nights, over weekends and public holidays. | 165 |
| 2012 | Britain | The 2012 Olympics London, Britain | Multi-infectious disease | Respiratory tract/Gastrointestinal | / | / | With this in mind, several practical lifestyle, nutritional and hygiene strategies to minimize infection risk in athletes have been suggested, including avoiding people with infectious illness where possible, practicing good personal hygiene (washing hands, brushing teeth regularly), not sharing drinking bottles or cutlery, getting adequate sleep, managing psychological stress, maintaining energy and fluid balance, and considering vaccinations particularly against seasonal in fluenza. These should become part of an athlete’s normal routine. | / | 166 |
| 2011 | Korea | The 2011 13th International Association of Athletics Federations World Championships Daegu, Korea | Multi-infectious disease | Respiratory tract/Gastrointestinal | A total of 126 illnesses were reported, signifying an incidence of 68.1 per 1000 registered athletes. Upper respiratory tract infection was the most common reported diagnosis (18%), followed by exercise-induced dehydration (12%), and gastroenteritis/diarrhoea (10%). The highest incidences of injuries were found in combined events and middle and long-distance events, and of illness in race walking events. | / | 1. Educate of athletes and their entourage on infectious disease and dehydration prevention strategies.  2. Athletes should be advised to seek shade and to hydrate regularly, to drink only bottled water, to eat safe food and to employ regular hand washing with alcohol gel.  3. The use of plastic to cover carpeted hotel rooms, and special indoor air cleaning systems, along with discouraging hand shaking and close contact with people outside the team, including fans and the media, should also decrease illness transmission.  4.Screening tests on airway problems and adapted strategies for athletes with a heavy competition load should be offered to all athletes at risk. 5. Appropriate event scheduling and heat acclimatisation. |  | 167 |
| 2010 | Canada | The 2010 Winter Olympics Vancouver, Canada | Sexually Transmitted Infections | Blood-borne | There were no significantly increased odds in perceived reports of new (0.999), youth (0.536) or trafficked SWs (zero reports) in the Olympic period. | / | 1. To reduce potential adverse impacts from displacement of local sex work markets, evidence-based public health strategies should be adopted for host cities of future events. 2.The removal of criminal sanctions targeting sex work and the piloting and rigorous evaluation of safer indoor sex work spaces should be considered. | / | 168 |
| 2010 | Canada | The 2010 Winter Olympics Vancouver, Canada | Sexually Transmitted Infections | Blood-borne | / | / | Safer sex work spaces such as indoor brothels and policy reforms should be considered both in Canada and by other host countries of large-scale events to reduce the vulnerability of SWs to HIV from displacement and disruption. | / | 169 |
| 1999 | United States | An 1999 International Youth Sporting Event in the United States | Measles | Respiratory tract | The index case occurred in a child from Japan aged 12 years. Contact tracing among 1250 persons in 8 states identified 7 measles cases; 5 (71%) cases occurred among persons without documented measles vaccination. Epidemiologic and laboratory investigation supported a single chain of transmission, linking the outbreak to contemporaneous measles virus genotype D5 transmission in Japan. | Participants in international events, international travelers, and persons with routine exposure to such travelers might be at greater risk of measles. | 1. Isolation of patients and administration of postexposure prophylaxis to contacts who lacked evidence of presumptive measles immunity through documented vaccination, laboratory evidence of immunity, history of physician-diagnosed measles, or birth before 1957. 2. High measles, mumps, and rubella vaccine coverage rates should be maintained. 3. The outbreak highlights the importance of maintaining and having readily available immunization records for US residents and travelers. 4. Encourage participants and attendees to be fully vaccinated against measles and should enhance efforts to improve immunization documentation among all players, event staff, and coaches. | 1. Public health authorities initiated a multistate investigation because of the patient’s travel history and international event participation. 2. Vaccination status was sought from sources including parents, immunization cards, schools, and healthcare providers.  3. To identify aircraft-associated exposures, flight manifests were requested and contact information was obtained for passengers seated within 1 row of the index patient on the same side of an airplane aisle. 4. Laboratory Investigation: Nasopharyngeal, oropharyngeal, or urine specimens were collected from persons with suspected measles for RT-PCR testing and viral culture. | 170 |
| 2010 | South Africa | The 2010 FIFA World Cup South Africa | Meningococcal | Respiratory tract | / | / | Recommendation that the administration of a conjugate quadrivalent meningococcal vaccine should be considered by travel health practitioners and others who will be involved in identifying the travel health risks of those who will be attending or playing in the 2010 FIFA World Cup South Africa. | / | 171 |
| 2010 | South Africa | The 2010 FIFA World Cup South Africa | Sexually Transmitted Infections | Blood-borne | / | / | 1.Distribution of free male and female condoms, and raising awareness on safer sex and sex worker rights during World Cup games are critical.  2. Following the World Cup, pressure should be increased on the South African Law Reform Commission to recommend full decriminalisation of sex work in South African law. | / | 172 |
| (1) 1960 (2) 1964 (3) 1968 (4) 1972 | (1) Rome (2) Tokyo (3) Winnipeg (4) Mexico (5) Sapporo | Olympics (Rome, 1960; Tokyo, 1964; Winnipeg, 1968;Mexico, 1968; Sapporo, 1972) | Multi-infectious disease | Vector/Respiratory tract/Gastrointestinal/Blood-borne/Water-contact | / | / | 1. The planning begins with the gathering of information about the host city and its environs, including climate and temperature fluctuations (especially important for those outdoor events which may go all day and into the evening and night, as well as those events which take place at night only); altitude; water for drinking and for swimming; ice supply; milk supply; food; restaurants; availability of towels, soap, laundry facilities, and beds for those athletes who are more than 188 cm (6 feet 2 inches) tall; and diseases of both humans and animals which are endemic. 2. The Medical and Training Services Committee is responsible for choosing the personnel to staff the medical team, and to assist at the site of the Games, and most of all, to plan for the proper operation of the medical and training services. The number of medical personnel (including nurses and trainers) is limited by the regulations of the International Olympic Committee and the organizing committee of the host city (nation). | / | 173 |
| 1998 | Malaysia | The 1998 Commonwealth Games Kuala Lumpur, Malaysia | Tetanus/Diphtheria/Measles/Mumps/Polimyelitis/Rubella/Hepatitis A/Hepatitis B/influenza/Malaria/Typhoid fever/Japanese encephalitis/Cholera/Rabies/Meningococcus/Yellow fever | Respiratory tract/Blood-borne/Vector/Gastrointestinal | / | 1. Air travel, cramped living conditions, and high training loads. 2.Tropical mosquitoes can spread malaria, such as dengue fever, and Japanese encephalitis. 3. The hot, humid condition of Malaysia provide excellent growing conditions for fungal infections of the skin and nails, which are particularly likely to affect the groin and toes. 4. Adjustment of circadian rhythms takes longer with increasing age, the mental stress associated with travel, and alcohol consumption. 5. During a 2-week stay in such an area, between 20% and 50% of visitors develop a diarrheal illness. | 1. Recommended immunization and prophylactic measures for the travelling athlete, including basic, essential and recommended vaccines. 2. Antimalarial chemoprophylaxis may be required for those training and residing outside Kuala Lumpur. 3. Athletes are encouraged to sleep well the night before departure, synchronize their watches with the current time at destination on boarding, and minimize the time spent in transit lounges by appropriate flight scheduling. 4. To minimize the stress of jet travel, athletes should arrive early at the airport, arrange appropriate in-flight meals, wear loose comfortable clothing, and carry personal material for relaxation. And be prepared for delays and stressful events such as lost luggage. 5. Pay attention to food hygiene and advice to athletes including careful hand washing before meals, ensuring that all food has been freshly cooked, and water boiled for at leat 10 minutes. 6. Protection from mosquitoes is encouraged with the use of insect repellents on the skin, sleeping in screened air-conditioned rooms or with mosquito netting around beds, mosquito coils, and the wearing of light-colored, long-sleeved shirts and long trousers. 7. Personal hygiene, keeping areas at the risk clean and dry as far as possible, and frequent washing of kit greatly reduces the risk. | / | 174 |
| 1998 | United States | The 1998 Triathlons Wisconsin and Illinois, United States | Leptospirosis | Water-contact | 834 (95%) of 876 participants in the Springfield triathlon were interviewed; 98 athletes met the definition for a suspected case (attack rate, 12%). Of the 98 athletes who met the definition for a suspected case of leptospirosis, 32 (33%) submitted 1 serum sample and 53 (54%) submitted 2 serum samples . Of these 85 athletes, 52 (61%) had signs, symptoms, and test results that met the definition of a laboratory-confirmed case. | Heavy rains that preceded the triathlon are likely to have increased leptospiral contamination of Lake Springfield. Among athletes, ingestion of 1 or more swallows of lake water was a predominant risk factor for illness. | Health care providers and occupational and recreational users of bodies of freshwater in the United States should be aware of the risk of contracting leptospirosis, particularly after heavy rains. | 1. Serum samples were obtained from 2 of the 3 hospitalized patients and were immediately tested for leptospirosis at the Centers for Disease Control and Prevention. 2. Telephone survey was used to characterize the illness and to identify its risk factors. 3. Because Lake Springfield is heavily used for recreational purposes, surveillance systems were established to identify community residents with leptospirosis. An environmental investigation was undertaken to identify the source for and the extent of leptospiral contamination of Lake Springfield. | 175 |
| 2000 | Australia | The 2000 Olympics Sydney, Australia | Multi-infectious disease | Gastrointestinal/Respiratory tract | / | 1. Most general practitioners reported not being well prepared before the Sydney Games and considered pre-planning training and seminars would have been their preference.They also reported that they were not in a position to become more involved in the provision of medical services for visitors during the Olympic and Paralympic Games. 2. Disease outbreaks are not just an issue for local health authorities, as they are also a concern for travellers returning home from the host country with the possibility of having acquired a communicable disease during their travelling. | 1. Travellers to avail themselves of adequate travel insurance coverage when travelling to the next Olympic and Paralympic Games, in Athens, Greece. 2. Prepare health facilities to cater for the health and travel health needs of all athletes and visitors to the Games. | 1. An Olympic Surveillance System was established for monitoring infectious diseases during the Sydney 2000 Games, with a particular focus on food-borne diseases and diseases spread via the respiratory route.  2. Expand the information sources, use a targeted and systematic screening approach using tailored tools (MediSys), determine validation sources, establish a daily analysis and communication process with regular and specific public health partners and developing specific reports. 3. Information on public health events detected in EU/ EEA countries was validated through routine channels with EU Member States, while those detected outside the EU/EEA (excluding South Africa) were validated through the WHO Regional Office for Europe and the ECDC EI international network. | 176 |
| 2006 | Italy | The Olympic Winter Games in Torino, Piemonte, Italy | acute gastroenteritis, influenza-like illness and measles | Gastrointestinal/Respiratory tract | 1. Only 24 acute viral gastroenteritis, 11 influenza-like illnesses and three cases of illness related to low environmental temperatures were reported. 2.Of the consultations of the GP Sentinel network, 6% were for acute gastroenteritis and 12% for influenza-like illness. Only one consultation for measles was recorded. 3.No cluster of cases was detected by the two public and private microbiology laboratories in Briançon during the period of surveillance. | 1. large numbers of people gathering in the same place, which could increase the risk of disease transmission.  2. possible saturation of healthcare structures. 3. installation of temporary restaurants with potentially precarious hygiene conditions despite reinforcement of hygiene inspetions. 4. Mobility of the population. | / | 1. Epidemiologic surveillance system: The system aimed to complement to the Italian system; surveillance targeted diseases with strong infectious epidemic potential (such as meningitis, legionellosis and gastroenteritis) or events which could represent a specific risk in the area under surveillance (such as very low environmental temperatures or carbon monoxide poisoning). Reinforcement of the carbon monoxide poisoning monitoring was carried out. 2. In the system： (1) Mortality was tracked using by analysing the number and cause of deaths. (2) A sentinel network of general practitioners was set up and reported the frequency of acute gastroenteritis, influenza-like illness and measles.  (3) Medical laboratories provided data about the analyses they undertook.  (4) Hospital emergency department and emergency ambulance service activities were followed up.  (5) Statutory notification diseases and toxic effects of carbon monoxide surveillances were reinforced.  (6) Analysed data were transmitted daily to the health authorities.  (7) A French/English report was sent weekly to all participants. | 177 |
| 2004 | Greece | The 2004 Olympics Athens, Greece | Multi-infectious disease | Gastrointestinal/Respiratory tract/Water-contact | In August, 2003, an outbreak of salmonellosis was linked to a hotel restaurant which accommodated athletes during a test event. | 1. A large number of people were potentially exposed to possible environmental health hazards given the massive food service operations, common and excessively used potable and recreational water supplies, crowded accommodation, and shared sanitary facilities in hotels, Olympic Venues, and cruise ships.  2. Massive food production and consumption, crowded accommodation settlements, common water supplies and heavily overloaded sanitation and sewage systems, thus presenting a substantial smoldering risk for disease transmission. | / | **The integrated environmental health surveillance program for the Athens Olympic Games:** Statistical analysis over time of the standardized inspection results and water quality tests results together with the communicable diseases surveillance findings were used to assess the performance of the environmental health surveillance program. 1.Standardized inspections: (1) A total of 19 forms to register information regarding premises of environmental health interest. (2) A total of 17 standardized scored inspection forms were developed Food premises: Canteens; Hotels; Ships; Swimming pools; Water supply systems; Cooling towers; Decorative fountains; Ice production plants; Areas requiring pest control; Waste management facilities; Public toilets; Sewage treatment units; Bottled water plants; Camps; Seacoast; Marinas. 2.Water quality surveillance program 3.Standardized corrective actions 4.Computerized electronic network 5.Training and Guidelines for professionals and owners 6.Vessel Sanitation | 178 |
| 2010 | South Africa | The 2010 FIFA World Cup South Africa | Not specified | Not specified | / | / | 1. Strengthening infectious disease surveillance, including vigilance with regard to imported cases;  2. Ensuring that food preparation safeguards against contaminants | The implementation of an electronic patient record will be a tremendous legacy, especially for users of the public health sector in South Africa. | 179 |
| 2004 | Greece | The 2004 Olympics Athens, Greece | Respiratory infection; diarrhea; gastroenteritis; meningitis, encephalitis; suspected viral hepatitis; lymphadenitis with fever; sepsis or unexplained shock | Respiratory tract/Gastrointestinal/Blood-borne/Vector/Water-contact | / | / | / | 1. THE 2004 SUMMER OLYMPIC GAMES SYNDROMIC SURVEILLANCE SYSTEM: A drop-in syndromic surveillance system in eight major hospitals and a major healthcare center in the greater Athens area were established by the Hellenic Center for Infectious Diseases Control, in August 2004. All syndromes (i.e., respiratory infection with fever; bloody diarrhea; gastroenteritis, without blood; febrile illness with rash; meningitis, encephalitis, or unexplained acute encephalopathy/delirium; suspected viral hepatitis; botulism-like syndrome; lymphadenitis with fever; sepsis or unexplained shock; and unexplained death with history of fever) presented daily to the emergency department were recorded along with the number of total visits. The project included trained personnel (nurses, physicians, bio-statisticians and epidemiologists) who visited the hospitals, and identified the cases from the visit books. Then the information was entered in a database and data analysis was performed. 2.Outbreak detection algorithms (1) One of them that was developed by Dafni et al. was applied during the 2004 Summer Olympic Games.Using these algorithms public health authorities may have a tool for early detection of unexpected outbreaks of serious threats, like avian influenza, nutrition related diseases, and so on. (2) Based on this algorithm, and on a daily basis, the Public Health authorities were informed about potential threats for the people that were involved in the Olympics, as well as for all people living in the Athens greater area. | 180 |
| 2008 | China | The 2008 Olympics Beijing, China | Malaria/Food and water borne disease/Hepatitis B/Influenza/Japanese B encephalitis/Meningococcus/Measles,mumps,rubella/Polio/Rabies/Tuberculosis/Tetanus/Typhoid fever/Yellow fever | Gastrointestinal/Vector/Water-contact/Blood-borne/Respiratory tract | / | / | 1.Need to be vaccinated for vaccine preventable diseases. 2. AIDS/HIV:all travellers and competitors at the games are recommended to be extremely cautious about any risk of exposure to the disease from accidents or dental surgery, sexual transmission, blood exposure (such as from unsterile needles and syringes) as well as from unscreened blood transfusions. 3.Avian influenza: travellers to affected areas should avoid contact with live animal markets and poultry farms, and any free-ranging or caged poultry. 4.Dengue fever:Protective measures against mosquito bites should be taken. 5.Helminthic diseases: wearing shoes and not walking barefoot outside; washing salads and/or vegetables or thoroughly cooking food. 6.Sexually transmitted infections: Travellers need to be advised about the significant risks associated with unprotected casual sexual relations. | / | 181 |
| 2008 | Austria | The 2008 European Football Championship Switzerland, Austria | Unspecified | Not specified | / | / | Measures planned in Austria for EURO 2008 include: 1. Training: A workshop dealing with mass gatherings will be run by the ECDC and the RKI for the Austrian health authorities and the food safety sector. 2. Increased awareness among the reporting doctors: Doctors in hospitals and practices will be asked to report infectious diseases or possible outbreaks rapidly. 3. A legionella crisis plan will be developed by all regional health boards. 4. 24/7 on-call duty: relevant organisations and the relevant national reference laboratories will be on duty around the clock. 5. Crisis centre and communication: A daily meeting is planned, including teleconferences with the ECDC and the relevant regional health boards, and briefings with the food safety sector (as well as with the veterinary sector, if necessary). | 1.Reporting (enhanced surveillance): The health authorities at the participating sites will send information on outbreaks and special events daily. In addition, the reference laboratories will report daily on potential clusters of confirmed cases in order to support the identification of outbreaks (including inter-regional and international outbreaks). 2.Information: A log book will be kept containing the daily notifications and the situation regarding infectious diseases in the European Union and the other countries participating in the championship. The basis for this information will be the Early Warning and Response System (EWRS), the Rapid Alert System for Biological and Chemical Alerts and Threats (RASBICHAT), the Medical Intelligence System of the European Commission (MEDISys) and the Health Emergencies and Diseases Surveillance System (HEDIS). | 182 |
| 2008 | Austria | The 2008 European Football Championship Switzerland, Austria | food-borne meningitis, SARS, botulism, H5N1, anthrax, measles, meningitis, poliomyelitis, rabies, HIV, tuberculosis or legionellosis, seasonal flu, Bordetella pertussis | Gastrointestinal/Respiratory tract/Blood-borne | / | / | 1.Surveillance, crisis management and national coordination: FOPH staff responsible for early warning, communicable disease surveillance, project coordination and communication will meet daily to evaluate national surveillance data and international information on infectious diseases provided by relevant organisations. A daily report will be produced and made available. In the case of a public health event, the crisis management organisation will become operational and national and cantonal public health partners will communicate through telephone conferences. 2.Outbreak investigation: The content of the notification form for ‘clusters of cases and unusual events’ has been updated and made available on the internet. Local infectious disease outbreaks must be reported within 24 hours.  3.Communication: The FOPH EURO 2008 public website provides related information. A situation report on measles and recommendations on immunisation against measles and tick-borne encephalitis have been posted. During EURO 2008, a national telephone hotline will be available for the public to answer questions concerning infectious diseases. 4. Preparedness: During Euro 2008, the FOPH and national reference laboratories will be on-call 24 hours a day seven days a week for international, national and cantonal (public health) authorities. 5.HIV prevention: A team of the Swiss AIDS Federation’s project ‘Don Juan’ will provide information on rules of safer sex, distribute postcards with rules of ‘fair play’ for commercial sex, and hand out condoms to men in locations where commercial sex is available. 6.Training: FOPH staff and cantonal chief medical officers of host cities participated in a workshop dealing with mass gatherings in Vienna in May 2008, which was organised for the Austrian health authorities and the food safety sector by the ECDC and the Robert Koch Institute, Germany. 7. Food safety: Official food control in Switzerland is carried out by cantonal food control officers. The FOPH will be responsible for the coordination of cantonal actions and communication in case of an event of national concern. | In Switzerland, the national communicable diseases surveillance is coordinated by the FOPH (the Federal Office of Public Health) and includes: 1.A mandatory laboratory- and physician-based reporting system for 43 notifiable infectious diseases with a notification delay of two hours (e.g. SARS, botulism, H5N1 or anthrax), 24 hours (e.g. measles, meningitis, poliomyelitis, rabies, clusters of cases* and unusual events**) or one week (e.g. HIV, tuberculosis or legionellosis); 2.A sentinel surveillance system based on primary healthcare physicians voluntarily reporting on seasonal flu, Bordetella pertussis and other topics; 3.An early warning and information network (EWIN) which retrieves data on a daily basis from international electronic information tools (ProMed, the Global Public Health Intelligence Network GPHIN, the Health Emergencies and Diseases Surveillance System (HEDIS), etc.), from the media and from national crisis information networks; 4. A crisis management team. | 183 |
| 2008 | China | The 2008 Olympics Beijing, China | influenza/Acute Respiratory Syndrome (SARS) | Respiratory tract/Gastrointestinal/Blood-borne/Vector | / | 1.Mass gatherings present a particular challenge for public health. 2.Possible infectious public health threats for visitors to China may include respiratory, diarrhoeal diseases, sexually transmitted infections and some vector borne diseases in rural areas (including dengue, Japanese encephalitis, malaria) as well as animal bites with possible exposure to rabies. Among the respiratory diseases, seasonal influenza has begun in Southern China (Hong Kong Special Administrative Region). | Daily audio conferences with EU/EEA countries and the European Commission have been scheduled during this period, and a specific daily bulletin summarising detected possible health threats made available by email to all EU/EEA Member States authorities, the European Commission, China CDC and WHO. | Daily epidemic intelligence activities at the ECDC have been enhanced to cover available data and media news concerning the Olympic Games and China. A team of four epidemic intelligence officers at ECDC will monitor everyday media news in European languages and Chinese on the internet. | 184 |
| 2008 | China | The 2008 Olympics Beijing, China | Influenza/Japanese B encephalitis/Rabie/Hepatitis B | Respiratory tract/Gastrointestinal/Blood-borne | / | 1. AIDS/HIV: increasing heterosexual transmission  2. Avian flu: avoid bird markets and poultry farms  3. Dengue fever: avoid mosquito bites in daytime  4. Gastroenteritis: avoid risky food, eat at the Village  5. Leishmaniasis: avoid sandfly bites  6. Rabies: seek immediate medical attention after any animal bite | Immunisations: Routine, Influenza, Rabies, Typhoid, Hepatitis A, Hepatitis B. | / | 185 |
| 2010 | India | The 2010 Delhi XIX Commonwealth Games India | Multi-infectious disease | Vector/Respiratory tract/Gastrointestinal/Blood-borne/Water-contact | / | With such a large mass gathering expected for the 2010 Commonwealth Games, the potential for disease outbreaks are especially high in a country of great social extremes. | 1.At each of the stadia, a Medical Centre with basic emergency care facilities to provide on the spot medical care is being established. First aid posts at the competition venues will provide on the spot care to the spectators.  2.Vaccine: Cholera, Diphtheria, Hepatitis ‘A’, Hepatitis ‘B’, Japanese encephalitis, Meningococcus, Measles, Mumps, Rubella, Polio Rabies, Tuberculosis (TB), Tetanus, Typhoid fever, Yellow fever. | / | 186 |
| 2010 | South Africa | The 2010 FIFA World Cup South Africa | polio/measles/influenza/food-borne disease/meningococcal/sexually transmitted infection/vector-borne | Vector/Respiratory tract/Gastrointestinal/Blood-borne/Water-contact | / | 1.International mass gatherings pose specific challenges. 2.A measles outbreak that started in early 2009 in Gauteng Province has spread to a number of other regions 3.The country remains vulnerable to reintroduction of wild-type polio, given suboptimal vaccine coverage in some areas. 4.The risk of acquiring African tropical infections, ie,yellow fever; Mosquito-borne diseases 5.South Africa is regarded as a rabies-endemic country, mainly related to dog exposure | 1.Pretravel preparation and appropriate vaccinations 2.As with malaria, DEET-based insect repellents and protective clothing should be used by those exploring the bush and other outdoor areas of South Africa to reduce the risk of tick bites and hence African tick bite fever (ATBF). | / | 187 |
| 2011 | Greece | The 2011 Summer Special Olympics Greece | Legionellosis/Vector borne/Sexually transmitted diseases/Food borne | Vector/Respiratory tract/Gastrointestinal/Blood-borne/Water-contact | / | Mass athletic gatherings represent a significant challenge for public health. | 1. Vaccine: tetanus-diphtheria vaccine; measles, mumps, and rubella vaccine; Hepatitis B vaccine; Hepatitis A vaccine 2.During the games (1) Food borne diseases: Travellers should avoid food that has been kept for long time at room or ambient temperature (e.g. uncovered buffet food, food from street and beach vendors), uncooked food apart from fruit and vegetables that can be peeled or shelled, fruits with damaged skin, dishes containing raw or undercooked eggs, and ice cream from unreliable sources, including street vendors. (2) Legionellosis: Prevention of infection depends on regular cleaning and disinfection of possible sources. (3) Vector borne diseases: Leishmaniasis, West Nile virus (WNV), West Nile virus (WNV) Take appropriate insect bite precautions when they visit areas with mosquitoes, ticks, sand flies, and fleas, including wearing appropriate clothing, such as long-sleeved shirts, long pants, hats, and closed shoes, using insect repellent with 30e50% DEET (N,N-diethyl-3-methylbenzamide) and avoiding outdoor activities, especially from dusk to dawn.  (4) Sexually transmitted diseases: Olympic Games visitors avoid casual and unprotected sexual contact and take all appropriate precautions such as using condoms. | / | 188 |
| (1) 2008 (2) 2010 | (1) China (2) South Africa | (1) the Beijing Olympic Games, 2008; (2)the FIFA World Cup in South Africa, 2010 | Multi-infectious disease | Vector/Respiratory tract/Gastrointestinal/Blood-borne/Water-contact | / | / | / | 1.Networks such as GeoSentinel have provided cumulative trends in travelrelated illness to assess pretravel risk for mass gathering events such as the Beijing Olympic Games or the FIFA World Cup in South Africa. 2.GeoSentinel established in 1995, is the major provider-based surveillance network for travelrelated illness. The GeoSentinel communications and data collection network currently comprises 53 travel/tropical medicine International Society of Travel Medicine (ISTM) member clinics located in 25 countries on all 6 continents. GeoSentinel surveillance data enable patient diagnoses, country of exposure, chronology of travel, and standardized exposure details to be collected for detailed analysis of travel-related morbidity. | 189 |
| 2012 | Ukraine, Poland | 2012 UEFA European Football Championship Poland, Ukraine | Measles | Respiratory tract | / | A measles outbreak in Ukraine with more than 11,000 cases from the beginning of 2012 until the end of June 2012. Susceptible visitors not only had a high risk of being infected, but also geographically propagating the epidemic to other countries. | To prevent imported epidemics, it should be emphasized that vaccinating travellers would most efficiently reduce the risk of epidemic. | 1. The study propose a novel two-phase multitype branching process model with immigration to describe the risk of a major epidemic in connection with large-scale sports-related mass gathering events. By analytic means, the expected number and the variance ofimported cases and the probability of a major epidemic caused by the imported cases in their home country were calculated. 2.A discrete time Markov chainmodel was introduced, which is an adaptation of a multitype Galton-Watson process with immigration to give a mathematical model for the evolution of the epidemic. | 190 |
| (1) 2008 (2) 2012 | (1) China (2) Britain | (1) the 2008 Paralympic Olympics  (2) the 2012 Paralympic Olympics | Multi-infectious disease | Vector/Respiratory tract/Gastrointestinal/Blood-borne/Water-contact | In both Paralympic Games, respiratory tract infections (RTIs) were the most frequent: incidence rate increased from 15.2 in Beijing to 18.1 in London/1000 athlete-days (95% CI 9.7 to 20.7 vs 11.5 to 24.7). | / | Strengthen the importance of regular prophylactic multispecialist medical examinations. | / | 191 |
| 2014 | Brazil | The 2014 FIFA World Cup Brazil | Dengue | Vector | / | / | / | A spatiotemporal hierarchical Bayesian modelling framework was used. The forecasts for June, 2014, showed that dengue risk was likely to be low in the host cities Brasília, Cuiabá, Curitiba, Porto Alegre, and São Paulo. The risk was medium in Rio de Janeiro, Belo Horizonte, Salvador, and Manaus. High-risk alerts were triggered for the northeastern cities of Recife, Fortaleza, and Natal. | 192 |
| 2010 | South Africa | The 2010 FIFA World Cup South Africa | tick bite fever, Crimean Congo haemorrhagic fever, chikungunya, cholera, dengue, food-borne disease, hand, foot and mouth disease, human immunodeficiency virus (HIV) infection, influenza, legionellosis, malaria, measles, meningococcal meningitis, sexually transmitted infections, poliomyelitis, rabies, Rift Valley fever, respiratory syncytial virus infection, rubella, tuberculosis and yellow fever. | Vector/Respiratory tract/Gastrointestinal/Blood-borne/Water-contact | / | Large numbers of visitors in the same area at the same time may increase the risk of communicable disease outbreaks. Several factors contribute to this theoretical increase, such as increased person-to-person transmission of pathogens due to the localised high population density, risk of importation of non‐endemic diseases, exportation of endemic diseases, challenges in contact tracing due to visitor mobility and temporary structures such as mass catering and accommodation for visitors. | / | 1. A set of three specifically adapted alerts in the web-based screening system MedISys were set up: potential public health events in South Africa, those occurring in the participating countries and those in the rest of the world. 2. The list of diseases comprised the following:  tick bite fever, Crimean Congo haemorrhagic fever, chikungunya, cholera, dengue, food-borne disease, hand, foot and mouth disease, human immunodeficiency virus (HIV) infection, influenza, legionellosis, malaria, measles, meningococcal meningitis, sexually transmitted infections, poliomyelitis, rabies, Rift Valley fever, respiratory syncytial virus infection, rubella, tuberculosis and yellow fever. | 193 |
| 2018 | Britain | The 2018 Commonwealth Games Triathlon Broadwater, Britain | Multi-infectious disease | Respiratory tract | / | 1. Mass gatherings provide ideal conditions for influenza transmission and amplification of preseasonal viruses, even in contexts of low seasonal influenza activity.  2. Cases of Neisseria meningitides type b and leptospirosis occurring during sporting events have been documented, the latter associated with heavy rains. | / | / | 194 |
| 2014 | Brazil | The 2014 FIFA World Cup Brazil | Dengue | Vector | / | The risk of dengue among tourists during the World Cup is expected to be small due to immunity among the Brazil host population provided by last year’s epidemic with the same DENV serotypes. | / | Two statistical models were used: a percentile rank (PR) and an Empirical Bayes (EB) model. | 195 |
| 2014 | Brazil | The 2014 FIFA World Cup Brazil | Dengue | Vector | 1. The density of cases becomes residual in June–August, and during the World Cup period (June 12–July 13, 2014), only Fortaleza reported a small outbreak of 0·4 cases per 1000 inhabitants. Natal, Recife, and Salvador, with fewer than 0·08 cases per 1000 inhabitants. 2. So far, only three cases of dengue were confirmed during the 2014 FIFA World Cup. | / | / | / | 196 |
| / | Around the world | Olympic Games, World Cups and other global sporting events | Multi-infectious disease | Respiratory tract/Gastrointestinal | / | A major sporting event that brings together large groups of people in circumstances that optimize disease transmission also brings potential public health risks. | / | The requirement for improved surveillance and reporting systems enhanced the public health service in the United Kingdom. | 197 |
| 2016 | Brazil | The 2016 Rio Olympics  Rio, Brazil | Zika | Vector | / | / | WHO’s statement advised athletes and visitors travelling to the games in Rio to practise safe sex, choose air conditioned accommodation, use insect repellent, and wear light coloured clothing that covers as much of the body as possible. | / | 198 |
| 2014 | Brazil | The 2014 FIFA World Cup Brazil | Dengue | Vector | / | From 12 June to 13 July 2014, Brazil hosted the 2014 Federation Internationale de Football Association (FIFA) World Cup, a mass gathering of more than 3 million Brazilian and international spectators, travelling between 12 different host cities. | / | A spatio-temporal Bayesian hierarchical model was used. The forecast model produced more hits and fewer missed events than the null model, with a hit rate of 57% for the forecast model compared to 33% for the null model. | 199 |
| 2016 | Brazil | The 2016 Rio Olympics  Rio, Brazil | Zika | Vector | / | / | 1. Follow the travel advice provided by the country’s health authorities, and consult a health worker before travelling. 2. Whenever possible, during the day, protect themselves from mosquito bites by using insect repellents and by wearing clothing – preferably light-coloured – that covers as much of the body as possible. 3. Practise safer sex (for example, use condoms correctly and consistently) or abstain from sex during their stay and for at least eight weeks after their return, particularly if they have had or are experiencing symptoms of Zika virus infection. 4. Choose air-conditioned accommodation (where windows and doors are usually kept closed to prevent the cool air from escaping, and where mosquitoes cannot enter the rooms). 5. Avoid visiting areas in cities and towns with no piped water or poor sanitation (ideal breeding grounds of mosquitoes), where the risk of being bitten by mosquitoes is higher. | / | 200 |
| 2015 | France | An 2015 obstacle adventure race Alpes-Maritimes, France | norovirus | Gastrointestinal | Of 8,229 persons registered, 1,264 adults reported acute gastroenteritis (AG) resolved within 48 hours. Of adults who reported AG, 866 met the case definition. | Younger participants (aged 18–27) years had a significantly higher risk or acute gastroenteritis (AG) in the multivariate analysis compared with older participants. The risk of AG was higher in the morning, from 10:00 to 14:00 and still high in the afternoon compared with the first hour of departure waves (9:00–10:00). The ingestion of mud is also a significant risk factor of AG. | / | / | 201 |
| 2016 | Brazil | The 2016 Rio Olympics  Rio, Brazil | Zika | Vector | / | 1. The Zika virus outbreak in Brazil and the associated excess of microcephaly cases are concentrated in the Northeast Region (92% of confirmed cases), well away from Rio in the Southeast Region.  2. Athletes and spectators are likely to spend their time in places purged of mosquito breeding sites and sprayed heavily with insecticide, so games participants are unlikely to encounter mosquitoes unless they travel well away from “official” sites. | / | / | 202 |
| 2016 | Brazil | The 2016 Rio Olympics  Rio, Brazil | Multi-infectious disease | Vector | / | / | 1. Recommendations to postpone the games fail to recognize this epidemiological pattern, and would put athletes and visitors under a much higher risk of infection. 2. Daily sweeps will be conducted in all Olympic facilities and areas where sport events will take place. 3. Approximately 2500 health professionals will be added to the current staff to intensify the health care network during the Games. 4. The private sector could take action and sponsor, for example, the distribution of a kit e containing preventative instructions against A. aegypti, a bottle of repellent, and condoms e to those attending sport events during the Games. | / | 203 |
| 2016 | Brazil | The 2016 Rio Olympics  Rio, Brazil | Zika | Vector | / | The spiky spread of ZIKV and its epidemic potential are especially problematic in countries which host big mass gatherings with endogenous ZIKV circulation. | 1.Health education 2.Intensifying epidemiological surveillance 3.Developing and monitoring global MG-specific integrated surveillance and alert system 4.Establishment and maintaining of “Zika Active Pregnancy Surveillance System” and prevention of perinatal transmission 5.Establishing and Maintaining Travel Medicine 6.Personal Protection 7.Environmental sanitation 8.Treatment 9.Disinfections 10.Financial support and commitment 11.Multidisciplinary international cooperation 12.Future vaccination 13.Intensifying compulsory vaccination against yellow fever 14.Control measures are needed for similar Arbovirus 15.Research and development of new products | / | 204 |
| 2016 | Brazil | The 2016 Rio Olympics  Rio, Brazil | Zika | Vector | / | / | Pregnant women should avoid visiting any region of the world where Zika virus circulation has been reported, including Rio de Janeiro, a recommendation in line with international and national public health guidelines. | / | 205 |
| 2016 | Brazil | The 2016 Rio Olympics  Rio, Brazil | Zika | Vector | / | 1. All countries are at risk for travel-associated importation of Zika virus.  2. CDC identified 19 countries currently not reporting Zika outbreaks but with environmental conditions and population susceptibility that could sustain mosquito-borne transmission of Zika virus during August–September. | 1. Pregnant women should not travel to any area where Zika virus transmission is ongoing. 2. Travelers should take protective measures, including use of insect repellent, to prevent mosquito bites both during travel and for 3 weeks after returning to their home country. Such measures include wearing long-sleeved shirts and long pants; staying in places with air conditioning and window and door screens to keep mosquitoes outside; sleeping under a mosquito bed net, and using insect repellents with active ingredients (e.g., DEET). 3. Travelers should prevent possible sexual transmission while at the 2016 Olympic and Paralympic Games and after returning home by correctly using condoms every time they have sex or by abstaining from sex. Males should use condoms for at least 8 weeks after travel or, if symptomatic for Zika virus infection, for 6 months from the start of symptoms. 4. After returning from a country with Zika virus transmission, men with pregnant partners should use condoms or not have sex for the duration of the pregnancy. 5. Couples who want to try to get pregnant after attending the Olympic and Paralympic Games should wait at least 8 weeks, and 6 months if the male partner has symptomatic Zika virus infection. | A stepwise model was used to assess the country-specific risk for importation and sustained, local mosquito-borne transmission of Zika virus from travel to the Games. | 206 |
| 2016 | Brazil | The 2016 Rio Olympics  Rio, Brazil | Zika | Vector | / | 1. There remains some legitimate concern about the possibility of Zika virus being exported from Rio to vulnerable regions of Africa. 2. The potential of mass migration to reignite recently halted measles and rubella transmission in Brazil, and to trigger the spread of influenza from Brazil to other countries.  3. Weaknesses in Brazil sanitation system makes water-borne infections a threat to Rio’s visitors and an ongoing health threat to millions of Brazilians long after the Olympics and Paralympics have left town. | 1. Pregnant women are advised to avoid travelling to the games; visitors are advised to use mosquito repellents and to cover up where possible. 2. Safe sex messaging is being clearly promoted (up to 8 weeks after the games for asymptomatic individuals, or for 6 months if Zika virus disease symptoms have developed). | / | 207 |
| (1) 2009 (2) 2013 (3) 2015 | (1) Rome, Italy (2) Barcelona, Spain (3) Kazan, Russia | The 2015, 2013 and 2009 Fédération Internationale de Natation (FINA) World Championships | Multi-infectious disease | Respiratory tract/Gastrointestinal | 1. The most common illnesses were infections of the respiratory (33.9%) and gastrointestinal tract (23.5%).  2. At all three championships, infections of the respiratory (32.6%, 24.7%, 34.3%) or the gastrointestinal tract (20.4%, 23.2%, 23.7%) and otitis (17.1%, 15.5%, 9.6%) were most common. | / | These findings emphasise the need for effective injury management and prevention strategies in aquatics. We recommend (1) a sport-specific approach to prevention, (2) out-of-competition injury surveillance and (3) improving athlete health education. | / | 208 |
| 2018 | Russia | The 2018 FIFA World Cup Russia | Multi-infectious disease | Vector | / | / | 1. Strengthen epizootiological monitoring when performing the survey of green areas in FWC-2018 host-cities, especially natural biotopes adjacent to the sports event venues, as well as territories of the popular tourist hiking routes. 2. On increase in scope, terms and frequency of non-specific preventive measures in areas of high risk of infection. | / | 209 |
| 2015 | Saudi Arabia | Saudi Professional League, the 2015 2016 Season | Multi-infectious disease | Respiratory tract/Gastrointestinal | Seventy-five illnesses were reported in 63 players, with the most common diagnoses being respiratory tract infections (n = 33, 44%) and gastroenteritis (n = 14, 18%). | / | / | / | 210 |
| 2018 | Korea | The 2018 Winter Olympics PyeongChang, Korea | Multi-infectious disease | Respiratory tract | Upper respiratory infection was the most frequent case in diseases of athlete encounters. | / | 1. It is necessary to operate the polyclinic resiliently according to the game schedule, sport entries and the geographical factors such as the climate. 2. Operation of MRI equipment in the PyeongChang Winter Olympic Games suggests that the equipment of the polyclinic should be considered for the games and after the games. | / | 211 |
| 2016 | Brazil | The 2016 Rio Olympics  Rio, Brazil | Zika | Vector | / | / | Health authorities may wish to actively monitor Twitter in order to rapidly identify areas in which the public require more information and clarifications on. This infor-mation can then be disseminated through social media and/or more traditional means. In the specific case of another Zika virus outbreak, health authorities may wish to focus on providing information highlighting at risk groups, symptoms transmission, prevention, vaccines, and whether it is safe to travel to at risk geographical zones. For future outbreaks, health authorities could disseminate infor-mation on the work they have been doing around Zika on Twitter. | / | 212 |
| 2018 | Russia | The 2018 FIFA World Cup Russia | Measles | Respiratory tract | / | "Risk territories" are RF regions located at the state borders as well as regions where epidemiologic surveillance over measles is inefficient and there hasn't been sufficient immunization against the disease. These regions are Moscow city, Saint Petersburg, Voronezh region, Moscow region, Kaliningrad region, Samara region, Amur region, Rostov region, Sverdlovsk region, Krasnodar region, the Chechen Republic, Dagestan, Primorye, and Khabarovsk region. | / | A procedure for assessing risks of the measles virus being imported and spread in certain RF regions. | 213 |
| 2020 | Japan | 2020 Tokyo Olympic Games | COVID-19 | Respiratory tract | / | Although inevitable as an alternative to contain COVID-19 contamination, social withdrawal can damage the immune system, compromise sports performance by altering body composition, impairing cardiorespiratory fitness, muscle strength and control, and mental and physical health. | / | / | 214 |
| 2020 | Japan | The 2020 Olympics Tokyo, Japan | Multi-infectious disease | Respiratory tract | / | The resurgence of measles and vaccine hesitancy is a threat to global health, and Japan has been no exception. The number of measles patients in Japan amounted to 744 in 2019. 2.The re-emergence of rubella epidemic has been detected in Japan since 2018, and as of 29 January 2020, the cumulative cases amounted to 5,280 along with 5 cases of congenital rubella syndrome. | Strengthening contemporary health promotion campaigns for the general public and reinforcing evidencebased supplementary vaccination programs for susceptible pockets are imperative. In addition, education for healthcare providers is indispensable in Japan. Moreover, incorporating vaccine records into the individual number card which will function as a health insurance card from fiscal year 2020 is helpful. Considering the Japan's political stability, enforcing legal procedures for mandatory vaccination can be an option. | / | 215 |
| 2018 | Australia | The 2018 Commonwealth Games, Queensland, Australia | Multi-infectious disease | Respiratory tract/Gastrointestinal/Blood-borne | Over the study period, there were 10 595 patient presentations to the emergency department (ED), the most common communicable disease profile was respiratory in nature (51.4%, n = 772). | / | 1. Depending on the communicable disease, the suggestion of an assessment clinic separate from the ED may be useful to implement to improve ED capacity and reduce the spread of infection.  2. Furthermore, specific training programmes for ED staff in recognizing the SIs associated with certain communicable diseases may be of benefit. | / | 216 |
| 2020 | Japan | The 2020 Olympics Tokyo, Japan | COVID-19 | Respiratory tract | / | / | Travels and airports: One of the main recommendations to travelers, especially athletes in competition, even being palliative, is to isolate any athlete with respiratory symptoms during the entire travel and to maintain a distance of at least two rows of chairs . | / | 217 |
| 2019 | Russia | The 1st Winter Children of Asia Games Yuzhno-Sakhalinsk in Sakhalin, Russia | Multi-infectious disease | Respiratory tract | In the Thailand National Youth Team (18 Thai athletes), the upper respiratory tract was the site most affected by illness (67%) with females reporting a greater illness rate. | The physiological responses of tropical country athletes who partake in winter sport may be impaired under cold environmental conditions., repeated exposure to cold air during sports, likely exposes athletes to an increased risk for upper-respiratory-tract infections. | / | / | 218 |
| 2009 | Australia | The 2009 17th World Transplant Games.  Gold Coast, Australia | H1N1 | Respiratory tract | No documented cases of H1N1 were identified during the Games. | / | / | / | 219 |
| 2005 | United States | The 2005 Adventure Race Tampa, Florida, United States | Leptospirosis | Water-contact | 44 (23%) of 192 racers met the definition for a suspected case. 14 (45%) of the 31 patients with suspected cases who were tested had their cases confirmed by serological testing (a single sample with MAT titer ≥400), including the index case patient. Organisms of a potential novel serovar (species Leptospira noguchii) were isolated in culture from 1 case patient. | 1. Direct contact with infected animals or their urine or through contact with contaminated environments. 2. Swallowing river water, swallowing swamp water, and being submerged in any water.  3. Outbreaks may be precipitated by heavy rainfall and flooding and can occur in areas where the disease has not previously been considered to be endemic. 4. Increasing urban human and rodent populations, occupational and avocational exposure to surface water, expanding canine leptospirosis, and climatic changes all have been associated with changes in the epidemiology of human leptospirosis. Among these factors, climate change holds perhaps the greatest potential to affect the transmission of leptospires to humans. | 1. Primary chemoprophylaxis has been demonstrated to be effective at reducing the risk of leptospirosis during periods of exposure to surface water. 2. Postexposure prophylaxis may protect against the development of symptomatic leptospirosis but not against seroconversion.  3. The potential benefits of short-term primary chemoprophylaxis with doxycycline likely outweigh the risks, because the morbidity associated with leptospirosis can be high. | 1. CDC and participating state and local health departments contacted race participants and administered a questionnaire to assess the presence of acute illness, medical care, and specific activities and behaviors during the race. 2. Lab investigation: Serum specimens for anti-leptospiral antibody testing and blood and urine specimens for culture were requested from all individuals with suspected cases for submission to the CDC through the individuals’ health care providers. 3. Environmental investigation: Environmental samples were collected on 17 December 2005, 6 weeks after the race, from areas along the race course that were identified by racers and race organizers as the sites with the most-extreme water exposure or that showed evidence of potential animal reservoirs in the park (eg, muddy areas rooted up by pigs). | 220 |
| 2006 | Germany | The 2006 triathlon in Germany Heidelberg and in the Neckar river, Germany | Leptospirosis | Water-contact | A total of 142 of 507 triathletes were contacted; among these, five confirmed leptospirosis cases were found. | 1. Swimming in the Neckar River in Heidelberg during a triathlon three days before onset of symptoms as the most likely source of infection. 2. A disrupted skin barrier is the classically acknowledged route of transmission, and we found that the presence of wounds before swimming was associated with the infection. Open wounds were identified as the only significant risk factor for illness.  3. Heavy rains that preceded the swimming event likely increased leptospiral contamination of the Neckar River. | Protective clothing and/or covering lesions with waterproof dressings may decrease the risk of leptospirosis in individuals exposed to potentially contaminated water. | We conducted a retrospective cohort study among the triathletes officially registered for the event. The participants were contacted by e-mail, and were asked to fill out a standardized questionnaire. | 221 |
| 2012 | British | The 2012 London Olympics London, British | Not specified | Not specified | / | / | / | A dynamic risk assessment model for Infectious disease embedded in a pilot Decision Support System (DSS) conceived via various regional and national Delphi workshops.  1. HPZone: a DSS for the management of Enquiries, Cases and Outbreaks at 26 Local Units readily providing consistent data. Basic coincidence and threshold alerts are built in so appropriate information is automatically brought to the attention of those who need to know. 2. HPZone-Cosurv interface: A module which automatically harvests laboratory data from encrypted emails and incorporates them into HPZone with tight rules for data insertion. 3. Dashboard: a module providing aggregate Case and Outbreak data collected and presented in different views including geographic, temporal and disease specific. | 222 |
| 2012 | British | The 2012 London Olympics London, British | Sexually Transmitted Infections | Blood-borne | A total of 30 STIs were diagnosed among OVs including non-specific urethritis (10, 9%), chlamydia (5, 5%), warts (5, 5%), herpes (4, 4%), gonorrhoea (3, 3%), molluscum (2, 2%) and scabies (1, 1%). | / | / | / | 223 |
| 2010 | South Africa | The 2010 FIFA World Cup South Africa | Sexually Transmitted Infections | Blood-borne | / | 1. Travellers and their microbial baggage enable a rapid dissemination of infection to all corners of the globe.  2. Ill returning travellers present with predictable or exotic infection that can pose diagnostic challenges. | / | GeoSentinel with 53 sites in 24 countries, including the 17 daughter network sites in Europe – EuroTravNet, collates and analyses surveillance data on ill returning travellers. Surveillance highlights include the geographic mapping of proportionate morbidity of infectious disease by area of acquisition, the elucidation of the spectrum of illness in different traveller populations and in-depth analyses of specific-travel associated illness such as malaria, dengue and sexually transmitted infections (STI). | 224 |
| 2014 | Brazil | The 2014 FIFA World Cup | Not specified | Not specified | / | Although there has been mention of a great deal of planning on the part of the government officials for the World Cup 2014, hospital surge to meet demand still falls short. Only 27% had any surge capacity for isolation of potentially infectious patients, with eight of them having specific isolation rooms or units. Fifteen hospitals (43%) had no plans or space for isolation; five hospitals (14%) would send patients needing isolation to the reference hospital; two hospitals (6%) said they would figure it out when necessary. | / | / | 225 |
| 2012 | Finland | The 2012 European Athletics (EA) Championships Helsinki, Finland | Multi-infectious disease | Respiratory tract/Gastrointestinal | Twenty-seven illnesses were reported, with most of upper respiratory tract infections and gastro-enteritis/diarrhoea. Illness risk factors remains unclear. | / | / | / | 226 |
| 2013 | Sweden | The 2013 European Athletics Indoor Championships Goteburge, Sweden | Multi-infectious disease | Respiratory tract/Gastrointestinal | Among the 577 registered athletes, 29 illnesses were reported (50/1000 registered athletes); upper respiratory tract infection and gastro-enteritis/diarrhoea were the most reported diagnoses. | / | Illness prevention measures should pay attention to temperature and climatic condition changes, air cleaning systems, and implement the recommendations for infectious disease prevention. | / | 227 |
| 2014 | Spain | The 2014 International Sailing Federation Sailing World Championships Santander, Spain | Multi-infectious disease | Respiratory tract/Gastrointestinal | There were 760 respondents (65% of all participants) for the 12-month included in this study. Most illnesses (40%) were infections, primarily of the respiratory system (43%). Of the 29 illnesses (2 per 1000 days of sailing), 9 (31%) were gastrointestinal and 6 (21%) respiratory, while 2 (7%) were gout attacks. | / | / | / | 228 |
| 2016 | Brazil | The 2016 Rio Olympics  Rio, Brazil | Multi-infectious disease | Vector | 48 (11%) participants had antibody evidence of other arboviral infections; 4 (0.9% tested) with CHKV, 6 (1.3%) with DENV, and 39 (9%) with WNV. Of these, new travel-associated infection was confirmed in 32 (7%); 3 of 4 CHKV, 2 of 6 DENV, and 27 of 39 WNV. | Those with travel-associated arboviral infections had similar demographics, and exposure whilst in Brazil, but were less likely to use mosquito repellent than athletes without arboviral infection. | The use of mosquito repellent was associated with decreased risk of infection. | / | 229 |
| 2016 | Brazil | The 2016 Rio Olympics  Rio, Brazil | Zika | Vector | During the Games the highest accumulated incidence observed was for headache: 6.06% cough: 5.30% and conjunctivitis: 3.03%. Non-athletes experienced more incidence of symptoms, except for incidence of cough which was the same in the two groups (1.1%). No participants reported symptoms fulfilling Zika definition case. | / | / | 1. A surveillance platform based on a mobile application installed in participant’s smartphones called “OlymTRIP” that monitored the health status of the Spanish Olympic Delegation (SOD) through a daily interactive check of the user health status including geo-localization data. 2. The app also provided information and advice about Zika infection. In a daily basis the app asked for the health state at the personally set time. In case of feeling unwell, the app provided the contact ofthe doctors of the Spanish Olympic Committee who in addition could track in real-time the health status of the SOD members using a web-based platform. | 230 |
| 2018 | Malaysia | The 2018 Malaysian Higher Education Games, Malaysia | Multi-infectious disease | Respiratory tract/Gastrointestinal | 1. A total of 6071 athletes from 80 contingents took part in SUKIPT 2018. During the nine days of competition 48 illnesses were reported, resulting in an incidence of 0.8 illness per 100 athletes.  2. Infection was the most common cause of illness (n = 25, 52%). A total of 23 illnesses (48%) affected the respiratory system, followed by heat-related illness (n = 8, 16.7%) and the digestive system (n = 5, 10.4%) | / | For future SUKIPT or similar multi-sport events in Malaysia, we recommend the contingent medical teams to monitor injuries and illnesses. This can be achieved by proper communication between tournament and contingent medical teams before the event. This is only possible with cooperation from the event organiser, by making the surveillance official and providing the tournament medical secretariat with contact details of both the Chef de Mission and contingent medical officer. | / | 231 |
| 1982 | South Africa | The 1982 Two Oceans Marathon Cape Town, South Africa | Upper respiratory tract infection (URTI) | Respiratory tract | Symptoms of upper respiratory tract (URT) infection occurred in 33.3% of the 141 runners compared with 15.3% of controls, and were most common in those who achieved the faster race times. | 1. Extreme stress and fatigue; 2. Cold and dry air; 3. Acute stress. | / | A randomly selected sample of 170 athletes entered for the marathon were asked to complete a questionnaire on the day before the race. Two weeks after completion of the race, 150 of the original 170 subjects were questioned regarding URT symptoms during the 14-day post-race period. | 232 |
| 1987 | United States | The 1987 Los Angeles Marathon (LAM) Los Angeles, United States | Flu | Respiratory tract | During the week before the race, 342 runners reported an infectious episode. Of these, 132 (38.6%) participated in the race and were still sick the week after (176 participated and were not sick the week after, 34 chose not to run). | 1. The mucosal immune system is suppressed by prolonged exercise. 2. The negative relationship between stress and infectious episodes. | / | A questionnaire including questions on demographic data, training habits, race results, and the incidence of infectious episodes was developed and administered to applicants of the marathon. | 233 |
| 1999 | Namche Bazaar | The 1999 Everest marathon Namche Bazaar | Multi-infectious disease | Respiratory tract/Gastrointestinal | 1. There were 65 recorded consultations: 41 were treated as bacterial, 34 being treated with ciprofloxacin and seven with ofloxacin; 17 cases of giardia were diagnosed clinically. 2. 19 consultations were made for a cough. 6 athletes were diagnosed as having chest infections and treated with amoxicillin unless allergic. | The remoteness of the Everest marathon, the length of time spent away from medical aid, the cold, altitude, and seriousness of the terrain. | 1. On race day, there were aid and drink posts, with a doctor, every 3 miles.  2. A backup team with a doctor swept down the course on the heels of the back markers assessing their ability to continue.  3. Runners were given clear instructions before the race about what to do if they became exhausted or were injured and also how to react to finding another injured runner. 4. Education of the athletes, before and during the trek, about adequate hydration, personal hygiene, and eVects of altitude helped them to distinguish the normal from the abnormal and encouraged them to seek medical advice sensibly. | / | 234 |
| 2000 | Malaysia | Eco-Challenge-Sabah 2000 Borneo, Malaysia | Leptospirosis | Water-contact | Of the 158 athletes contacted, 109 reported illness; 68 (44%) had illness that met the case definition. | 1. Leptospira were the cause of illness and that water from the Segama River was the primary source of infection.  2. On univariate analysis, statistically significant risk factors for illness included kayaking, swimming in the Segama River, swallowing water from the Segama River, and spelunking. By multivariate stepwise logistic regression, only swimming in the Segama River was independently associated with illness. Adjusting for the other variables did not alter the relative risk. The attributable risk of river swimming was 38%. | / | 1. To identify cases of febrile illness occuring at the time of the race, a standardized telephone questionnaire wa administered to the athletes. 2. Ill athletes from whom serum specimens had been drawn by their physicians were requested to submit samples to CDC. | 235 |
| 2002 | United States | The 2002 Winter Olympics Salt Lake City, United States | influenza/meningitis | Respiratory tract/Gastrointestinal/Blood-borne | / | / | 1.Volunteers as well as competitors to take the influenza vaccine, and suggested that athletes also be immunised against meningitis. 2.Have fully staffed medical stations at every competition and training venue for the athletes. 3.Emergency treatments must follow strict protocols. | / | 236 |
| 2002 | United States | The 2002 U.S. Transplant Games theme park A in Orlando, Florida | Salmonella serotype Javiana | Gastrointestinal | 1. 2 cases of Salmonella serotype Javiana infections among persons who had attended the 2002 U.S. Transplant Games held at theme park A in Orlando, Florida, during June 25-29. 2. A total of 369 (34%) persons responded by August 1; of these, 296 (80%) responded by July 22. 94 (25%) persons reported that at least one household member had an illness that met the case definition, representing 141 ill persons. | Preliminary microbiologic evaluation indicates fecal coliform contamination of the diced tomatoes. | / | 1. For case ascertainment and investigation purposes, a web-based survey was distributed electronically on July 20 to 1,100 attendees with known e-mail addresses, including athletes, donors, family members, and transplant professionals.  2. To identify other potential cases of S. Javiana, the PFGE pattern for the outbreak strain was posted on PulseNet, the National Molecular Subtyping Network for Foodborne Disease Surveillance. | 237 |
| 2003 | United States | The 2003 America’s Cup yacht racing | Upper respiratory tract infection (URTI) | Respiratory tract | In total, 220 injuries and 119 illnesses were recorded, with an overall incidence of 8.8 incidents/ 1000 sailing and training hours (injuries, 5.7; illnesses, 3.1). Most of the illnesses were upper respiratory tract infections (40%). | The high incidence of URTIs may be attributed to the stresses associated with the America’s Cup—for example, living in hotels away from home for long periods of time, intense training and sailing demands, long working days (with most days in excess of 12 hours), little recovery time or ‘‘time off’’, cold and wet weather conditions, and routine monotony, all of which could increase stress and contribute to suppressed immune system function and increased risk of URTIs. | 1. Prudent allocation of preventive and therapeutic resources, such as comprehensive health and medical care, well designed conditioning and nutritional programmes, and appropriate management of recovery should be adopted by America’s Cup teams in order to reduce the risk of injury and illness. 2. Nutritional strategies, such as maintaining a positive energy balance, consuming a well balanced diet high in carbohydrate, protein, and micronutrients, and the intake of carbohydrate before, during, and after training, as well as regular monitoring of markers of fatigue and overtraining are reported to help prevent or minimise the risk of immunosuppression and subsequent URTIs. 3. Appropriate attention should be placed on the health of the athletes, including regular health and medical assessments, comprehensive medical treatment, physiotherapy (prehabilitation and rehabilitation), preventive strength and conditioning programmes, nutrition and hydration strategies, as well as the monitoring of total work loads in order to manage and implement appropriate recovery strategies. | 1. All injuries and illnesses were diagnosed and reported by medical staff, which included a sports physician, a general practitioner, a physiotherapist, and a chiropractor, on a standard injury report form. 2. A prospective study design was used to collect the injury and training data of a professional America’s Cup yacht racing crew during the preparation for and participation in the Challenge for the 2003 America’s Cup. | 238 |
| 2004 | Greece | The 2004 Olympics Athens, Greece | Legionnaires | Respiratory tract | / | / | / | Scoring system and quantitative assessment for Water supply system, Cooling tower and Decorative fountain. | 239 |
| 2003 | New Zealand | The 2003 Southern Traverse Adventure Race New Zealand | Upper respiratory tract infection (URTI) | Respiratory tract | 1. Symptoms of upper respiratory illness were most common immediately before (25/60, 42%) and after (28/49, 57%) racing, and largely resolved over the 2-week follow-up (5/27, 19%). 2. Gastrointestinal complaints were common at the finish (8/49, 16%) and during the next 5 days but settled more quickly than upper respiratory symptoms. | 1. Low levels of salivary immunoglobulin A (SIgA) are associated with an increased risk of URTI 2. Overtraining. 3. Quality of drinking water. | / | Illness, injury, and mood data were obtained using questionnaires. Questionnaires were first administered and completed 2 days before the race, once teams had undergone prerace registration. | 240 |
| 2021 | Japan | Olympics | COVID-19 | Respiratory tract | / | 1. Vaccine rollout has been inequitable, reducing access in many low and middle income countries. The country’s limited testing capacity and sluggish vaccine rollout have been attributed to lack of political leadership. Even healthcare workers and other high risk populations will not have access to vaccines before Tokyo 2020, to say nothing of the general population. 2. An overwhelmed healthcare system combined with an ineffective test, trace, and isolate scheme could seriously undermine Japan’s ability to manage Tokyo 2020 safely and contain any outbreak caused by mass mobilisation. | Must accelerate efforts towards containing and ending the pandemic by maintaining public health and social measures, promoting behaviour change, disseminating vaccines widely, and strengthening health systems. | / | 241 |
| 2021 | Japan | Olympics | COVID-19 | Respiratory tract | / | / | Large-scale vaccination will effectively control the spread of COVID-19. When the protective efficacy of vaccines is 78.1% or 89.8%, and if the vaccination rate of athletes reaches 80%, an epidemic prevention barrier can be established. | / | 242 |
| 2020-2021 | United States | National Football League (NFL) and National Collegiate Athletic Association (NCAA) | COVID-19 | Respiratory tract | The median (IQR) daily new COVID-19 cases in treatment group counties hosting games was 26.14 (10.77-50.25) cases per 100 000 residents on game day. The median (IQR) daily new COVID-19 cases in control group counties where no games were played was 24.11 (9.64-48.55) cases per 100 000 residents on game day. | / | The NFL and NCAA policies regarding limited in-person attendance, mask use, and social and physical distancing measures in stadiums was not associated with substantially higher community spread of COVID-19. Additionally, an important number of NFL and NCAA football stadiums are outdoors or have a retractable roof, which could have had an impact on mitigating spread. | / | 243 |
| 2021 | Japan | Olympics | COVID-19 | Respiratory tract | 1. From June 29 to September 8, 2021, more than one million tests were performed for the participants, and 41 OGT/PGT athletes and 822 OGT/PGT non-athlete personnel were confrmed SARS-CoV-2 positive. The estimated incidence was 0.24% [28/11,656] for the OGT athletes, and 0.30% [13/4403] for the PGT athletes. Of 41 athletes, no detailed information was available for 17 cases, only one cluster was identifed in 5 Greek OGT artistic swimming members, and 19 were sporadic throughout various sports. All of them were non-Japanese, and 40 positive cases (97.6%) were detected during the 14-days quarantine at arrival, and only one Italian rowing player 2. For personnel with SARS-CoV-2 in the OGT and PGT, 68.2% and 74.4% were Japanese. Thus, approximately 0.34% [146/43,000] of the foreign OGT non-athletes and 0.56% [67/12,000] of the foreign PGT non-athletes were infected during the study period.r was confrmed after the quarantine. | / | Two core infection control strategies applied in these events were bubble scheme and frequent testing. Vaccination was encouraged, no spectators were allowed, and face mask-wearing were required for all individuals. With regards to vaccination, more than 80% of athletes and staff were vaccinated while the vaccination was not set as mandatory. | / | 244 |
| 2020-2021 | South African | Professional rugby union | COVID-19 | Respiratory tract | 185 players had 42 physician verified positive SARS-CoV-2 infections (I = 1.23; 95%CI: 0.86–1.61). Incidences during the three periods were: non-contact training = 0, contact training (I = 1.04; 0.36–1.71; mostly forwards), and competition (I = 1.54; 1.00–2.10). 83 % of the infected players were symptomatic and 52 % of the 42 positive players had systemic symptoms. Median return-to-training was 14 days. 22 (52 %) SARS-CoV-2 infections were rugbyrelated: 13 off-field (31 %), 9 on-field (21 %). 11 % of matches were cancelled due to SARS-CoV-2 infections. | Training appeared to be the most high-risk activity, with almost all of the on-field infected players being forwards (8 of 9 players). We suggest that this could be attributed to the scrum situation, where there are prolonged periods of close physical contact of up to 16 players, 19 requiring high energy expenditure, and therefore increased ventilation rates. | / | / | 245 |
| 2020 | UK | International cricket EURO 2020 Royal Ascot Download Festival Wimbledon tennis Goodwood Festival The Open Golf | COVID-19 | Respiratory tract | 1. In total, 6376 cases were identified as attending EURO 2020 football events at Wembley during the period they were likely to have acquired COVID-19, and 3036 during the period they were likely infectious. Numbers in both categories increased substantially at the later matches, especially the Final. A smaller number of cases were identified at other events, such as the All England Lawn Tennis Championships at Wimbledon where there were similar numbers of spectators and venue capacity, but the total numbers of potentially infectious (n = 299) or acquired cases (n = 582) were much lower. 2. Close to half of all cases associated with the Euros coming from this date. The total number of cases who attended the Wembley Semi Final (7th July) and Final during the period when they likely acquired their infection was both high at 2092 and 3404 respectively. The number of cases who attended the Final and were potentially infectious was 2295.  3. Cases identified at The Open Golf were 91% male, while at Wimbledon 52% were male.  4. There are higher proportions of events coded as pubs or bars on each of these dates compared to other dates for COVID-19 cases in England. | 1. Whilst the Wimbledon crowds were well managed and largely compliant with the required risk mitigation, the initial reports from research teams indicate that spectators at the Wembley stadium became less compliant with mitigation such as face coverings as the tournament progressed. 2. the risk of COVID transmission was not mitigated by the control measures in place for entry to the event itself. | 1. Previous crowd behaviours associated with football fans has underpinned the methods used to manage these crowds including the legislation in place governing alcohol consumption within football stadia. In general terms, this has the effect of concentrating people into as few areas as possible while crowd management strategies often hold groups until they can be moved en-masse in a controlled manner. To mitigate the risk of transmission of COVID-19, it would be preferable to dissipate the crowds across as wide an area as possible and manage the movement over long periods of time, as happened at other events including the Wimbledon tennis championships. 2. Transmission of infection may have occurred at the event itself or during any of the other reported activities associated with the event, of which attending a pub or restaurant is the most frequently reported. Neither full vaccination nor a negative LFD test will completely eliminate the possibility of an infectious individual attending an event, but it should reduce the likelihood of someone transmitting highly infectious amounts of virus to a large number of individuals attending the event. | We used contact tracing data routinely collected through telephone interviews and online questionnaires, to describe the potential public health impact of the large sporting and cultural events on potential transmission and incidence of COVID-19. Data from the EURO 2020 matches hosted at Wembley identified very high numbers of individuals who tested positive for COVID-19 and were traced through NHS Test & Trace. | 246 |
| 2021 | United States | NCAA (National Collegiate Athletic Association) | COVID-19 | Respiratory tract | / | / | Requiring face masks, restricting eating/drinking, reducing spectator capacity, encouraging physical distancing, and reserving space between groups of ticketed seats are strategies that attempt to reduce transmission risk. When vaccinations are not available or coverage is low, ensuring adherence to key transmission mitigation strategies such as mask wearing during mass events is critical during the current, and any future, pandemic. | Counts were recorded via a locally developed web-based app, MaskCount (Regenstrief Institute Inc). Pretesting using images of public mask-wearing behavior suggested high interobserver agreement (κ = 0.93). | 247 |
| 2020 | United States | NBA | COVID-19 | Respiratory tract | / | / | A fundamental question of the COVID-19 pandemic has been when it is safe to discontinue isolation precautions in patients who have recovered. Given that it appeared that the virus from these persistently positive individuals was not replication competent and there was minimal risk of transmission after sufficient time passed after symptom onset, the CDC shifted to a symptom-based strategy in July 2020, advising that isolation and precautions can be discontinued 10 days after symptom onset or a patient’s first positive RT-PCR result, unless the patient is immunocompromised or had severe disease. | / | 248 |
| 2022 | Qatar | the national professional league (Qatar Stars League, QSL) | COVID-19 | Respiratory tract | During the study period, 85 subjects returned positive (cycle threshold (cT) <=30) or reactive (30<cT<40) PCR tests, of which 36 were players. The infection rate was consistent with that of the general population during the same time period. More than half of infected subjects were asymptomatic, and the remaining had only mild symptoms with no one requiring hospitalisation. Symptom severity was associated with lower cT values. Social contacts and family were the most common sources of infection, and no infection could be traced to training or matches. Of the 36 infected players, 15 presented positive serology during the study period | / | Professional and leisure team sports were suspended as measures of public health in most countries to prevent the spread of SARS-CoV-2. There is limited information if contact sports played outdoors such as football (soccer) indeed represent an increased risk of viral transmission. | / | 249 |
| 2022 | China | Olympic | COVID‑19 | Respiratory tract | / | / | 1. Both the model simulation results and the actual observation show that most of the cases in the closed loop are "seed" cases, that is, the cases in the closed loop are negative in the customs examination. On the one hand, it shows that the customs ports are preventing and controlling the overseas epidemic 2. The first line of defense of input plays an important role; On the other hand, it shows that daily nucleic acid detection in closed loop can effectively control the risk of transmission. | / | 250 |
| 2022 | China | Olympic | COVID‑19 | Respiratory tract | / | 1. The Olympics inherently brought about a festive atmosphere to Japan, which had previously been strictly adhering to COVID-19 countermeasures. For example, many restaurants began ignoring operation hour curfews, likely due to the perceived double standard of hosting the Olympics during a state of emergency. 2. Finally, despite the lack of spectators and tourists in Tokyo during the Games, the human mobility during the Olympics was greater than during the previous, third state of emergency. In the midst of the rise of the more infectious Delta variant, insufficiently reduced levels of domestic mixing would directly increase the risk of exposure to COVID-19 among locals. | We examined the association between the hosting of the Tokyo Games and the daily number of COVID-19 cases in Japan by using a synthetic control method (SCM) to approximate the counterfactual trend in the daily number of COVID-19 cases, assuming the absence of the Games. | / | 251 |
| 2021 | Japan | Olympic | COVID‑19 | Respiratory tract | / | 1. As the government and scientific advisors heavily relied on public health and social measures (PHSMs), particularly people’s self-restrictive measures, the pandemic fatigue among citizens was more common, lessening the effectiveness of the state of emergency during Tokyo 2020. 2. In addition, along with the mobilisation of healthcare workers to support the Games, the health system outside the Olympic arena and village was overstretched, restricting citizens’ access to testing, and patients’ treatment in healthcare facilities.  3. Prediction of the surge of the Delta variant in Tokyo was not sufficiently reviewed, and the quarantine for international athletes and staff was not strengthened.  4. While the Olympic villages and some facilities were constructed in the preCOVID-19 era, scant investment in preventing the airborne infection of SARS-COV-2 was made.  5. lessons learned from the successful and challenging management of previous mass gathering events during the pandemic were poorly reflected. | 1. Although the vaccination status was not a requirement for participation in Tokyo 2020, it is expected that the combination of vaccine passports with a negative test result for COVID-19, as well as regular screening will be warranted, along with appropriate measures such as mask wearing. These measures should not only be applied to athletes and officials but to spectators. 2. Olympic and Paralympic Games organisers should collaborate with public health agencies and institutions globally to conduct rigorous health risk assessment through the all-hazard approach before the Games. - The host country should at least suppress the transmission of virus beforehand. - Regular screening is beneficial to quickly break the chains of virus transmission. - The surge capacity for the Games, and citizens’ access to testing and healthcare must be secured. This will facilitate proper risk communication and will promote community engagement. - Ensuring accountability and transparency in risk analysis, data collection and their reporting are vital to promote open scientific discussions and thus assist in managing games in a safe and secure manner. | / | 252 |
| 2021 2022 | China | the 2021 World Ski Championships the 2022 Beijing Olympic Winter Games | Multi-infectious disease | Respiratory tract | 1. We found only 6 cases of symptomatic ARI among 183 (3%) members of Team Finland during 2 major winter sports events (the 2021 World Ski Championships and the 2022 Beijing Olympic Winter Games). 2. The difference between these events and the historical comparison groups before COVID-19 is dramatic. 3. At the January 26–February 28, 2018, Olympic Winter Games in PyeongChang, South Korea, and the February 18–March 3, 2019, World Ski Championships in Seefeld, Austria, ARIs were recorded in 58 (33%) of the 174 members of Team Finland.  4. Clinically, all the ARIs were mild common colds. In the Oberstdorf and Beijing winter sport events, we detected only 3 (4%) non–SARS-CoV-2 infections (caused by 3 different viruses) in 73 athletes, and those infections did not spread further. Symptom onset was 1–3 days after arrival in Beijing, which suggests that the infections were acquired in Finland | / | In summary, our observations suggest that multilayered mitigation strategies effectively prevented respiratory viral infections during 2 major winter sport events that occurred during the COVID-19 pandemic. Sport events may be held without an increased risk for respiratory viral infections. ARIs are now returning, concurrent with relaxed control measures. It remains to be clarifed what mitigation procedures will be suffciently effective in preventing respiratory viral infections during major sports events after the COVID-19 pandemic while, at the same time, minimally affecting the well-being of the athletes. | / | 253 |
| 2021 | Japan | Olympics | COVID‑19 | Respiratory tract | / | / | 1. Preventive measures, adapted amid continuous expert review, included single hotel rooms for athletes, at least daily testing, and wearable technology for monitoring contacts, supported by rigorous contact tracing.  2. Establish Covid-19 advisory committees that include player associations, governing bodies, and experts; have a plan B to respond quickly to an outbreak 3. No forced waivers for athletes; full and comprehensive insurance for training and competition periods Safe international travel policies; sport-specific protective measures. 4. At least daily RT-PCR testing for athletes, which may be supplemented with lateral-flow antigen tests. 5. wearable technology for proximity monitoring, backed by human contact tracing; designated hotel isolation facilities. 6. Tailored treatment and rehabilitation programs; mandatory cardiac screening; access to on-the-ground mental health support. 7. Distribution of medically approved face masks, including filtering facepiece respirators for high-risk settings, such as buses. 8. Individual rooms for athletes; modified means of travel and access; capacity limits for all indoor environments, including cafeterias; proper ventilation and Covid-19 detection. | The IOC plans to provide every athlete with a smartphone that has mandatory contact-tracing and health-reporting apps. | 254 |
| 2022 | Qatar | FIFA 22 | malaria, arthropod, Leishmania infections | Vector | / | (1) a rise in the number of pathogen introduction events in response to a rapid increase of Qatari population (×3.8 over 15 years) due to high number of migrants that represent more than 75% of the population together with a considerable influx of tourists’ (×7.5 over 14 years). (2) the high proportion (more than 99%) and increase of urban population (×4.7 over 15 years), providing ideal conditions that increase the likelihood of VBD local transmissions. (3) the booming of freight air transport (×24.6 over 14 years), port traffic (+29% over 14 years), and merchandise import (×10.6over 14 years), which increases the risk of introduction of vectors, pathogen infected or not. | A number of recommendations are suggested in order to (1) strengthen the integrated vector management (including surveillance and control) and (2) ensure effective VBD outbreak preparedness and response, within a “One Health” approach, by complying with WHO resolutions: i. Define a national integrated strategy for the management of VBDs in Qatar, to be developed within a surveillance and control plan for the main VBDs at risk for the country, identify all contributors and their tasks, build a coordination unit, and allocate adequate financial resources. ii. Build capacities in entomology to increase knowledge of local vector ecology and competences for vector identification and vector surveillance. iii. Build capacities in research infrastructure including in laboratory diagnostic, for both molecular detection of pathogens and identification of vectors. iv. Develop coordination and enhance national and international collaborations to build intra- and inter-sectoral networking on vectors and pathogens they transmit. v. Build an integrated surveillance and control system of both vectors and pathogens, based on integrated vector management, with prioritised targets in term of vector species, pathogens, and high-risk areas. vi. Develop a national public health pesticide management policy to tackle weaknesses in pesticide use or management and to avoid and solve potential problems of impact on human health and nontarget organisms. vii. Plan an evaluation and quality check process to adjust the surveillance system when/where needed. | / | 255 |
| 2021-2022 | United States | National Basketball Association | COVID‑19 | Respiratory tract | Individuals who were fully boosted experienced 608 confirmed SARS-CoV-2 infections and were significantly less likely to be infected than fully vaccinated individuals who were booster eligible and had not received a booster, who had experienced 127 confirmed infections (adjusted HR, 0.43 [95% CI, 0.35-0.53], P < .001; Table 2). The secondary analyses evaluating symptomatic infection showed a similar association (adjusted HR, 0.39 [95% CI, 0.30-0.50]; P < .001). No hospitalizations or deaths occurred. Omicron was the dominant variant, representing 93% of 339 sequenced cases. | / | 1. Player vaccinations were not mandated. Staff were required to be fully vaccinated by October 1, 2021, and to have received a booster dose by January 5, 2022, if eligible. Masking requirements were similar between players and staff, with the exceptions of players unmasking on court and head coaches unmasking during games. 2. This study found that in a young, healthy, highly vaccinated cohort frequently monitored for SARS-CoV-2, booster vaccination was associated with a significant reduction in incident infections during the Omicron wave. | / | 256 |
| 2021 | Japan | Olympics | COVID‑19 | Respiratory tract | / | / | With a sensitivity about 91% and very high specificity, some rapid antigen testing may offer an acceptable alternative to RTqPCR to detect asymptomatic carriers at busy airports. One significant caveat is the fact that passengers must be advised to avoid eating drinking, gargling and smoking in advance and upon landing. Besides, appropriate instructions for saliva collection are necessary to standardize procedures. | / | 257 |
| 2019 |  | Nordic World Ski Championships |  |  | Ten out of 26 (38%) athletes, 6 out of 36 (17%) staff, and 3 out of 52 (6%) control subjects experienced symptoms of respiratory infection (p = 0.0013). The relative risks for acquiring symptomatic infection were 6.7 (95% confidence interval [CI], 2.1–21.0) of athletes and 2.9 (95% CI, 0.84–10.0) of the staff as compared to the controls. Asymptomatic infections were identified in 8%, 22%, and 19%, respectively (p = 0.30). The etiology of respiratory infections was detected in 84% of the cases. | It is commonly reported, but not well documented, that heavy exercise-induced immunosuppression, mental stress, nutritional restrictions, air travel, sleep disturbance, human crowding, housing with other athletes, low temperature with low humidity, and competition all increase the risk of respiratory virus infection, especially during the winter time when many viruses are prevalent. It is of note that the athletes had a 2-fold risk for illness when compared with the staff, who share many risk factors with the athletes. On the other hand, the staff had a 3-fold increased risk compared with the control subjects, suggesting that travelling and crowding are also important risk factors. It is tempting to link competition with heavy physical and mental stress and enhanced susceptibility to respiratory viral infections. | Molecular POCT is accurate in the hands of trained team physicians. | Fifteen respiratory viruses were searched for by molecular point-of-care test (POCT) on site. After the Championships, 16 respiratory viruses were tested for by 2 different multiplex PCR in laboratory-based testing.Two PCR based tests were used: 1) Allplex Respiratory Panels 1–3 (Seegene, Seoul, South Korea) for respiratory syncytial virus A and B, adenovirus, influenza A and B viruses, rhinovirus, enteroviruses, parainfluenza type 1–4 viruses, human coronaviruses 229E, OC43, and NL63, human bocavirus, and humanmetapneumovirus; 2) in-house triplex RT-PCR assay for respiratory syncytial virus, rhinoviruses, and enteroviruses. The viral shedding was calculated as reported earlier 28 specimens by FilmArray Panel on site, 336 by Allplex Panel, and 336 by triplex PCR panel in the laboratory. | 258 |
| 2022 | China | Winter Olympics | COVID‑19 | Respiratory tract | / | / | A total of 18 scenarios were designed regarding the NAT frequency, symptom monitoring, and strengthening close-contact control. An agent-based stochastic dynamic model was used to compare the cost-efectiveness of diferent NAT scenarios and optimize the surveillance strategies. The dynamics of the proposed model included the arrival and departure of agents, transmission of the disease according to Poisson processes, and quarantine of agents based on regular NATs and symptom onset. Accumulative infections, cost, and incremental cost-effectiveness ratio (ICER) were simulated in the frame of the model. ICER was used to compare the cost-effectiveness of diferent scenarios. Univariate sensitivity analysis was performed to test the robustness of the results. | A total of 18 scenarios were designed regarding the NAT frequency, symptom monitoring, and strengthening close-contact control. An agent-based stochastic dynamic model was used to compare the cost-efectiveness of diferent NAT scenarios and optimize the surveillance strategies. The dynamics of the proposed model included the arrival and departure of agents, transmission of the disease according to Poisson processes, and quarantine of agents based on regular NATs and symptom onset. Accumulative infections, cost, and incremental cost-efectiveness ratio (ICER) were simulated in the frame of the model. ICER was used to compare the cost-efectiveness of diferent scenarios. Univariate sensitivity analysis was performed to test the robustness of the results. | 259 |
| 2021 | Japan | Olympic | COVID‑19 | Respiratory tract | Overall, 11417 and 4403 athletes participated in the Olympic and Paralympic Games, respectively. Tokyo 2020 provided 1014170 screening tests for all participants, including athletes, team officials and stakeholders, during the Games. Screening detected 299 cases, which were later confirmed, including 53 athletes and team officials and 246 stakeholders (table 1). Overall, 869 cases were detected by airport quarantine, symptomatic cases and close contact tracing during the Tokyo 2020 Games | / | 1. Preventive measures for the Tokyo Games duration (1 July to 8 September 2021) were documented in the ‘playbook’ and distributed to all participants, including athletes and officials, international federations, Olympic/Paralympic family, marketing partners, broadcasters, press and workforce before the game.4 Participants were required to follow infection control measures, such as physical distancing, hand hygiene, masking and participation in a daily COVID-19 screening testing programme. 2. During the Games, IDCC organised (1) daily surveillance and reporting to the IOC, IPC and public health authorities; (2) large-scale screening tests for all the participants for SARS-CoV-2; (3) transportation of those suspected or confirmed of having infection; (4) medical isolation facilities for the athletes who tested positive for SARSCoV-2; and (5) investigation of SARSCoV-2-positive cases. 3. The daily screening test was specially tailored to minimise the impact on the Games. Sample submission timings were set to avoid overlap with competition. Athletes who tested positive were transported to a dedicated isolation hotel by IDCC-prepared vehicles with negative air pressure to avoid spreading infection and increasing local medical resource burden. At the isolation hotel, healthcare professionals monitored the patient’s health conditions daily to manage any medical needs immediately. 4. Athletes with COVID-19 were not permitted to participate in the Games. Athletes who recovered from COVID-19 were assessed by the Results Advisory Expert Group before they returned to play. Confirmed close contacts by regulation of the Ministry of Health, Labour and Welfare (MHLW) were immediately separated from the team to curb further transmission and tested. In the case of the other participants, IDCC communicated with the local government, and they were isolated by the local government. We believe that under strict implementation of these procedures, Infection control at mass gathering events requires close communication with the central and local health administration. IDCC worked directly with the Tokyo Metropolitan Government, the National Institute of Infectious Diseases and MHLW during the Games. All liaisons from each organisation worked in the same room for smooth communication. IDCC also coordinated with other organisations, including IOC/IPC, national Olympic and Paralympic Committees (NOC/NPC) and international sports federations. | / | 260 |
| 2020-2021 | Japan | Japan Judo Federation Medical Science Committee (AJJF) | COVID‑19 | Respiratory tract | Testing began in October 2020 and was conducted until September 2021 for 2,073 athletes over the duration of six tournaments. The SARS-CoV-2 virus was detected in 11 (0.29%) athletes. In tournaments held until April 2021, SARS-CoV-2 was detected in only one of the 1,173 (0.08%) athletes tested. However, prior to tournaments held from July 2021 onward, when variants became prevalent, SARS-CoV-2 was detected in 10 (1.1%) of the 900 athletes tested (p < 0.05). No clusters were reported in association with any tournament. | / | We believe that drafting health inventory forms two weeks prior to judo tournaments was essential and kept the participants alert. However, as variants emerged, some participants who were positive could not be detected through their inventory forms; this demonstrates the need for caution when relying on health inventory forms alone. | Infection prevention managers were installed prior to tournaments. Two weeks prior to each tournament, these managers drafted health inventory forms for athletes and related parties to check for COVID-19-associated symptoms. Although PCR testing prior to tournaments was not required by policy, the AJJF conducted them (directly and by mail) prior to six tournaments from October 2020 to September 2021 for athletes whose health inventory forms listed no symptoms. | 261 |
| 2020 | Japan | Olympic | COVID‑19 | Respiratory tract | A total of 12,072 foreign participants were enrolled, 13 (0.11%) of whom had a positive PCR test result. None of these cases were broadcasters or members of the press, were tested outside the Olympic Village, or had a history of COVID-19 infection. The effectiveness of full vaccination and vaccination at least once (14 days ago) was 74% (95% confidence interval [CI]: 6-93%) and 81% (95% CI: 30-95%), respectively. Three breakthrough infections with the Delta variant were observed in 6,485 fully vaccinated participants (0.05%). The positivity rate was 0.09% among adherents to the bubble system and 0.28% among nonadherents, but this difference was not significant. | / | vaccination, frequent testing, social distancing, and adherence to a bubble system. | / | 262 |
| 2012-2020 |  | Paralympic Summer and Winter Games | Multi-infectious disease | Respiratory tract/Gastrointestinal | Athletes with neurological impairments (n=51) lost 10 days per year due to respiratory problems (95% CI 9 to 11). Gastrointestinal problems caused a time loss of on average 4 days per year in athletes with neurological impairments versus 1 day in athletes with musculoskeletal impairments (mean difference 2.7 days, 2.1–3.3). | / | Respiratory tract and other infections; gastrointestinal problems,represented the greatest health burden. Our fndings can help guide the allocation of clinical resources, which should include a broad network of medical specialists, together with dieticians and physiotherapists, to meet the health challenges in Para athletes. | 1. We then informed candidate athletes (20–40 athletes each in the five Games preparation cycle) as well as their respective part-time health providers (typically one physician and three physiotherapists), about the procedures, risks and benefits of the Norwegian Olympic and Paralympic Health Monitoring Programme. The development and implementation of the programme have previously been described in detail. 2. We instructed athletes to report all health problems they had experienced in the preceding 7days, including ongoing problems reported earlier. Each week until final selection and the start of the Games, we collected health data from these athletes using an online questionnaire (Oslo Sports Trauma Research Center (OSTRC)-H/OSTRC-H2).24 25 The National Para team physician and physiotherapist followed up health problems reported. 3. In a few sports, where available through the sport federations’ resources, the follow-up was done by the team physiotherapist. The methodology for data collection, using three different electronic platforms, has been described in detail. Athletes were encouraged to report every health problem, irrespective of its consequences on their sports participation or performance and irrespective of whether they had sought medical attention | 263 |
| 2020 | Austria, England, Wales, Spain, Portugal, Northern Ireland, Scotland, Italy, Cyprus, South Africa, United Arab Emirates | European Tour golf events | COVID-19 | Respiratory tract | During the study period, there were 2900 RT-PCR tests performed on 195 professional golfers competing on the European Tour. Four players tested positive on-site during the study period (0.14% of tests; positive results were declared with Ct <40). Two positive tests were returned as part of routine protocols, while two reported a history of close contact with an individual who had tested positive for SARS-CoV-2 and were isolated and target tested. All were asymptomatic at time of testing, with three developing symptoms subsequently. None required hospital admission. There was no transmission from player to player. | Although playing golf represents an outdoor, low-population density activity, travel (especially transit through airports and shared public transport) and hotels likely carry more risk and require comprehensive risk mitigation. | Detection rate of SARS-CoV-2 among players in our study did not appear to be related to the national rates of detection in the host country. This confirms the success of the ‘tour bubble’ concept and the effectiveness of evidence-based, non-pharmaceutical interventions When a clear link was found, cases were typically due to shared indoor space including housing or car sharing. This is in keeping with the transmission routes in professional team sports where transmission was thought to be minimal during training or matches but shared indoor environments presented higher risk than outdoor environments. When positive cases were detected, strategies were effective in identifying the positive player, implementing individual isolation and tracing appropriate contacts. This achieved cessation of disease transmission within the ‘tour bubble’. It is therefore clear that when appropriate mitigating protocols are adhered to, competitive golf can be conducted internationally with low rates of SARS-CoV-2 transmission. | Daily symptom and temperature checks and weekly reverse transcriptase PCR (RT-PCR) screening were performed to determine potential carriage of SARSCoV-2. Onset and type of symptomology were analysed. Gene expression and cycle thresholds (Cts) were reviewed for all positive cases. Repeat PCR testing was performed on all positive players. RT-PCR analysis included human housekeeping genes and various RNA genes specifc for SARS-CoV-2. | 264 |
| 2019-2021 | Germany | men’s professional football leagues in Germany | COVID-19 | Respiratory tract | / | / | Non-match-specific situations, such as goal celebrations, are an important part of football and constitute up to 33% of the player pairs’ contact time; limiting such situations may further reduce contacts between players. | Contacts between players were defined as being within a two-metre radius during matches and were differentiated as either match-specific or non-match-specific. | 265 |
| 2022 | China | Winter Olympics | COVID-19 | Respiratory tract | / | / | To ensure athletes’ health, safety, and ability to compete, the organizers have introduced the “closed-loop management system” or the COVID-free “bubble”, in which COVID-free athletes could stay and be protected from potential infections that could upend their Games plans (International Olympic Committee, 2021), if not their career as well. To shed light on the issue, based on Beijing, 2022 Olympic Playbooks and most up-to-date guidance issued, this article and its accompanying infographic were developed to illustrate factors that could influence athletes’ ability to join and stay in the “bubble”, participate in the Games, and further build their career. Furthermore, we also adapted and integrated easy-to-adopt mental health de-stress techniques recommended by the World Health Organization to help athletes better thrive amid the Beijing, 2022 Winter Olympics, in or outside of the “bubble” | / | 266 |
| 2022 | Qatar | FIFA 22 | COVID-19, monkeypox, Marburg, cholera and polio rhinovirus and coronaviruses | Respiratory tract/Gastrointestinal | / | Monkeypox has previous signifcant fatality rates of about 5% and may cause many health complications, including fatal outcomes in 2022. Moreover, unlike COVID-19, the world is not yet ready to control the infection. Vaccines against it are not yet available in Qatar or many other nations, and education and prevention programmes for monkeypox are urgently needed. | 1. Regarding COVID-19, vaccination and testing are effective ways to reduce the risk of its spread. Therefore, it would be right only to allow fully vaccinated adults to visit Qatar. Even as imposed in many countries, booster doses will be essential to require them. Those who had the last dose of their vaccine more than six months back must be asked to get a booster dose for COVID-19. Else, they must show proof of a negative RT-PCR test for SARS-CoV-2 taken 72 hours before the journey. Qatar should also ensure that all high-risk individuals get a booster dose before the start of the event. 2. For monkeypox, there is a need for mass training of the medical, paramedical staff, and all others involved in event management. That would help identify or trace the infection early and thus isolate patients. | / | 267 |
| 2020 | United States | Multi-sports | COVID-19 | Respiratory tract | Players within leagues using bubbles demonstrated a statistically significant (P , 0.001) lower PR (0.09 per 1000 [95% CI 5 0.018, 0.26]) and incidence (1.81 per 1000 [0.37, 5.29]), than players within non-bubble leagues (0.92 [0.79, 1.06] & 50.87 [43.79, 58.78] respectively). Post-Bubble PRs (NHL: 0.00 [-,0.18]; NBA: 0.31 [0.038, 1.12]; MLS 0.14 [0.004, 0.79]) and incidence (NHL: 0.00 [-, 5.07]; NBA: 5.78 [0.70, 20.88]; MLS: 1.71 [0.043, 9.54]) were significantly lower than Pre-Bubble rates respectively (NHL: 2.71 [1.85, 3.83]; NBA: 20.80 [14.99, 28.12]; MLS: 2.14 [1.31, 3.30] & NHL 40.00 [27.36, 56.47]; NBA: 122.1 [87.99, 165.0]; MLS: 29.94 [18.29, 46.24]). PRs were significantly lower within leagues inside bubbles than within their respective host state/province. Prevalence did not show a statistically significant difference when comparing Bubble versus Non-Bubble or PreBubble versus Post-Bubble, but was significantly higher within bubble leagues than their respective host state/province. | / | Quarantine bubbles significantly reduce incidence and PR of COVID-19 within team-based professional sports when compared to general population and control group statistics. Prevalence may not be a good indicator of a bubble’s success as it includes pre- and post-bubble cases and does not account for asymptomatic carriers who are undiagnosed in the community but test positive within our studied professional athletes due to semi-daily testing. | / | 268 |
| 2010-2016 | United States | Rugby | Respiratory illness, Digestive illness | Respiratory tract/ Gastrointestinal | The IR of acute illness was signifcantly lower in the I (5.5: 4.7 to 6.4) versus the C period (13.2: 9.7 to 18.0) (p<0.001). The IR of respiratory (C=8.6: 6.3 to 11.7; I=3.8: 3.3 to 4.3) (p<0.0001), digestive (C=2.5: 1.8 to 3.6; I=1.1: 0.8 to 1.4) (p<0.001), skin and subcutaneous tissue illness (C=0.7: 0.4 to 1.4; I=0.3: 0.2 to 0.5) (p=0.0238), all infections (C=8.4: 5.9 to 11.9; I=4.3: 3.7 to 4.9) (p<0.001) and illness burden (C=9.2: 6.8 to 12.5; I=5.7: 4.1 to 7.8) (p=0.0314) were signifcantly lower in the I versus the C period. | / | A Team Illness Prevention Strategy (TIPS) can reduce the overall incidence of illness by 59% during a sports tournament. | / | 269 |
| 2020, 2022 | China, Japan | Olympics | COVID-19 | Respiratory tract | / | The COVID-19 risk factors include crowd density, duration of the events, method of transportation, distance between individual members of the audients, distance between athletic participants, and the ongoing changing status of COVID-19 pandemic. | The Tokyo Olympic Committee recommended four main principles: Mask wearing; COVID-19test, trace, andisolate;Minimizing physical interaction; and Think hygiene. The Beijing Olympic Committee utilized the following strategies: Vaccination; Closed loop; COVID-19 Liaison Officers; Test, trace and isolate; Minimise physical interaction and Think hygiene | / | 270 |
| 2020 | Italy | skiing competitive races | COVID-19 | Respiratory tract | One thousand three hundred and ninety (91.6%) out of 1,518 participants were accredited. Only one of the 694 swab tests performed on-site for accreditation turned positive in an asymptomatic subject and was confrmed by molecular swab test. During the events no suspected case of COVID-19 was reported to the LOC. At the end of the two sporting events, antigen swabs were performed on 50 participants, and these yielded negative results. No COVID-19 positive case was reported in the next 14 days after the end of the games. | / | The application of a protocol based on a self-administered questionnaire with a combination of antigen tests was eﬀective for the reopening of elite skiing activity. | / | 271 |
|  |  |  | ACUTE RESPIRATORY VIRAL INFECTIONS, | Respiratory tract | / | / | 1. In determining the level of quarantine and observation measures, it should be borne in mind that the eﬀects of coronavirus COVID-19 on the human body on average are: 80% – mild symptoms that are not signifcantly life-threatening; 14 – serious problems, pneumonia, difculty breathing; 5% – critical conditions – respiratory failure, septic shock; 2% – deaths. It is very important to use an electronic platform called the “Hazard determination and risk assessment systems’’ (HDRAS) to determine the extent of an epidemic to public health 2. It is very important to use an electronic platform called the “Hazard determination and risk assessment systems’’ (HDRAS) to determine the extent of an epidemic to public health. It allows you to analyze information about dangerous factors from various sources of information, including the media (media), as well as social networks and more. Te system used by experts of the European Regional Ofce to collect information on infections and other factors during mass events, including sports and the subsequent provision of advisory services, including the Chief Sanitary Inspectorate in Poland. 3. If there coronaviruses infection and quarantine and observational measures necessary to follow with the provisions for the mass sporting events organization: - to provide close contact at the national level for mass sports events organizers with the institutions of the national public health system, and at the international level, if necessary, with WHO experts and the WHO Regional Ofce for Europe (the latter for European countries); - in planning for the mass sporting events organization in conditions of coronavirus epidemic it is necessary to take into account the general state of public health regarding the availability of coronavirus infection and quarantine and the application of observation measures for the territories and settlements (cities, etc.) where the mass sports events planned; - if necessary to ensure timely evacuation of athletes from quarantine zones, as their presence in these areas can not only be dangerous to their health, but also aﬀect the quality of training, competitive process; - use the “Hazard determination and risk assessment systems” (HDRAS) to assess the danger degree of the epidemiological situation regarding coronavirus infection; - to use diagnostic test methods of coronavirus infection to assess the epidemiological situation and to determine the athlete’s health state; - do not to allow spectators and fans from quarantine areas to places of mass sports events; - to maximum limit as much as possible the presence of elderly people and children at sports events as high-risk groups for coronavirus infections; - do not to allow domestic and farm animals as possible sources of coronavirus infections on the territory and in the premises where sports events are held; - to perform general hygiene requirements, hands cleaning and disinfection, dressing protective face masks and its timely replacement with new ones and so on. | / | 272 |
| 2021 | Japan | Tokyo Olympic Games | COVID-19 | Respiratory tract | / | / | 1.Comprehensive COVID-19 testing.  2.Athletes, support staff and others may only be in their official hotel or village, on official transport vehicles, or at an official venue, and cannot venture out into the city or even to official venues other than the one in which they compete or work.  3.Athletes are required to depart Tokyo at the latest 2 days after their sport competition has concluded. | / | 273 |
| 2020 | Germany | 2nd division professional basketball in Germany | COVID-19 | Respiratory tract | SARS-CoV-2 was detected in 65% of the tested participants at a median of 4.00 days after the sporting event. Positively tested participants developed symptoms 4.00 days after the sporting event. | / | 1.Appropriate protective equipment is able to prevent SARS-CoV-2 transmission and that particle filter masks are especially useful in this regard. 2.Carry out PCR tests for detecting possible asymptomatic SARS-CoV-2 infections amongst actively participating players especially prior to indoor sporting events in order to prevent the mass transmission of this disease. | / | 274 |
| 2020 | Denmark | two best football (soccer) leagues for men in Denmark | COVID-19 | Respiratory tract | The incidence rate of players testing positive for SARS-CoV-2 was 0.53% (4/748). Among the players, one club had two, two clubs had one, and 23 had no positive cases. | / | 1. Players with typical symptoms of COVID-19 were advised to immediately self-isolate and follow the latest recommendations and guidelines from the Danish health authorities, contact their own general practitioner and follow the doctor's instructions. 2. Self-reported health checks for all onpitch staff were recommended, and players were instructed to complete a daily health questionnaire focusing on signs of SARS-CoV-2 infection. If a player had one or more symptoms (temperature >37.5 Celsius, sore throat, dry cough, headache, nausea, diarrhea, muscle pain, respiratory symptoms, or other symptoms), the player should report to the team's medical staff and was then allowed to train or play matches only if given clearance by the medical staff. 3. A testing procedure was implemented for SARS-CoV-2 in asymptomatic on-pitch staff and players. | / | 275 |
| 2021 | Japan | the Olympic and Paralympic Games 2021 in Japan | COVID-19 | Respiratory tract | / | / | Japan achieved a rapid increase in its COVID-19 vaccination rate after May 2021. The government strategy for COVID-19 vaccination and evidence-based press reporting resulted in rapid achievement of a high Japanese vaccination rate. | / | 276 |
| 2021 | Japan | the Tokyo Olympic games | COVID-19 | Respiratory tract | / | / | / | **The COVID-19 athlete passport:** The passport presents a novel way of managing elite athletes, whereby they retain an electronic document (for example, on their phone or through an official IOC app) that reports their status regarding previous exposure to the virus, testing, results and vaccination. The system could be integrated so that the team manager/doctor also have access to the information of all athletes under their care. From this, appropriate precautions can be tailored to each athlete during their travel and stay in Tokyo, and risk can be stratified in the unfortunate event of an on-site COVID-19 exposure or outbreak. | 277 |
| 2020 | Global | the Olympic Games and FIFA World Cup | COVID-19 | Respiratory tract | / | The covid-19 pandemic has shone a spotlight on global inequities facing migrant populations, with major health and social effects on migrants. Emerging evidence suggests that migrant populations, including labour migrants, in high income countries may have been disproportionately represented in covid-19 cases, admissions to hospital, and deaths. | The international focus of big sporting events should be used to hold all actors, particularly host countries, international sporting organisations, and industries, responsible to take action to protect the health of labour migrants and ensure their access to health and vaccination systems, alongside catalysing a renewed focus on data collection, research, and evidence based policy making in this neglected area. | / | 278 |
| 2021 | Italy | The European Soccer Championship (EURO) | COVID-19 | Respiratory tract | / | 1.Large spontaneous celebrations across the country after the national team won the final match. Informal celebrations became more and more common across the country until 11 July 2021, when a ‘frenzy night’ of celebrations took place in many Italian cities. The change in behaviour was widespread enough to determine a rapid and almost synchronous increase in transmission across the country with the highest peaks in the two weeks following the final match. 2.An increase in the number of new cases of SARS-CoV-2 infection in July 2021, mainly among younger males that usually hold a greater interest in this type of event. 3.The cases and clusters we were able to link retrospectively with EURO2020 were associated with aggregations in private settings, in pubs/other public buildings, or in public squares. | Several factors could have contributed to limiting transmission inside the stadium.  1.Ongoing COVID-19 restrictions including indoor or outdoor mask wearing and physical distancing were in force.  2.A strict COVID-19 security protocol at the stadium itself during the championship, that limited live attendance at 25% of total stadium capacity, established access verification and managed spectator flows.  3.All spectators older than six accessed the stadium only if they exhibited proof of at least one of the following: negative SARS-CoV-2 test performed within the previous 48 hours, vaccination (at least one dose), prior SARS-CoV-2 infection ended within the previous 6 months.  4. Cross flows and overcrowding at the entrance were limited by assigning spectators in advance specific entrances with travel directions | / | 279 |
| 2021 | Japan | the Tokyo Olympic and Paralympic Games | COVID-19 | Respiratory tract | / | / | 1.During training and games, all support staff were also required to wear masks and gloves and to disinfect the cooling devices used. In addition, all drinks for the athletes were prepared individually for each athlete, and other infection control measures were taken according to the characteristics of each competition. 2.During training, each athlete had a separate drink holder for hydration, and measures were taken to avoid sharing between athletes. | / | 280 |
| 2021 | Japan | the Tokyo Olympic games | COVID-19 | Respiratory tract | A model of that integrated source–environment–receptor pathways was developed.A noprevention scenario produced 1.5–1.7 newly infected individuals per infector entry, whereas a combination of cooperative preventive measures by organizers and the spectators achieved a 99% risk reduction, corresponding to 0.009–0.012 newly infected individuals per infector entry. | / | Seven possible and pragmatic measures were considered: (a) physical distancing of spectators at entrances and exits, (b) decontamination of surfaces in concessions, (c) enhanced stadium air ventilation, (d) partitioning of spectators in the stands, (e) mandatory face masks at the concourses, restrooms, and concessions, (f) hand washing with soap in restrooms, and (g) wearing hats or other headwear in the stands. | / | 281 |
| 2021-2022 | Japan; China | (1) the Tokyo Olympic Games; (2) the Beijing Olympic Games | COVID-19 | Respiratory tract | There were 33 COVID-19- positive athletes, 34 cases in team officials and 417 confirmed close contacts; only 7 of whom tested positive (<2%). 96% (Tokyo) and 95% (Beijing) of close contacts, who were isolated and monitored, but not placed in quarantine hotels, continued to train and compete. The genomic sequencing of positive cases showed that there was no evidence of spread between international and domestic populations. The local subvariants were not discovered in countries beyond Japan or China after the Olympic Game. | / | 1.Include public health expertise on sport governing body and host medical committees.  2.Develop a predetermined risk-based pandemic mitigation strategy with contingency planning that is communicated in advance of the event to all stakeholders.  3.Ensure provision of financial and logistical resources across all participants to support the strategy.  4. Apply public health principles that have proven effective (handwashing, masks, physical distancing, vaccination).  5. Facilitate individual responsibility by educating event participants on public health principles relating to infectious disease mitigation (prevention, detection, management).  6.Ensure adequate access to training, nutrition, mental and physical health support of participants in isolation.  7. Evaluate the effectiveness of COVID-19 mitigation protocols with robust scientific rigour. | / | 282 |
| 2022 | South Africa | two large professional golf events in South Africa | COVID-19 | Respiratory tract | There was a total of 378 player-week episodes and 378 caddie-week episodes during the study period. Three persons tested positive while registered at events during the study period (0.4% of person episodes). The positive tests were returned from two players and one caddie, all of which were asymptomatic at the time of testing. There was one high-risk contact who consistently returned negative antigen tests. There was no evidence of transmission. | / | Protocols to permit asymptomatic, vaccinated persons positive for SARS-CoV-2. 1. Optimising the use of outdoor space. 2. Advice regarding social distancing. 3. Mask use in shared indoor space and where social distancing is not possible. 4. Maximal ventilation of indoor areas. 5. Daily checking of symptoms and contact history. 6. Encouragement of vaccine uptake to players and caddies, and mandate of vaccine to all others on-site. 7.Rapid antigen testing was performed on entry into the event and an RT-PCR was performed if players later developed symptoms. Each day, a symptom and contact history checklists were completed online through an application prior to admission to the event. | / | 283 |
| 2021 | Italy | the Volleyball Nations League (VNL) | COVID-19 | Respiratory tract | There were 54 participants from Japan, including 27 male and female staff and players each. There was one team doctor for both men and women.There were 9 fever cases from our men’s team, but all of them tested negative for COVID-19. Overall, a total of 10,170 tests were performed and only one was positive. | / | 1. Basic hygiene management included wearing masks, maintaining social distancing, frequent hand washing and disinfection, and air purification through ventilation.  2.No one was allowed to leave the bubble, except for transport to the hotel and activity venues, which was allowed only by designated means of transportation (mainly buses), and going out for other reasons was not allowed.  3.In the designated hotel, only trips to the private beach for relaxation were allowed. Each team was assigned a specific time to take their meals. Although buffet style, all meals were served by the restaurant staff. In the hotel, we tried to avoid contact with other teams as much as possible, including in the elevators. The person-in-charge was required to send a daily health report of the entire team to the task force platform. 4.PCR test before departure. | / | 284 |
| 2020 | Qatar | the Asian Federation Cup (AFC) Champions League-West | COVID-19 | Respiratory tract | The outbreakinvolved approximately 74 COVID-19 cases and constituted the main subject to communicate the risks of the infection from Sep 11th – Oct 4th, 2020. | / | / | / | 285 |
| 2020-2021 | Greece | Super League Greece | COVID-19 | Respiratory tract | In total, 190 positive cases (130 among professional football players and 60 among staff) were recorded. Out of the 190 cases that turned positive, 64 (34%) cases were considered as symptomatic, and 126 (66%) cases were asymptomatic. The incidence rate of a positive test result for footballers was 0.57% (confidence interval (CI) 0.48–0.68%) and for staff members it was 0.27% (CI 0.20%, 0.34%), respectively. | Footballers recorded a twofold increased risk of COVID-19 infection in comparison to staff members (relative risk = 2.16; 95% CI = 1.59–2.93) | The implementation of a weekly diagnostic testing (RT-PCR) was critical to break the transmission chain of COVID-19, especially among asymptomatic football players and staff members. | / | 286 |
| 2021 | Japan | Tokyo Olympic and Paralympic Games | COVID-19 | Respiratory tract | / | 1.The temporary increase in the resident population volume due to the influx of people into Tokyo for purposes other than watching the Games during the consecutive holidays and summer vacation during the 2020 Tokyo Olympics may increase the population density. Japan does not have administrative measures such as lockdown. 2.If the domestic vaccination pace (about 120,000 per day) continues and that the population volume would increase by about 5%, which is about the same as in August 2020, the national analysis team has indicated that an explosive outbreak of infection could occur from late July to mid- August. | 1.Suspension of acceptance of overseas guests by the TOC; 2.Include domestic spectators with a maximum of 10,000 people within 50% of the capacity of the venue in principle; 3.Implementing Transportation Demand Management; 4.Thorough prevention of elderly clusters and active vaccination; 5.Detect infected people at their early stage and supply medical resources quickly before deterioration including strengthening of the telephone consultation system; 6.A part of the metropolitan hospital was converted into a specialised hospital to prepare surge capacity, and strengthened the information sharing system for hospitalisation management. For asymptomatic positive or mild patients at designated accommodation facilities (hotels) or home, the health status monitoring system by telephone and IT application has been strengthened and oxygen concentrators are being installed (hotels).  7.The system of immediate visit home by medical team is also established to cope with the event of patient’s sudden deterioration. | / | 287 |
| 2021 | Japan | Tokyo Olympics | COVID-19 | Respiratory tract | / | Up towards 100,000 visitors arriving from all over the globe, with every conceivable SARS-CoV-2 variant, known or hitherto unknown; | 1.Full immunisation of all athletes, companions, support staff, press and other visitors; 2.Predeparture PCR testing for SARS-CoV-2 before boarding flights and systematic PCR screening of participant at entry port followed by a ten day period of close follow-up. | / | 288 |
| 2020 | Germany | the German Bundesliga (football (soccer)) | COVID-19 | Respiratory tract | Suspicious symptoms were reported for one player. Of 1702 regularly tested individuals (1079 players, 623 officials members), 8 players and 4 officials tested positive during one of the first rounds of PCR testing prior to the onset of team training, 2 players during the third round. Nine players converted from negative/borderline to positive (without symptoms); two players who initially tested positive tested negative at the end of the season. 22 players remained seropositive throughout the season. | / | / | / | 289 |
| 2020 | the United States | the 2020 National Football League (NFL) season | COVID-19 | Respiratory tract | A total of 632 370 tests administered across 11 668 persons identified 270 (2.4%) COVID-19 cases from 1 August to 14 November 2020. | / | / | The U.S. National Football League (NFL) and the NFL Players Association (NFLPA) instituted a large-scale COVID-19 monitoring program involving daily testing using 2 reverse transcription polymerase chain reaction (RT-PCR) platforms (Roche cobas and Thermo Fisher QuantStudio), a transcriptionmediated amplification platform (Hologic Panther), and an antigen point-of-care (aPOC) test (Quidel Sofia). | 290 |
| 2020 | Australia | Women’s National Basketball League (WNBL) 2020 season | COVID-19 | Respiratory tract | / | / | When sport resumed, many leagues implemented hubs, or ‘bubbles’, to minimize risk of infection between athletes and the community. |  | 291 |
| 2021 | Tunisia | Tunisian Premier League | COVID-19 | Respiratory tract | Among the 1388 players of the Tunisian first professional league, 102 players (7.35%) had COVID- 19. Three were excluded for lack of clinical data.The most frequent clinical manifestations were anosmia, agueusia and muscle fatigue. The clinical examination was normal in all the subjects included except for the presence of fever among 37 (37.9%) subjects. Two players presented a pericardial effusion without signs of severity. |  | / | / | 292 |
| 2020-2021 | the United States | National Football League 2020 Season | COVID-19 | Respiratory tract | A total of 256 players had confirmed COVID-19 cases in the testing period, with a range of 1 to 22 player cases per team. |  | The NFL and the National Football League Players Association (NFLPA) implemented protocols designed to mitigate spread of infection. Protocols included daily testing, use of proximity trackers, restrictions on facility access and interactions among players and staff, mandatory masking, restrictions on gathering for meetings and meals, disinfection and decontamination guidelines, improvements in ventilation system filtration and air flow, quarantine and isolation, contract tracing, and modifications to travel activities throughout the season. | / | 293 |
| 2020-2021 | France | French Federation of Football (FFF) championships 2020/21 season (league 1 and league 2) | COVID-19 | Respiratory tract | 47% of all professional football players (572/1217) tested positive for SARS-CoV-2 by nasopharyngeal PCR. The proportion of players infected by SARS-CoV-2 was similar in both leagues with 45% (n = 276/613) in league 1, and 49% (n = 296/604) in league 2. Among all players infected by SARS-CoV-2, most individuals had one single episode, except for 11 players who had documented reinfections (11/572, 1.9% of all SARS-CoV-2 infections). No hospital admission and no death were related to COVID-19 during the study period. |  | The FFF developed a COVID-19 protocol starting in August 2020, by the beginning of the 2020– 2021 season. That protocol consisted in mandatory masking outside of sports activities, physical distancing, regular washing with alcohol-based hand rub, facility disinfection, closed-door matches (i.e., with no public), and repeated SARS-CoV-2 PCR testing through nasopharyngeal swabs. Every team had a physician in charge of the protocol and its adherence by players and staff members. Players and staff members of all teams were tested by nasopharyngeal SARS-CoV-2 RT-PCR one to three days before every game. Tests before games were performed systematically, for each player, whether or not they were symptomatic. When a person tested positive, a quarantine period was started for a minimum of 10 days, and a negative PCR was needed to end the quarantine. Not respecting physical distancing (being at a distance of <1 m) during a period longer than 15 min or exposition to biological fluids of a person with COVID-19 led to an exposure questionnaire, a quarantine and an immediate PCR testing. | / | 294 |
| 2020-2021 | Spain | the LaLiga Smartbank (second division of the Spanish professional football league) | COVID-19 | Respiratory tract | In a team with twenty-six professional football players, 14 players (53.8% of the total sample) were diagnosed with COVID- 19 infection while the remaining 12 players never tested positive in any antigen or PCR test. From the sample of players with a recorded COVID-19 infection, five players (35.7%) did not report any symptoms while the remaining nine players reported one or several symptoms such as fatigue, asthenia, fever and headache. No player had to be admitted to hospital due to COVID-19 infection |  | Following the guidelines provided by Spanish football and medical authorities, all players were tested for SARS-CoV-2 infection using a rapid antigen test four times per week and by Polymerase Chain Reaction (PCR) analysis 48 h before any official match for the duration of the season. In those cases of a positive rapid antigen test, infection by SARS-CoV-2 was confirmed by a PCR analysis. Once a positive case of COVID-19 was reported in a football player, the medical staff of the team recorded the date of diagnosis, the symptoms suffered by the player during the infection, the quarantine time set and the duration of the illness, including the dates of return to training and competition. The medical staff considered that a player had overcome the infection once they had had two negative PCR tests over a 24-h period. | / | 295 |
| 2021 | the United States | National Football League 2021 Season | COVID-19 | Respiratory tract | During December 12, 2021–January 1, 2022, a surge in COVID-19 cases occurred among NFL players and staff, with an average of 336 cases per week, compared with 30 cases per week during the preceding 3 months. Sixty-six (53%) cases occurred among 125 unvaccinated persons, three (23%) among 13 partially vaccinated persons, and 924 (14%) among 6,443 fully vaccinated persons. Among 117 (12%) cases with a sequenced virus isolate during this period, 111 (95%) were classified as Omicron and six (5%) as the SARS-CoV-2 B.1.617.2 (Delta) variant. |  | Fully vaccinated persons were tested weekly and unvaccinated persons were tested daily by RT-PCR. Daily testing was requested for all fully vaccinated persons with COVID-19, who could be released from isolation if they were asymptomatic or fever-free with symptoms improved for 24 hours with medical staff clearance, and receipt of two negative or Ct≥35 RT-PCR test results (point of care [POC] or laboratory-based) within 24 hours. | / | 296 |
| 2021 | Japan | the Tokyo Olympic and Paralympic Games | COVID-19 | Respiratory tract | SARS-CoV-2 RNA was detected in 151 wastewater samples (41.9%), of which 53 (26.4%) and 98 (61.6%) were from the Olympics and Paralympics, respectively. The observed concentrations of SARS-CoV-2 RNA in passive samples were up to 35 000 copies per sampler. |  | / | / | 297 |
| 2021 | Japan | the Tokyo Olympic and Paralympic Games | COVID-19 | Respiratory tract | SARS-CoV-2 RNA was detected in a total of 233(33.8%) waste water samples consisting of 151(41.8%) and 82(24.9%) samples from passive and grab sampling, respectively. |  | / | / | 298 |
| 2021 | Japan | the Tokyo Olympic and Paralympic Games | COVID-19 | Respiratory tract |  |  | 1.Wearing face mask at all times, washing hands frequently, reducing physical interactions, maintaining social distance and cancelling public attendance were applied and monitored. 2.The new position of CLO (COVID-19 Liaison officer) was created to help each team with the pandemic related issues.  3. Epidemic prevention bubbles were established and all the participants were restricted in Olympic bubbles to restrict the interaction with non-participants to a minimum level.  4.The countermeasures that lasted from 14 days before the arrival, through the whole journey in Japan and the departure were all mentioned clearly in the playbook.  5.The personal protective knowledge, the collecting process of samples and the customs clearance process were recorded as short videos for delegation members.  6.Individual fitness rooms or treatment rooms were established to reduce the possibility of cross infection.  7. Vaccination against COVID-19 proved to be effective. | / | 299 |
| 2020 | the United States | National Basketball Association (NBA) 2020 Regular and Postseason | COVID-19 | Respiratory tract | Among 3648 individuals who participated, 36 (1%) were persistent positive cases. Antibodies were detected in 33 individuals (91.7%); all remained asymptomatic following the index persistent positive RT-PCR result.Cases were monitored for up to 100 days, during which there were at least 1480 person-days of direct exposure activities, with no transmission events or secondary infections of SARS-CoV-2 detected (0 new cases). |  | / | / | 300 |
| 2020 | the United States | The National Football League (NFL) 2020 football season | COVID-19 | Respiratory tract | Over the course of the monitoring period (August 9– November 21), 623,000 RT-PCR tests were performed among approximately 11,400 players and staff members; 329 (approximately 2.9%) laboratory-confirmed cases of COVID-19 were identified. After intake screening, in August and early September, fewer than 10 COVID-19 cases were identified per week for the following 7 weeks. However, during September 27–October 10, a total of 41 cases were identified among players and staff members. |  | The NFL-NFLPA implemented a standard COVID-19 mitigation protocol in July that included mandatory masking; physical distancing; frequent handwashing; facility disinfection; restricted facility access; and regular, frequent testing of players and staff members. RT-PCR tests, with results available in 24 hours, were initially conducted 6 days per week for players and most staff members. | / | 301 |
| 2020 | Germany | the German Bundesliga | COVID-19 | Respiratory tract | Seroprevalence (NT used as confirmation) in 2164 samples from 1184 players and staff was rather similar in May (23/1157, 1.99%) and June (21/1007, 2.09%). All participants were PCR-negative during the study period. |  | / | / | 302 |
| 2021 | Japan | the Tokyo Olympic and Paralympic Games | COVID-19 | Respiratory tract | A multitype branching process model predicted: 1. If prevention measures were well observed, the number of new cases among Games accredited individuals would approach zero by the end of the Games.  2. If transmission was not controlled our model indicated hundreds of Games accredited individuals would become infected and daily incidence in Tokyo would reach upwards of 4,000 cases.  3. Had domestic spectators been allowed (at 50% venue capacity), over 250 spectators might have arrived infected to Tokyo venues, potentially generating more than 300 additional secondary infections while in Tokyo/at the Games. |  | / | / | 303 |
| 2022 | China | the Beijing 2022 Winter Olympics and Winter Paralympics | COVID-19 | Respiratory tract | 1. A dynamic model with pulse detection and isolation effect predicted: the peak of the epidemic reached on the 57th day. During the simulation period (100 days), 13,382 people infected COVID-19. The mean and peak values of hospitalized cases were 2650 and 6746, respectively. 2.According to the official data of the events, from January 23, 2022, to the closing of the Winter Paralympics on March 13, 2022, a total of 16,092 Olympic-related personnel entered the country through the airport, with a total of 284 people testing positive at the airport. Moreover, 2,546,100 nucleic acid tests were conducted in the closed-loop, with 179 positive people testing positive for a total of 463 cases. However, there was no cluster epidemic spread in the closed-loop. |  | 1. The most important measures to stop COVID-19 transmission during the event were daily nucleic acid testing, reducing contact among people, and daily health monitoring; 2. Strictly implementing the entry policy and reducing the number of cases entering the closed-loop system could delay the peak of the epidemic and provide time for medical resources to be mobilized. | / | 304 |
| 2018 | Indonesia | the 2018 Asian Games | Multi-infectious diseases | Gastrointestinal/Respiratory tract | Among a total of 782 South Korean elite athletes, 209 (26.72%) illnesses were reported.The most common system involved was gastrointestinal (n=93, 44.49%), followed by respiratory (n=53, 25.36%).Out of 93 athletes complaining of gastrointestinal problems, 83 of them reported gastrointestinal problems associated with diarrhoea/vomiting, 16 athletes had gastrointestinal problems associated with fever, and 42 athletes had associated pain in the abdomen. Among 53 athletes with respiratory symptoms, 46, 15 and 13 presented with throat pain, fever and dyspnoea, respectively. Infection was attributed to be the cause in 50 (94.33%) athletes. | The maximum incidence of illness was reported in table tennis (100%), followed by rowing (94.44%). The risk of illness was also high in archery, baseball, basketball, canoeing, equestrian, fencing, field hockey, handball, tennis, sailing, shooting, squash and swimming (30%–60% of registered athletes in each sport). | / | / | 305 |
| 2020 | the United States | The National Football League (NFL) 2020 season | COVID-19 | Respiratory tract |  | Most (n = 849; 43.2%) distinct contacts among players were pre-game, 546 (27.8%) were during competition, and 569 (29%) were post-game. | A robust prevention program integrating masking, distancing, hygiene, and ventilation when off-field can be created to minimize on- and off-field exposures, which effectively reduces transmission risk in outdoors and/or well-ventilated stadium settings. | / | 306 |
| 2021 | Japan | the Tokyo Olympic Games | COVID-19 | Respiratory tract | 1.The estimated overall accumulated infections can be approximately rounded to 32 infected athletes in the studied period from July 1 to July 23, 2021. This would imply that the United States and Great Britain possess the highest risk of bringing multiple infected athletes into Tokyo along with Spain, Russia, Netherlands, Brazil, and Argentina.There would still be 11 unidentified cases after three antigen COVID-19 tests if the first test was taken coincidently on the day of infection.  2.Athletes infected: two South African football players, three beach volleyball players, four Czech Republican players, and five Dutch players. |  | / | / | 307 |
| 2021 | the United Kingdom | the EURO 2020 football championship events | COVID-19 | Respiratory tract | 2,632 cases were identified who self-reported attendance at EURO 2020-related events. The peak in cases occurred on 21 June 2021, 3 days after the England vs Scotland match at the Wembley Stadium (Figure 1) and declined gradually following Scotland’s elimination from the championship on 22 June 2021. Approximately 4% of the 63,874 new SARS-CoV-2 cases reported in Scotland from 11 June to 7 July were related to EURO 2020. Travelling to London was reported by 61% of the 2,632 cases. Secondary attack rates were significantly higher for EURO 2020 index cases (27.2%). | 1.90% of the positive cases stemming from these events were male and 73% were aged 20–39 years. 2. The steep increase in cases probably occurred as a result of more frequent social gatherings surrounding the EURO 2020 matches, rather than from official EURO 2020 events. | / | / | 308 |
| 2022 | China | the Beijing 2022 Winter Olympics Games | COVID-19 | Respiratory tract |  | Mass gatherings during large-scale sporting events represent a conducive environment for viral transmission given the close, prolonged, and frequent interactions between participants. | 1.Classification of participants: in consideration of the activities and locations, we classified the participants in the Games as belonging to high-risk, moderate- risk, and low-risk populations. 2.Classification of venues: all 15 categories of sports at the Beijing 2022 Olympics were classified as belonging to the relatively high-, moderate-, and low-risk categories with respect to COVID-19 considerations. Non-competition areas, such as the Olympic Villages, contracted hotels and other designated hotels, designated hospital/ isolation facilities, dedicated transport, and opening/closing/ victory ceremonies, were also categorized based on the risk of SARS-CoV-2 transmission. 3.Common countermeasures: (1) Restricted in the closed loop throughout the stay in China; (2) Fully vaccinated (Validation of receiving a COVID-19 booster vaccination for all local workforce entering the closed loop); (3) Keeping in touch with CLO; (4) Body temperature monitoring; (5) Daily COVID-19 PCR screening test; (6) Properly wearing a face mask; (7) Ensuring physical distancing; (8) Droplet prevention measures; (9) Specialized isolation space; (10) Staggered coming and going; (11) Shields and transparent barriers to enforce unidirectional flow; (12) Regular ventilation and disinfection of the venues. | / | 309 |
| 2021 | Japan | the Tokyo Olympic Games | COVID-19 | Respiratory tract | More than 320 cases have been linked to the Olympic Games. New daily positive cases (DPC) in Tokyo have decreased significantly since the end of August 2021. After the peak DPC of 5773 cases was reached on August 13, 2021, new DPCs rapidly decreased to 219 at the end of September. |  | / | / | 310 |
| 2019 | England | the Vitality Netball World Cup 2019 | Multi-infectious diseases | Gastrointestinal/Respiratory tract | A total of 11 illnesses in 11 players were reported calculating to an illness incidence of 7.64/1000 player-days (95%CI: 3.12–12.15) and period prevalence of 5.72%. The most common specific diagnosis of illness was upper respiratory tract infection (n = 4; 36.36%), followed by gastroenteritis and dysmenorrhea (n = 2; 18.18% each). |  | / | / | 311 |
| 2020 | the United Kingdom | Super Rugby League matches | COVID-19 | Respiratory tract | Out of 136 total players, there were 8 SARS-CoV- 2 positive players, 28 players identified as increased-risk contacts and 100 other players in the matches. Within 14 days of the match, one increased-risk contact and five players returned positive SARS-CoV- 2 reverse transcriptase PCR (RT-PCR) tests, and 27 increased-risk contacts and 95 other participants returned negative SARS-CoV- 2 RT-PCR tests. |  | / | / | 312 |
| 2013-2017 | South Africa | the Super Rugby Union tournaments | acute respiratory tract illness | Respiratory tract | The incidence rate of respiratory tract infections was 0.4 (0.3–0.6)/1000 player-days. For sub-categories the highest incidence rate was in upper respiratory tract infections (1.9; 1.7–2.2), while the % illness causing time-loss was influenza-like illness (100%), lower respiratory tract infections (91.7%), infective sinusitis (55.6%), and upper respiratory tract infections (49.0%). Illness burden was highest for upper respiratory tract infections (2.0; 1.6–2.5), and thedays until return-to-play/single illness was highest for lower respiratory tract infections (3.2; 2.3–4.4), and influenza-like illness (2.1; 1.6–2.8). |  | / | / | 313 |
| 2022 | Qatar | the 2022 FIFA Men’s World Cup | COVID-19 | Respiratory tract |  |  | Health centres can be established near the stadiums, providing ease and accessibility for public vaccination. Healthcare workers should also be at the forefront of this initiative to harness trust in vaccination. Stalls can be placed by healthcare authorities near stadium entrances for dissemination of brochures and answering public questions related to vaccine safety. Mobile vaccination clinics near the stadium can provide a strategic chance to encourage on-site vaccinations, and an opportunity to vaccinate or provide booster shots to those who do not have ready access to vaccines. Further incentives to motivate football fans in the form of pro-vaccination sporting merchandise can foster a constant source of public interest on this topic.Social media awareness campaigns tailored to football followers with key messages and interviews by players during the tournament could collectively inspire vaccine confidence. | / | 314 |
| 2021 | Japan | Tokyo olympics | COVID-19 | Respiratory tract |  | COVID-19 is still circulating in Japan with around 5000 to 6000 new cases per day, while only 1,000 to 1,500 new cases were recorded at the time the event was planned in 2020 (Fig. 1). The Japanese authorities announced that it would expand and extend a state of emergency in Tokyo through May 31 due to the ongoing coronavirus pandemic. In addition, only 1% of the population has received COVID- 19 vaccination in Japan to date. | 1.Allowing individuals already immune to the virus (either by vaccination or after recovery) to participate to mass gatherings should be carefully considered. 2.Quarantine of participants at home for a reasonable period of time following the event should also be considered. 3. Systematic screening of participant for SARS-CoV-2 carriage by PCR or antigenic test should be considered when possible. Participants should strictly adhere to individual preventive measures such as face mask use, hand hygiene and social distancing. Finally, real-time surveillance of SARS-CoV-2 circulation among participants will be of primary importance to detect outbreaks at mass gathering events. | / | 315 |
| 2021 | Japan | Tokyo Olympic and Paralympic Games | COVID-19 | Respiratory tract |  |  | / | The National Institute of Infectious Diseases, Japan, undertook enhanced event-based surveillance (EBS) for infectious diseases occurring overseas that have potential for importation (excluding coronavirus disease 2019 [COVID-19]) for the Tokyo 2020 Olympic and Paralympic Summer Games (the Games). The pre-existing EBS system was enhanced using the World Health Organization Epidemic Intelligence from Open Sources system and the BlueDot Epidemic Intelligence platform.  Overall, 140 events and 20 diseases were identified by the enhanced EBS system during the provisional period of 11 July to 8 August 2021. A total of 17 events and 10 diseases were detected by the pre-existing system, 121 events and 11 diseases by the EIOS system, and two events and two diseases by the BlueDot platform. The median number of events per day was 5 (range, 1–9). All identified events were evaluated for risk, with none meeting the high-risk criteria for publishing in the daily report. | 316 |
| 2021 | Japan | the Tokyo Olympic Games | COVID-19 | Respiratory tract |  |  | 1.The IOC required stringent countermeasures such as frequent reverse transcription-polymerase chain reaction testing of participants and limiting interaction with people beyond Olympic venues.  2.Both international and domestic spectators were banned to avoid transmission within venues and to minimize physical contact between the domestic population and Games participants. 3.Prohibiting restaurants and bars from serving alcohol and requiring them to curtail their operations. 4.Measures to control SARS-CoV-2 transmission among the domestic population (i.e., banning spectators and imposing a stringent state of emergency lasting more than 8 weeks) during the Tokyo Olympic Games would be essential to bring the prevalence of severe COVID-19 cases within the limits of existing intensive care unit capacity in Tokyo. | / | 317 |
| 2020 | Brazil | Under-20, Under-23, São Paulo Cup, São Paulo Championship (Divisions I, II and III), Women’s São Paulo Championship and Women’s Under-17 | COVID-19 | Respiratory tract | Among 662 PCR-confirmed cases, 501 were athletes and 161 were staff. The new infection rate was 11.7% and 7.2% for athletes and staff, respectively. Athletes were more susceptible to infection than staff (OR: 1.71, 95% CI: 1.42, 2.06, p<0.001), although with lower chance for moderate to severe disease (OR: 0.06, 95% CI: 0.01, 0.54, p=0.012). Six teams had ≥20 individuals testing positive for SARS-CoV- 2, whereas 19 teams had ≥10 confirmed cases. Twenty-five mass outbreaks were identified (≥5 infections within a team in a 2-week period). | 1.Athletes were more susceptible to infection than staff (OR: 1.71, 95% CI: 1.42, 2.06, p<0.001); 2.Among players, the odds of infection increased according to age (OR: 1.05, 95% CI: 1.03 to 1.07, p<0.001) but did not differ between sexes (OR: 1.27, 95% CI: 0.95 to 1.71, p=0.113). | The São Paulo Football Federation (SPFF) protocol for reopening football involved repeated reverse transcriptase (RT)-PCR testing, temperature checks, social distancing, wearing a mask outside training and matches, strict hygiene and limited number of staff in the stadium. Athletes and staff were tested on a weekly basis. Each club’s responsible medic was obliged to submit confirmation of the negative test, or in the case of a positive test evidence that the 10-day quarantine had been completed, to the SPFF medical committee at least 24 hours before the match. Only players and staff who had confirmation of alignment with these procedures were permitted to participate. Immediate isolation of positive cases and tracing of close contacts were also recommended | / | 318 |
| 2020 | Qatar | the Amir Cup final (football competition) | COVID-19 | Respiratory tract | A total of 16,171 spectators undertook rapid antigen and antibody tests. Fifteen Spectators (n = 15) returned with a positive result for COVID-19 infection during the final event (positivity rate = 0.12%). All players underwent RT-PCR testing 48 h before the match. None of the players tested positive for COVID-19 infections. 1311 individuals reported having symptoms related to COVID-19 post final of Amir Cup. These spectators were tested for COVID-19 RT-PCR with an overall positivity rate (positive/reactive) to be 0.42% (69/16171). |  | 1.The ticket allocations were limited to 50% of the overall capacity of the venue/stadiums in Qatar.  2.Before being admitted inside the stadium, social distance, temperature screening, and mask-wearing were required (non-pharmaceutical interventions, or NPIs). All spectators intended to witness the final had to show either a negative COVID-19 test or a positive COVID-19 antibody test. Temporary testing centres for SARS-CoV-2 rapid antigen test were put up at the football clubs and in an adjacent conference centre in Doha 72 h before the event.  3.Standard precautionary methods were followed at the stadium: (1) Social distancing measures by reducing the overall numbers of spectators up to 50% of stadium capacity; (2) Having a green status (COVID negative) in the geolocalization tracking app; (3) Initial health screening procedures such as temperature and symptom (e.g., cough, shortness of breath, fever, chills, sore throat, headache, etc.); (4) Mandatory wearing of a mask at all times; (5) Fans seated on alternate seats (spacing between the two spectators was considered at least 1.5 m in front, behind, and diagonally to avoid crowding); (6) All high touch surfaces inside the stadiums were disinfected daily, and multiple mobile handwash stations and automated hand sanitiser dispensers were added to designated areas of stadiums. | Monitoring of post‑event infections among spectators: the antigen test and monitoring findings were recorded using a geo-locating cell phone application (EHTERAZ) and an online booking system. | 319 |
| 2021 | Japan | the Tokyo Olympic Games | COVID-19 | Respiratory tract | Synthetic control method (SCMs) were used to estimate the impact of holding the Tokyo Olympics on the number of newly confirmed COVID-19 cases in Tokyo (Japan). Holding the Olympics significantly increased the daily average of new COVID-19 cases by 105 to 132 cases in Tokyo (47 to 65 cases in Japan) per one million people from July 23 to August 22 compared to the counterfactuals. If the Olympics had not been held, the average daily number of COVID-19 cases could have been reduced by as many as approximately 1500 to 1850 cases in Tokyo and approximately 5900 to 8150 cases in Japan as a whole during that time period. We may therefore reasonably conclude that holding the Tokyo Olympics was likely a factor in the spread of COVID- 19 infection in the host city of Tokyo. |  |  |  | 320 |
| 2018-2019 | Italy, Australia | 2018 and 2019 Foiling Week editions held in Malcesine (Italy) and the Moth World Championship held in Perth (Australia) in December 2019 | upper respiratory tract illness | Respiratory tract | Among 77 sailors from 13 nationalities, most of the reported illnesses (80%, n = 4) were upper respiratory tract infections with an incidence of 0.51/1000 sailor-hours and a prevalence in the week of the regatta of 5.2%. |  |  |  | 321 |
| 2010 | Canada | the 2010 Olympic Winter Games | measles | Respiratory tract | A dualgenotype outbreak of measles occurring after the XXI Olympic Winter Games. In the context of an outbreak associated with a mass gathering—likely the result of 2 separate importations of MV from Olympics visitors. |  |  |  | 322 |
| 2022 | Qatar | the FIFA World Cup 2022 | Monkeypox | Respiratory tract/Contact |  | Monkeypox can spread from one person to another through close contact with respiratory droplets, infected lesions, body fluids, and contaminated materials (like clothing), all of which can happen in any mass gatherings (MGs), including the FIFA WC2022. It has been reported that Monkeypox virus transmission occurs primarily through sexual contact. Monkeypox has a long incubation period, and some patients are asymptomatic during the early stages of the disease, which can make case identification and isolation difficult. | 1.We stress the importance of raising awareness on the measures to reduce exposure risk and promptly recognizing and acting on situations of exposure or infection. This requires the establishment of reliable communication channels between health authorities, as well as visitors and local population. These communication channels should be available in different languages and via various means, such as through mobile application, website, phone line, and printed materials included into the welcome packages, to ensure optimal and easy access to various subgroups of the population. The communication channels should help establish a relationship of trust in the professionalism of the healthcare system and highlight the impartiality of healthcare workers. Visitors should not feel reluctant to seek diagnosis or care for fear of being judged or discriminated against.  2. Healthcare providers should raise travelers’ awareness of the importance of taking precautions to avoid sexually transmitted infections, especially since Monkeypox virus is typically diagnosed as a co-infection with other STIs. The communication channels should be bilaterally efficient and should be readily available to individuals in the event of an outbreak or case of exposure. Those with suspicion of MPX should be sent materials, be guided to screening and safety, and be considered for contact tracing. Travelers visiting the FIFA WC2022 should cooperate with the event organizers regarding health communication and preventive health measures. 3. Vaccination: Ring vaccination; post-exposure prophylactic immunization. |  | 323 |
| 2021 | Germany | The UEFA EURO 2020 tournament (EURO 2020) | COVID-19 | Respiratory tract | In total, five cases who had attended a football match in the stadium of Munich, nine cases, who attended a football match in a stadium outside of Germany, and 123 cases in association with public viewing events were identified by enhanced surveillance. |  |  | **Enhanced surveillance activities** included that each case within the observation period that might have had an association with the EURO 2020 was additionally marked with a specific identifier. At a pre-event consultation between national (RKI) and state authorities the nation-wide use of three different identifiers was agreed on to allow the distinction between cases notified in the context of attending a football match within a stadium in Munich, within another stadium in another host city and cases reporting attendance of a public viewing event anywhere. Public viewing event was defined as group gathering in a bar, restaurant or any other public place to watch a EURO 2020 football match broadcast. Furthermore, any cases in the context of privately organised group gatherings to watch a EURO 2020 football match, who were identified by case investigation of the local PH authorities and marked with the defined identifier for public viewing events, were additionally defined as cases in the context of public viewing events in this analysis. Information about the use of identifiers was distributed by the respective state PH offices to their local PH authorities. At RKI, data were regularly screened for any identifier-marked cases. A summary of reported cases was shared with the states. To Bavaria, the state hosting the football matches, a detailed description of cases being marked with the agreed identifiers was reported twice a week. | 324 |
| 2018 | the Republic of Korea | the PyeongChang 2018 Olympics | gastroenteritis (noroviruses) | Gastrointestinal tract | An outbreak of norovirus infections was recognized in the PyeongChang Winter Olympics, starting with security staff on 3 February 2018.Rectal swab samples (707) from food handlers were collected and examined for noroviruses by using real-time reverse transcription PCR and conventional reverse transcription PCR. Five of 707 samples were identified as noroviruses. Genotypes of the norovirus-positive samples were determined with sequencing analysis. Identified genotypes of norovirus in asymptomatic food handlers included GI.3, GII.4, and GII.17. The GII.17 strain was prevalent among the genotypes, accounting for three of five detections. | The occurrence of NoV infection in food handlers had a close relationship with environmental contamination in Gangwon-do. | Food handlers with noroviruses detected in rectal swabs were excluded from cooking, and all food handled by infected food handlers was discarded. Surveillance of norovirus infection for food handlers contributed to preventing norovirus spread. |  | 325 |
| 2020 | the United States | North American professional sports leagues (Major League Baseball, Major League Soccer, National Basketball Association, National Football League and National Hockey league) | COVID-19 | Respiratory tract |  |  | **All the times:** (A) Health planning 1.Maintain exposure-based groupings: Create and maintain risk of exposure-based categories encompassing each set of individuals necessary to operate, facilitate and participate in the event. 2.Maintain physical distancing protocols: Maintain strict physical distancing protocols that are tailored to the location, size, type of sport, type of venue (eg, indoor or outdoor6) and each exposure-based tier. 3.Require use of personal protective equipment in appropriate settings. 4.In consultation with medical advisors, implement an enhanced testing programme. 5.Monitor local conditions. (B)Sport participants 1.Communicate with participants. 2.Provide prevention supplies. 3.Promote the daily practice of everyday preventive actions. (C) Sport facilities 1.Clean and disinfect facilities. **Before a sporting event:** (A) Health planning 1.Establish relationships with key community partners and stakeholders. 2.Assess local healthcare capacity. 3.Consult with public health and infectious disease specialists. (B)Sport participants 1.Significantly reduce the number of attendees relative to who would ordinarily attend.  2.Establish protocols for pre-event screening.  3.Administer educational/training sessions. 4.Offer athletes and team staff access to the influenza vaccine. 5.Plan for unexpected staff absences. 6.Plan for responses after COVID-19 infection. (C) Sport facilities 1.Repurpose facilities and accommodation spaces and establish a controlled environment. **During a sporting event:** 1.Enforce adherence to preventive measures. 2.Establish secure processes to record health information to promote athlete health and safety. 3.Prohibit use of certain equipment or facilities. 4.Limit shared materials. 5.Explore alternative ways for fans, media and other observers to experience the sporting event and remotely or virtually interact with tier 1 participants. 6.Use a protocol and physical spaces to isolate, test and/or treat (or remove) any participants who become infected or symptomatic. 7.Use a protocol to guide the continued operation of a sporting event in the event a participant is exposed to COVID-19. **After a sporting event:** 1.Consult with staff, participants, partners, and local and state officials to evaluate the effectiveness of operational and communication plans. |  | 326 |
| 2011-2016 | the United States | Major League Baseball (MLB) and Minor League Baseball (MiLB) | Multi-infectious diseases | Gastrointestinal/Respiratory tract/Contact | The most common diagnosis was nonspecific viral illness (15.3%), followed by gastroenteritis (13.6%), other gastrointestinal illness (8.3%), influenza (7.0%), and upper respiratory infection (6.2%). Appendicitis (15.2%) and Epstein–Barr virus/cytomegalovirus (9.1%)were the most common season-ending diagnoses. |  | Prevention efforts should focus on limiting the spread of communicable viral, respiratory, and gastrointestinal disease among players, as the majority of diagnoses fell into these categories. Dermatologic infections were a relatively common cause of extended time out of play and season-ending illness, suggesting that continued progress in early identification and management of these conditions is warranted. | A Health and Injury Tracking System (HITS) was created to track player injuries and medical illness across all Major and Minor League affiliates. | 327 |
| 2021 | Japan | Tokyo Olympic and Paralympic Games | COVID-19 | Respiratory tract |  | 1. Japan likely missed COVID-19 cases because of testing limitations. 2.To early June, only 7% of the population has been fully vaccinated. | 1. The absence of international spectators, decreasing crowding in the Olympic Village, standard physical distancing, pre-departure and daily testing of athletes, and limiting athletes’ arrivals to 5 days before, and departure within 48 hours of, competition will all provide some protection. 2. Provide free vaccination for Olympic athletes. 3.While athletes will undergo daily COVID-19 salivary testing (rapid antigen-based, with PCR confirmation of positive rapid tests), it is proposed that all 11 000 athletes will gather daily at a dedicated testing area in the Village to ensure testing. 4.Games attendees are required to download the Japanese Contact Confirming Application (COCOA) smartphone application (app). | / | 328 |
| 2020 | Russia | the Russian Premier-League (RPL) 2020 season (soccer) | COVID-19 | Respiratory tract | COVID-19 infection was detected in 103 soccer players during COVID-19 screening. This number comprises 14.5% of all soccer players on the rosters of RPL soccer teams and is subjected to regular COVID-19 testing. The asymptomatic course was observed in 43.7% of cases (n = 45). In 56.3% of patients (n = 58), fatigue, headache, fever, and anosmia were the most common symptoms. Most cases had a mild course and did not impair return to regular exercise. Pulmonary lesions were detected in 36.2% (n = 21) of symptomatic soccer players. And in 23.3% (n = 24) of players with positive test results (in 3 cases, pulmonary lesions were revealed in asymptomatic players. In asymptomatic patients, "frosted glass opacities" extended less than 10% of the lung. Only two players were hospitalized with lung lesions and returned to regular sports. |  | According to the Russian quarantine rules, all individuals who tested positive for COVID-19 have to be isolated for 14 days regardless of their clinical symptoms. The quarantine can be lifted only after receiving two negative PCR-test results performed within 24 hours. | / | 329 |
| 2020 | the United Kingdom | British Horseracing events and Point to Point (PTP) grassroots races | COVID-19 | Respiratory tract | There were 7 reported COVID-19 diagnoses from 959 responses across these events (0.07%), with 2 in British Horseracing events (0.04%), and 5 in PTP races. |  | 1.From June 1, 2020, horseracing fixtures resumed in England, using a “behind closed doors” model. Sport “behind closed doors” involves fixtures with limited attendance and enhanced registration, thereby establishing a known number and role for all individuals expected on site. This can facilitate the identification of higher footfall areas (ie, those frequented by many attendees or staff groups, such as between changing rooms and parade rings), and the implementation of processes such as one-way systems, staggered arrival times, and social distancing in areas where more staff may be present throughout an event. 2.Restrict attendance from high COVID-19 incidence areas regionally. 3.The identification and minimization of crowded areas, managing flow in indoor areas, enhanced cleaning, development of spectator codes of conduct, use of Social Distancing Officers, hand sanitizer availability at prominent locations such as on entry to toilets, near taps or catering areas, implementation of cleaning teams, and movement planning for all groups on site were some of the processes undertaken for each event which may have minimized transmission risks for attendees. | / | 330 |
| 2022 | Qatar | the FIFA World Cup 2022 | COVID-19 | Respiratory tract |  |  | 1.Upon stadium entry social distancing, temperature screening, and mask wearing were required. The antigen test and monitoring results were recorded using a geolocating cell phone app and an online booking system. 2.Governments should continue to support vaccine campaigns until COVID-19 is declared endemic, in addition to encouraging individuals to take personal precautions that are probably the most cost-effective and reliable way to stay airborne virus/COVID-19 free and enjoy safe, and cheerful football again. | / | 331 |
| 2021 | Netherlands, Azerbaijan, Romania, Hungary, Denmark, Scotland, England, Germany, Italy, Russia, Spain | EURO2020 football championship | COVID-19 | Respiratory tract | A general increase in Covid-19 positivity trend in Europe was observed following a week of EURO2020 matches across most countries and host cities. A percentage increase in Covid-19 was observed in the host city/region as follows: Netherlands (1629%), Denmark (210%), Scotland (57%), England (382%), Germany (9%), Italy (104%), Russia (196%) and Spain (135%). Of note, Munich-Germany experienced a minimal increase in Covid-19 incidence while no increase incidence was observed in Budapest-Hungary (−23%) from the onset of Euro2020. | The EURO2020 was the first-time spectators were allowed to enter stadiums in Europe. Stadiums instituted several mitigations to safeguard the spectators although reports of transmission were still present. The major challenges were the gatherings outside the stadiums that might have contributed to these observations. Targeted restrictions might be required during mass sport events especially in the presence of highly transmissible variant(s) and low vaccination rates among the young generation. | / | / | 332 |
[truncated: 97,520 more chars]
